# Glutamate activates the MAPK pathway by inhibiting *LPAR1* expression and promotes anlotinib resistance in thyroid cancer

**DOI:** 10.1007/s12672-025-02853-0

**Published:** 2025-06-13

**Authors:** Bin Liu, Ying Peng, Yanjun Su, Chang Diao, Liansheng Cha, Ruochuan Cheng

**Affiliations:** 1 Thyroid Disease Diagnosis and Treatment Center, First Affiliated Hospital of Kunming Medical University, No.295 Xichang Road, Wuhua District, Kunming, 650032 Yunnan China; 2 Kunming Medical University, Yunnan, 650032 China; 3 Zhenxiong County People’s Hospital, Zhaotong , 657216 Yunnan China

**Keywords:** Thyroid cancer, Anlotinib, Drug resistance, Glutamate

## Abstract

**Objective:**

To investigate the effects of glutamate on thyroid cancer (TC) cell lines and TC-anlotinib-resistant cell lines and to explore the potential molecular mechanism of glutamate and *LPAR1* in promoting anlotinib resistance in TC.

**Methods:**

Glutamate was used to treat TC cell lines and TC-anlotinib-resistant cell lines, and changes in cell function and effects on the expression of *LPAR1* and MAPK pathway-related proteins were assessed. In addition, overexpressed-*LPAR1*. *(OE-LPAR1)* cell lines were constructed, and OE-LPAR1 and glutamate were combined with TC cell lines and TC-anlotinib-resistant cell lines to explore the interaction between glutamate and LPAR1. Finally, a xenograft tumor model was established in nude mice, and the protein expression of key nodes was detected for further verification.

**Results:**

Glutamate promoted the migration, invasion and proliferation of TC cell lines and TC-anlotinib-resistant cell lines, inhibited the expression of LPAR1, and promoted the expression of MAPK pathway-related proteins, whereas OE-LPAR1 had the opposite effect. Furthermore, glutamate promoted the expression of Ki67, inhibited apoptosis, significantly inhibited the expression of LPAR1, and promoted the expression of MAPK pathway-related proteins in a nude mouse xenograft tumor model, whereas OE-LPAR1 significantly inhibited the expression of Ki67 and promoted apoptosis.

**Conclusion:**

Our study revealed that glutamate promotes the progression of malignant biological behavior in TC cell lines and TC-anlotinib-resistant cell lines. Additionally, glutamate may activate the MAPK pathway by inhibiting the expression of LPAR1, thereby promoting resistance to anlotinib in TC.

**Supplementary Information:**

The online version contains supplementary material available at 10.1007/s12672-025-02853-0.

## Introduction

The overall treatment outcome for thyroid cancer (TC) is favorable [1], yet the mortality rate for advanced TC, particularly locally advanced and iodine-refractory/metastatic poorly differentiated thyroid carcinoma (PDTC) and anaplastic thyroid carcinoma (ATC), remains high. Reports indicate that the average survival times for PDTC and ATC patients are 3.2 years and 6 months, respectively [2, 3]. For this advanced stage of TC, targeted therapy is the primary treatment method [4]. Anlotinib, a novel tyrosine kinase inhibitor [5], is currently one of the most frequently used targeted drugs for the treatment of advanced TC in China owing to its favorable clinical outcomes and relatively low price, and it is included in the scope of medical insurance. In preclinical trials, anlotinib has demonstrated effective antitumor activity against various transplanted tumors [6]. Additionally, it has been incorporated into preoperative neoadjuvant therapy [7, 8]. Despite its widely recognized role and clinical effectiveness in treating TC, nearly half of patients do not experience significant therapeutic benefits in the later stages [9]. The suboptimal clinical outcomes for patients in the later stages are attributed to the emergence of drug resistance. Overcoming drug resistance represents a significant breakthrough in targeted therapy for advanced TC. To achieve this goal, understanding the mechanisms underlying drug resistance is crucial.

Anlotinib was initially widely used in patients with MTC and achieved good therapeutic results [8, 10]. Therefore, we selected the TT cell line for metabolomic and transcriptomic sequencing in our previous study and used it as the cell line for in vivo experiments in this study. Currently, anlotinib is also used clinically for differentiated thyroid cancer and ATC. So we selected the K1 and 8305 cell lines for further verification.Our previous metabolome sequencing suggested an abnormally increased differentially abundant metabolite glutamate (glu) while transcriptome sequencing suggested an abnormally decreased differential gene *LPAR1* [10].Moreover, by performing a biological information analysis, we found that both glutamate and *LPAR1* are associated with the MAPK signaling pathway [11–13]. Therefore, in this study we examined the hypothesis that glutamate activates the MAPK pathway by inhibiting the expression of *LPAR1*, thereby promoting TC-anlotinib resistance.

## Methods and materials

### Cell invasion and cell migration assays

The K1 (CellCook, CC2301), TT (Pricella, CL-0457) and 8305 C (Pricella, CL-0613) cells(all 3 cell lines were identified)were collected via trypsin digestion, counted, and seeded into a Transwell chamber with 400 µL of cell suspension (concentration of 1 × 10^5^ cells/mL) for incubation for 48 h. Subsequently, the Transwell chamber was removed, and the cells were fixed with a multipin fixator for 30 min at room temperature. Finally, the cells were stained with 500 µL of crystal violet solution and photographed at 100x magnification. For the invasion assay, Matrigel was evenly applied to the Transwell membrane.

### Flow cytometry analysis of the cell cycle and apoptosis

The cells were collected through conventional digestion and washed with precooled PBS prior to precipitation. To the cell pellet, 100 µL of 1× binding buffer working solution was added. Excluding the blank wells and PI control wells, 5 µL of FITC dye was added to each tube for apoptosis detection. Additionally, 5 µL of PI dye was added to each tube, excluding the blank wells and FITC control wells. The cells were then incubated in the dark at room temperature before detection. For periodic detection, the cells were fixed with precooled 70% ethanol and stained with propidium iodide before being incubated in the dark.

Transfection of *LPAR1*-overexpressing lentiviral vectors

The *LPAR1* overexpression plasmid and empty control plasmid were purchased from Invitrogen/Thermo Fisher Scientific. Before transfection, TT, K1, and 8305c cells were seeded into 6-well plates (1.0 × 10^5^ cells per well) and allowed to grow for 24 h. Lipofectamine 3000 (Invitrogen/Thermo Fisher Scientific) was used for transient transfection via standard procedures. Then,2.5 µg OE-*LPAR1* vectors, control, negative control (NC), or Lipofectamine 3000 was separately added to the serum-free medium and incubated at 25 °C for 10 min. Each group was mixed with Lipofectamine 3000 and cultured in serum-free DMEM. After 6 h of culture, the medium was replaced with DMEM containing 10% fetal bovine serum (FBS). These cells were then collected for further use.

### Western blot (WB) analysis

Protein samples were separated by sodium dodecyl sulfate-polyacrylamide gel electrophoresis (SDS-PAGE) and subsequently transferred onto polyvinylidene difluoride (PVDF) membranes. The membranes were blocked with 5% non-fat milk and incubated overnight at 4 °C with primary antibodies against target proteins. After washing, the membranes were incubated with horseradish peroxidase (HRP)-conjugated secondary antibodies and washed three times with phosphate-buffered saline (PBS). Protein signals were visualized using an ultrasensitive multifunctional imaging system and quantified by densitometric analysis with ImageJ software.Details of all antibodies are provided in the Table 1.

**Table 1:** Details of all antibodies

Antibody	Manufacturer	Catalog number	Dilution
LPAR1	Proteintech	20442-1-AP	1:1000
β-Actin	Proteintech	66009-1-Ig	1:25000
ERK	Affinity	AF0155	1:5000
MEK	Affinity	AF6385	1:2000
Phospho-ERK (p-ERK)	Affinity	AF1015	1:2000
Phospho-MEK (p-MEK)	Affinity	AF8035	1:3000
Ki-67	Affinity	AF0198	1:2000
β-Actin (duplicate entry)	Proteintech	66009-1-Ig	1:25000
HRP-conjugated goat anti-rabbit IgG	Servicebio	GB23303	1:3000
HRP-conjugated goat anti-mouse IgG	Servicebio	GB23301	1:5000

### Cell wound healing assay

A sterile pipette tip was used to create uniform scratches in a confluent monolayer of the K1, TT and 8305 C cells cultured in 24-well plates. The cells were then treated with Glu. Wound closure was monitored and images were captured at 0, 24, 48, and 78 h post-scratching using an inverted phase-contrast microscope.

### Cell proliferation analysis via CCK8 assay

The cells in good growth conditions were prepared as a cell suspension at a certain concentration, and 2 × 10^3^ cells/100 µL were added to each well of a 96-well cell culture plate. After the cells had adhered to the wall, 10 µL of CCK-8 solution was added, and the mixture was incubated in a 37 °C incubator for 0.5 h to 4 h. The absorbance was measured at a single wavelength of 450 nm.

### Immunohistochemistry and TUNEL staining were employed for comparative analysis

Tissues were embedded in paraffin and sectioned following standard procedures. Following immunohistochemistry, baking, dewaxing, hydration, antigen retrieval, and sealing procedures were performed. The reactive antibody corresponding to the target protein was subsequently incubated. After the addition of the reaction enhancer, the enzyme-enhanced goat anti-mouse/rabbit IgG polymer mixture was incubated, followed by DAB staining. Following hematoxylin retention, the samples were dehydrated and sealed. The positive rate was calculated through microscopic observation, with five fields examined. For TUNEL staining, each sample was prepared according to standard methods, equilibrated in 50 µL of equilibration buffer, and then incubated in 56 µL of TdT incubation buffer at 37 °C for 1 h. DAPI-induced nucleation was observed under a sealed microscope. Under a fluorescence microscope, apoptotic nuclei appeared red, whereas normal nuclei remained blue.

### ELISA of the secretion of serum metabolites

A glutamic acid enzyme-linked immunosorbent assay (ELISA) kit was used for detection and analysis. The test sample was a human serum sample. Standard and sample dilutions were added to blank wells, while human serum samples (100 µL/well) were added to other corresponding wells. The assay was performed according to the manufacturer’s instructions. After the procedure was complete, 100 µl/well of reaction stop solution was added, the mixture was mixed well, and the OD450 value was immediately measured (within 3 min).

### In vivo validation

TT cells were cultured, and OE-*LPAR1* vectors and control vectors (NCs) were constructed. Lentivirus was used to package TT anlotinib hydrochloride-resistant cell lines, and cells were collected at a concentration of 1 × 10^6^. Nude mice were divided into six groups to form subcutaneous xenografts: TT cell line, TT-anlotinib-resistant cell line, TT cell line + glu (100 mg/kg), TT-anlotinib-resistant cell line + glu(100 mg/kg), TT-anlotinib-resistant cell line + OE-*LPAR1*, and TT-anlotinib-resistant cell line + OE-*LPAR1* + glu (100 mg/kg) (*n* = 6). The tumor-bearing status, weight changes, behavioral changes, and response to external stimuli of the mice were observed. The mice were sacrificed at the appropriate time points, and the tumor tissues were removed. qPCR, IHC, and WB were used to detect the expression of key proteins. Eyeball blood was collected, and the glutamic acid content was detected via ELISA according to the manufacturer’s instructions. The experimental data were collected, and the results were statistically processed and analyzed.

### Statistical analysis

Independent student’s t tests were used for comparison between two groups, and ANOVA tests were used for comparison between multiple groups.Pearson correlation was used to analyze the correlation between two continuous variables. A *P* value < 0.05 indicated that the difference between the two groups was statistically significant. All calculations were performed using GraphPad Prism 5 and SPSS 15.0 software.

## Results

### Glutamate inhibits the expression of *LPAR1* and promotes the progression of malignant biological behavior in tumors

**Fig. 1:**
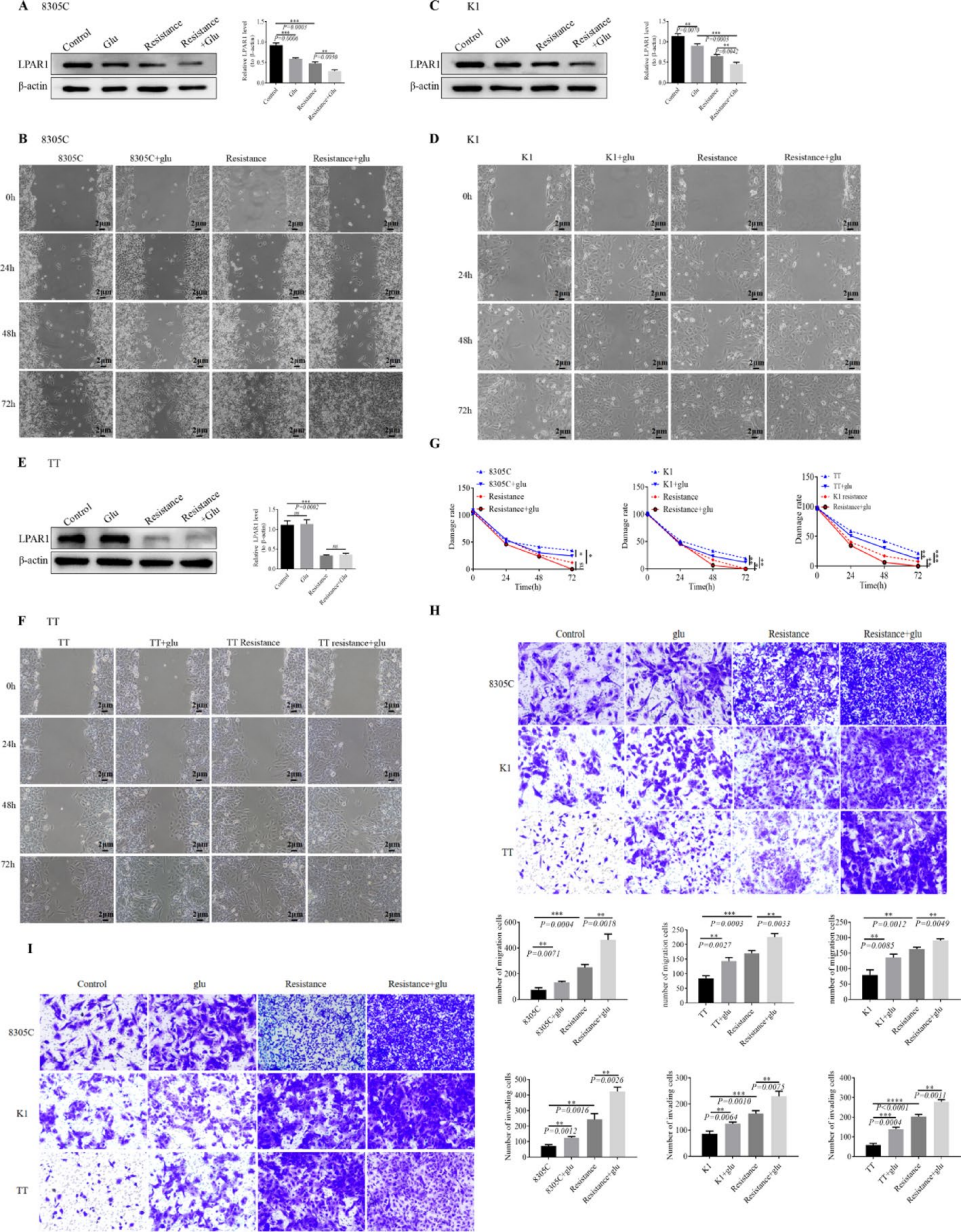
The potential regulatory relationship between glutamate and *LPAR1* was explored by detecting the expression level via WB, and the cell biological function was verified. **A** The expression of LPAR1 in each treatment group of 8305 C cells after glutamate intervention or no intervention was detected. **B** The scratch repair ability of 8305 C cells in each treatment group with or without glutamate was detected via a cell scratch assay. **C** The expression of LPAR1 in each treatment group of K1 cells after glutamate intervention or no intervention was detected. **D** The scratch repair ability of each treatment group of K1 cells with or without glutamate was detected via a cell scratch assay. **E** The expression of LPAR1 in TT cells from each treatment group was detected after glu intervention or no intervention. **F** The scratch repair ability of TT cells in each treatment group with or without glutamate was detected via a scratch assay. **G** Statistical graph of the scratch repair ability of 8305, K1 and TT cells in each treatment group. *P* ^*^ represents a P value < 0.05, which indicates a significant difference. **H** **I** Cell migration and invasion experiments

To investigate the impact of glutamate on *LPAR1*, we conducted glutamate intervention experiments. The results indicated that in both K1 and 8305 C cell lines, compared to the control group, the expression of *LPAR1* in the glu group were significantly decreased, and a similar trend were observed in the resistant group, but glu intervention did not significantly inhibit *LPAR1* expression in TT cell line.Meanwhile, experiments showed that glutamate significantly increased the wound healing of K1,8305 C and TT sensitive and resistant cell lines (Fig. 1A, B, C, D, E and F). At the same time, glutamate increased the invasion and migration of K1, 8305 C, and TT cell lines (Fig. 1G, H and I).

**Fig. 2:**
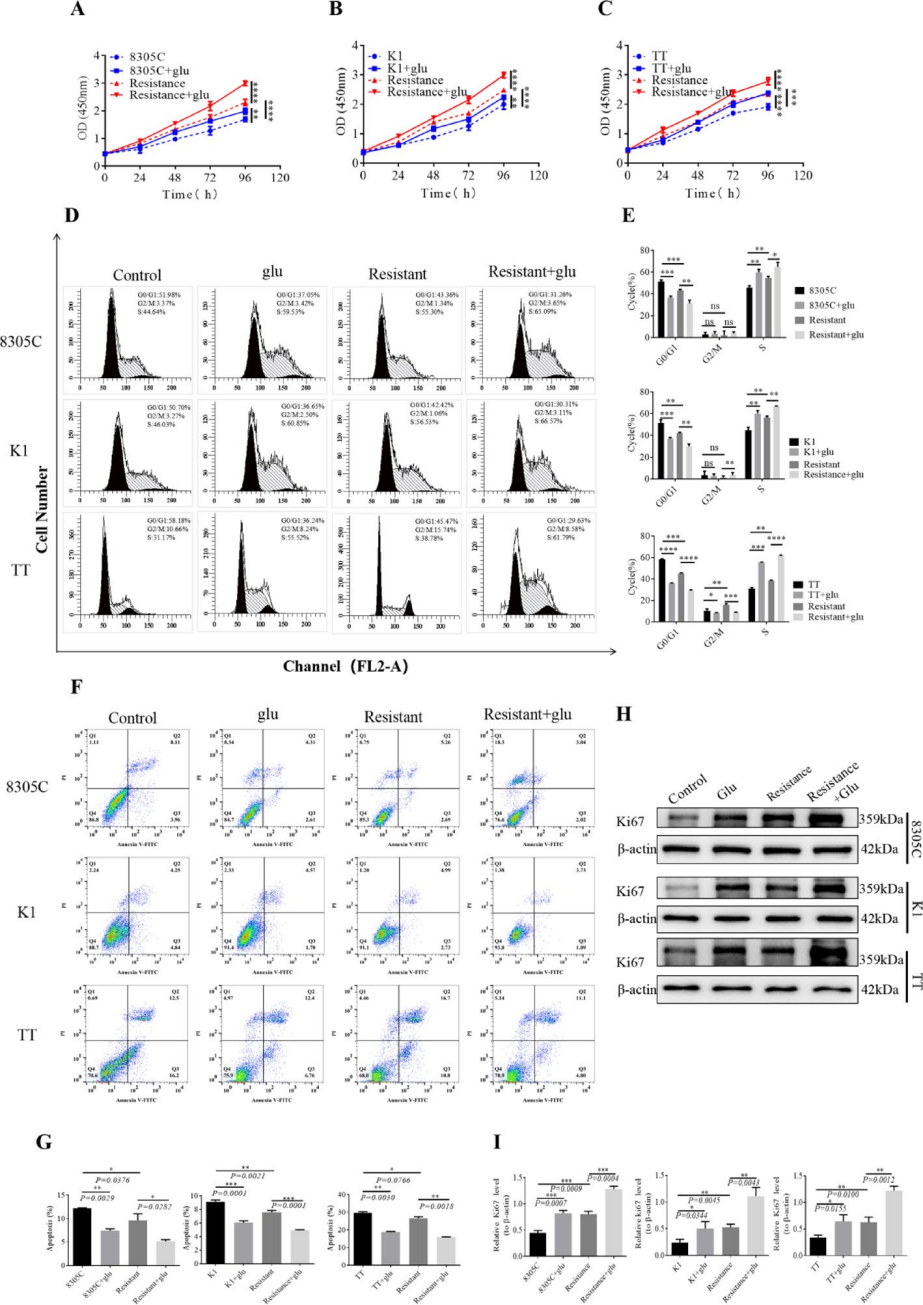
The potential role of glutamate in TC-anlotinib resistance was further investigated by detecting cell proliferation, cell cycle progression and apoptosis. A CCK8 kit was used to detect the effects of glutamate on cell proliferation in each 8305 C, K1 and TT treatment group: (**A**) 8305 C; **B** K1; **C** TT. The effects of glutamate on the cell cycle/apoptosis were detected via flow cytometry: **D** cell cycle; **F** cell apoptosis. The statistical graphs show the effect of glutamate on the cell cycle/apoptosis in each group. **E** Cell cycle; **G** cell apoptosis. **H** The Ki67 cell proliferation index of each group of cells with/without glutamate treatment was detected via WB. **I** The histogram shows the expression level of Ki67 in each group of cells, with a *P* value < 0.05 indicating a significant difference

Subsequent CCKB experiments and flow cytometry results indicated that glutamate significantly promoted cell proliferation (Fig. 2A, B and C) and significantly decreased the G0/G1 phase ratio and apoptosisin K1, 8305 C and TT sensitive and resistant cell lines (Fig. 2D, E, F and G). The Ki67 proliferation index results further revealed that glutamate significantly stimulated cell proliferation in both sensitive and resistant cell lines (Fig. 2H and I).

### The impact of glutamate on the MAPK pathway

**Fig. 3:**
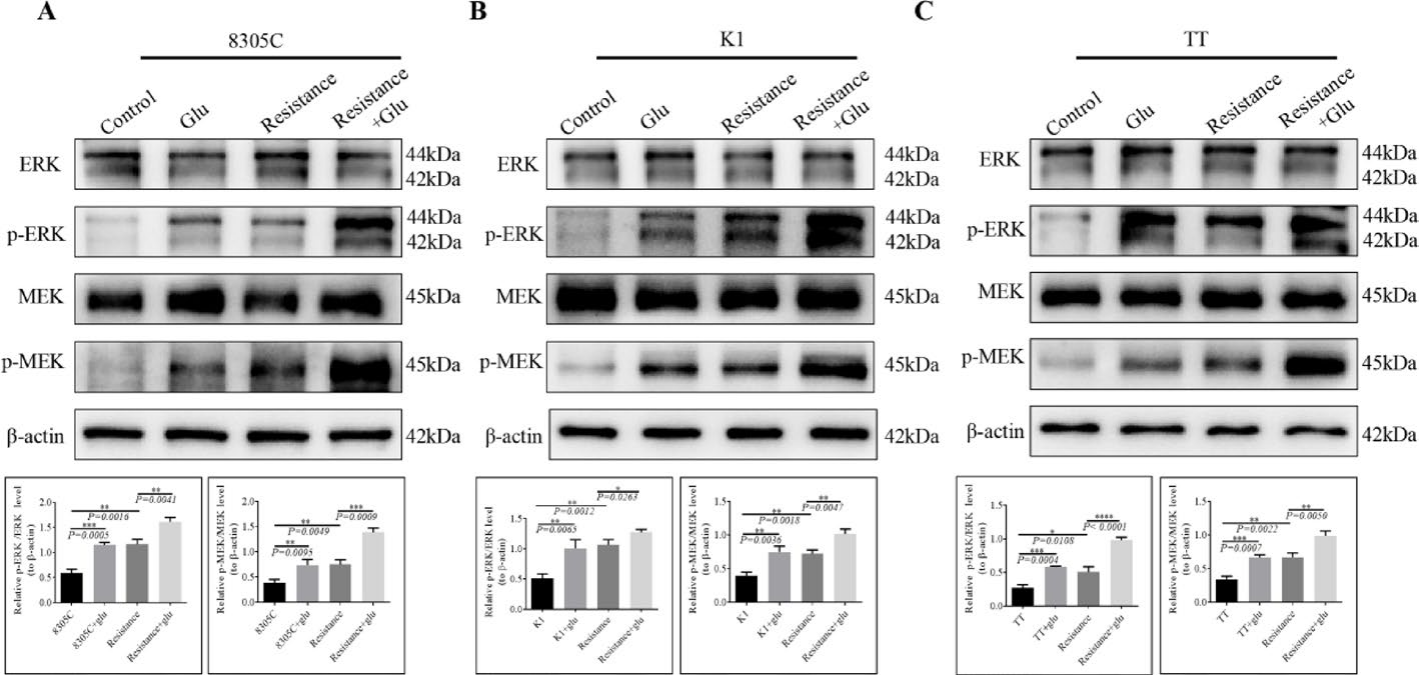
WB was used to detect changes in the expression of pathway proteins in each group of TC cells: **A** 8305 C; **B** K1; **C** TT. p-ERK is the phosphorylated ERK, p-MEK is the phosphorylated MEK, and β-actin is the internal reference. The histograms show the expression level of Ki67 in each group of cells, with a *P* value < 0.05 indicating a significant difference

The precise mechanism through which glutamate contributes to TC-anlotinib resistance remains unclear. The MAPK/ERK/MEK pathway has been demonstrated to be linked to chemotherapy resistance in numerous malignant tumors [14, 15], and the MAPK pathway has been reported to be involved in the growth and drug resistance of thyroid cancer [16–18]. Our study aimed to investigate the functional role of the MAPK pathway in TC-anlotinib resistance. Our findings revealed that glutamate significantly elevated the levels of p-ERK/ERK and p-MEK/MEK in both the TC-anlotinib-sensitive and TC-anlotinib-resistant strains, with broadly similar results across the three cell lines (Fig. 3A, B and C).

### Further analysis of the regulatory relationships among glutamate, *LPAR1*, and the MAPK/ERK/MEK signaling pathway

**Fig. 4:**
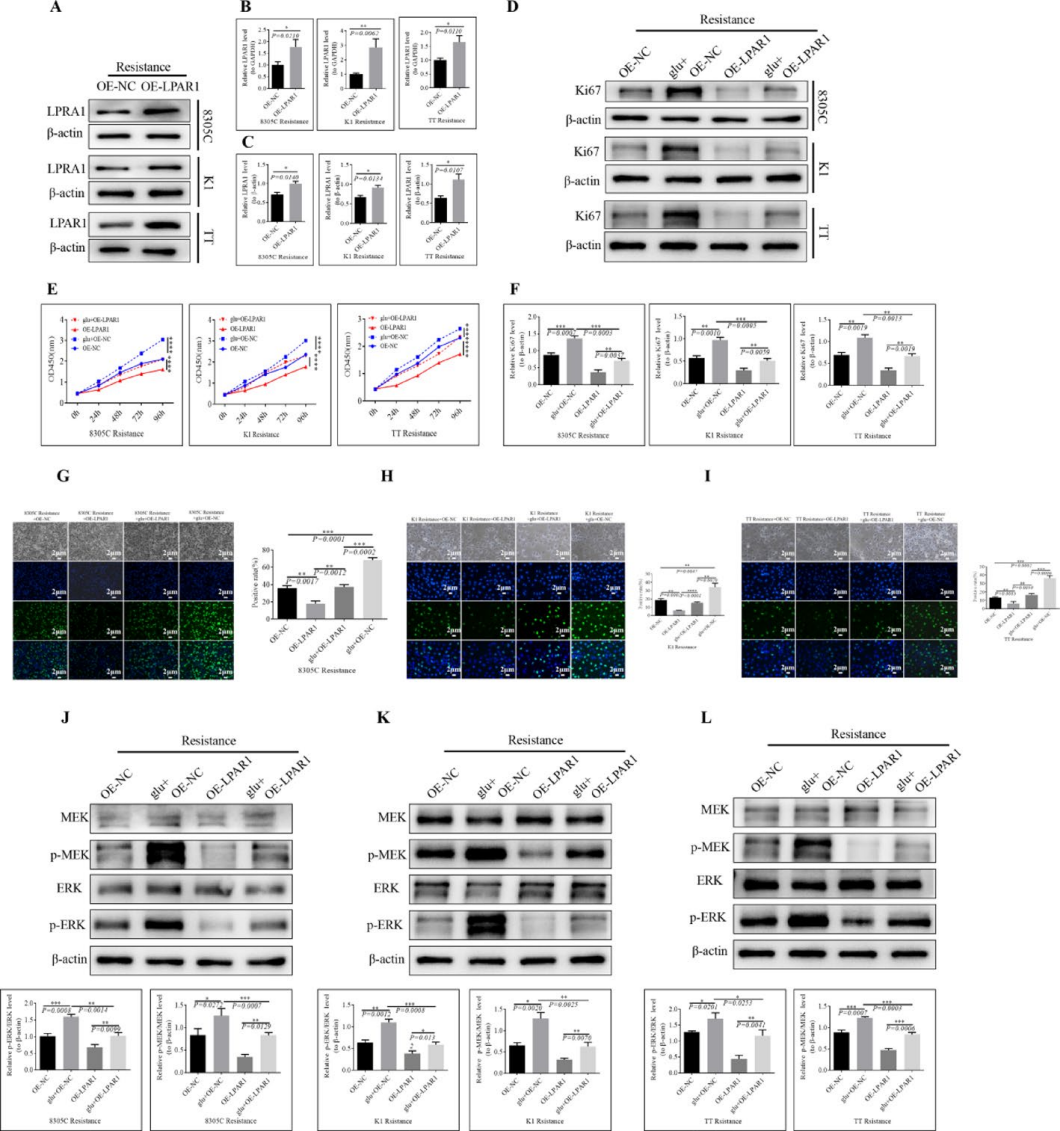
The OE*-LPAR1* lentivirus stable transmissible cell line was constructed to explore its potential regulatory mechanism in TC-anlotinib resistance. **A** The overexpression efficiency of *LPAR1* in each group was detected via WB, and β-actin was used as the internal control. **B** The overexpression efficiency of *LPAR1* in each group was determined via QPCR, and GAPDH was used as the internal control. **C** Corresponding statistical column chart of the data in Figure A. (**D**) Effects of OE*-LPAR1* and glutamate on the Ki67 proliferation index in TC-resistant cell lines. **E** Effects of OE*-LPAR1* and glutamate on the proliferation of each group of TC cells. **F** The histogram shows the expression level of Ki67 in each group of cells, with a *P* value < 0.05 indicating a significant difference. An EdU assay was also used to determine the effects of OE*-LPAR1* and glutamate on cell proliferation in each group: **G** 8305 C; **H** K1; **I** TT. The effects of OE*-LPAR1* and glutamate on MAPK signaling-related proteins in TC-resistant cell lines were tested via WB: **J** 8305 C; **K** K1; **L** TT. p-ERK is the phosphorylated ERK, p-MEK is the phosphorylated MEK, and β-actin is the internal reference. The bar graphs in the figure show the differences in each group of cells, with a *P* value < 0.05 indicating a significant difference

We next investigated whether glutamate truly activates the MAPK pathway by stably inhibiting the expression of *LPAR1*, thereby promoting resistance in TC cells to anlotinib. We used CCK8, EdU, and WB assays to assess the effects of OE*-LPAR1* and glutamate on cell proliferation. First, we validated the OE*-LPAR1* cell line (Fig. 4A, B, C and D). The results of the CCK8 assay demonstrated that OE*-LPAR1* significantly inhibited the proliferation ability of each group of cells. However, after combined intervention with OE*-LPAR1* and glutamate, the proliferative capacity of the cells was restored (Fig. 4E). The expression level of Ki67 was similar to that observed in the CCK8 experiment (Fig. 4F). The EdU results were consistent with those obtained through CCK8 and WB (Fig. 4G, H and I). We subsequently examined the expression of MAPK pathway-related proteins. The results indicated that OE*-LPAR1* significantly suppressed the expression levels of p-ERK/ERK and p-MEK/MEK in each group of cells. Nevertheless, under combined intervention with OE*-LPAR1* and glutamate, the expression levels of p-ERK/ERK and p-MEK/MEK increased (Fig. 4J, K and L).

**Fig. 5:**
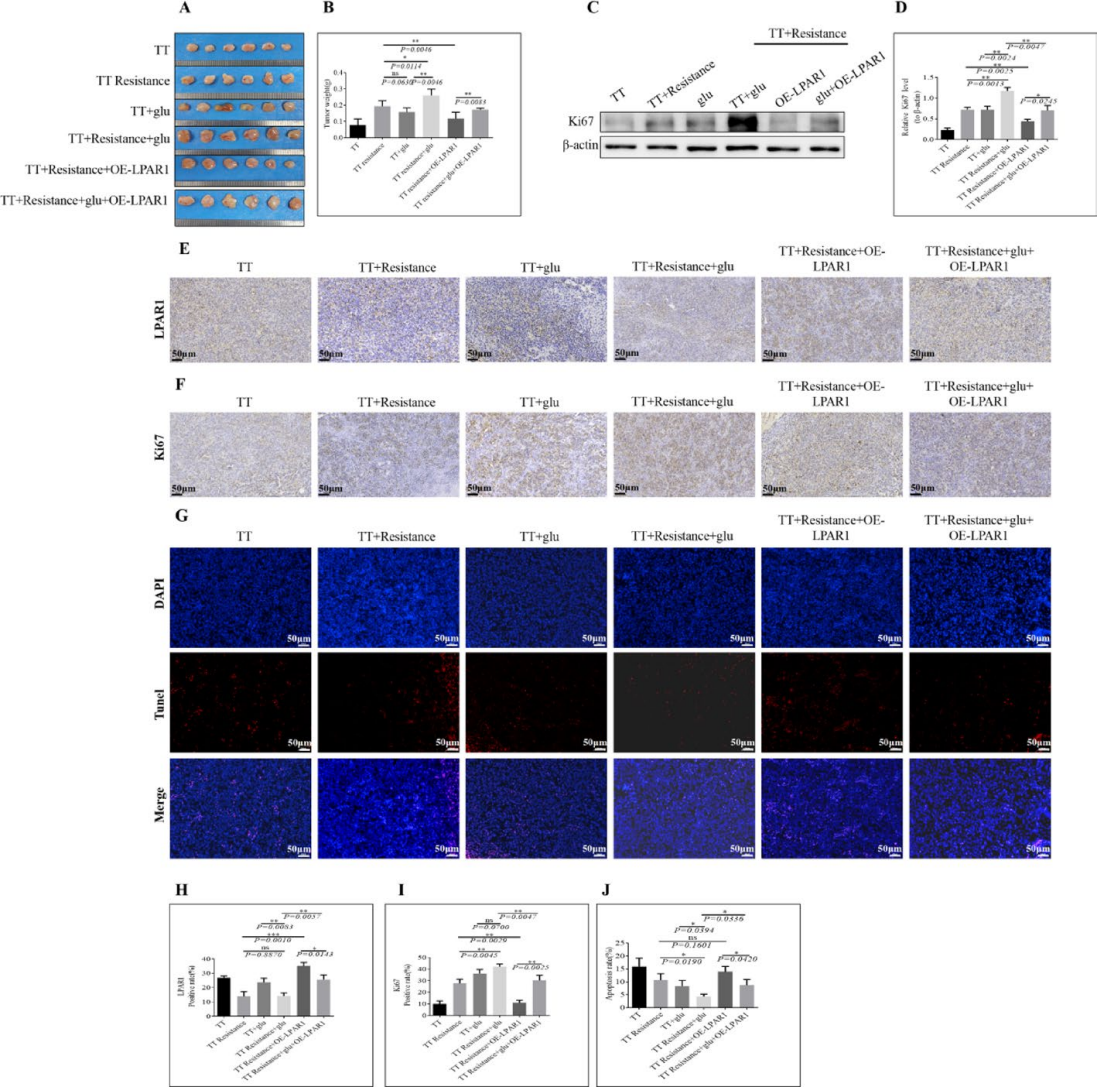
In vivo experiment. **A** A subcutaneous tumor formation model was constructed, and the tumor volume was observed and measured. **B** Statistical histogram of the data in Figure A; the horizontal coordinate represents the group, and the vertical coordinate represents the tumor volume. (**C**) Ki67 protein expression in each group was detected via WB, and β-actin was used as an internal reference. (**D**) Statistical histogram of the data in **C**; the horizontal coordinate represents the group, and the vertical coordinate represents the Ki67 expression level. The protein expression of LPAR1 and Ki67 was detected by IHC: **E** LPAR1; **F** Ki67. **G** TUNEL staining was used to detect apoptosis. **H** Statistical histogram of **E**; the horizontal coordinate is the group, and the vertical coordinate is the LPAR1 expression level. **I** Statistical histogram of the data in **F**; the horizontal coordinate represents the group, and the vertical coordinate represents the Ki67 expression level. **J** Statistical histogram of the data in **G**; the horizontal coordinate represents the group, and the vertical coordinate represents the degree of apoptosis. The bar graph shows the differences in each group of cells, with a *P* value < 0.05 indicating a significant difference

For in vivo experiments, a subcutaneous tumor formation model in nude mice was established by subcutaneously injecting cells (5 × 10^6^ cells/mL) into each group. Statistical analysis revealed that the tumor volume followed the order of TT + Resistance + glu > TT + Resistance > TT + Resistance + glu + OE-*LPAR1* > TT + glu > TT + Resistance + OE-*LPAR1* > TT (Fig. 5A and B). WB data demonstrated that the protein expression of Ki67 in the TT + Resistance + glu group was significantly greater than that in the TT + Resistance group. Compared with that in the TT + glu group, Ki67 expression in the TT + Resistance + glu group was notably elevated(Fig. 5C and D). Immunohistochemistry was used to detect LPAR1 expression in tumor tissues. Compared with that in the TT + Resistance group, LPAR1 expression was not significantly different in the TT + Resistance + glu group; however, compared with that in the TT + glu group, LPAR1 expression was significantly lower in the TT + Resistance + glu group. Furthermore, compared with that in the TT + Resistance + glu + OE*-LPAR1* group, LPAR1 expression was significantly greater in the TT + Resistance + OE-*LPAR1* group. However, compared with that in the TT + Resistance + glu + OE-*LPAR1* group, Ki67 expression in the TT + Resistance + OE-*LPAR1* group was significantly lower. The IHC results aligned with the WB findings (Fig. 5E and F). TUNEL staining revealed that the level of apoptosis in the TT + Resistance + glu group was notably lower than that in the TT + Resistance group and lower than that in the TT + glu group. In contrast, compared with that in the TT + Resistance + glu + OE-*LPAR1* group, the level of apoptosis in the TT + Resistance + OE*-LPAR1* group was significantly greater (Fig. 5G). This trend aligned with the expression level of LPAR1 but was opposite to that of Ki67. Figure 5H and I, and 5J show the statistical histograms of Fig. 5E and F, and 5G, respectively.

**Fig. 6:**
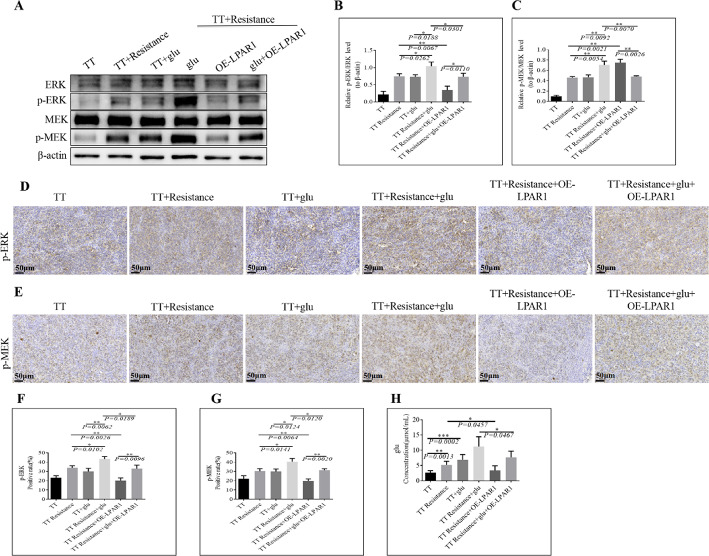
The key proteins in the MAPK signaling axis were further detected. **A** MAPK pathway protein expression in tumor tissues was detected via WB, β-actin was used as the internal reference, and the gray value was used to quantify the protein expression level. **B** and **C** Bar graphs showing the differences in protein expression in each group of cells. P values < 0.05 indicate significant differences:(**B** p-ERK; **C** p-MEK. IHC was used to observe the expression of the MAPK signaling pathway-related proteins **D** p-ERK and **E** p-MEK. The histogram shows the differences in p-ERK and p-MEK expression levels between Figures **D** and **E**, with P values < 0.05 indicating a significant difference: **F**) p-ERK. **G** p-MEK. **H** The serum content of glutamate in each group was detected by ELISA; the horizontal coordinate represents the group, and the vertical coordinate reflects the serum glutamate content

In the subsequent study, WB and IHC analyses revealed that the protein expression levels of p-ERK/ERK and p-MEK/MEK were notably elevated in the TT + Resistance + glu group compared with those in the TT + Resistance group. Compared with those in the TT + glu group, the p-ERK/ERK and p-MEK/MEK proteins were significantly increased in the TT + Resistance + glu group. However, compared with that in the TT + Resistance + glu + OE*-LPAR1* group, the expression of p-ERK/ERK and p-MEK/MEK was significantly lower in the TT + Resistance + OE-*LPAR1* group (Fig. 6A, B and C). The IHC results were consistent with the WB results (Fig. 6D, E, F and G). Finally, we reexamined the changes in glutamate levels in the peripheral blood of the mice across all the groups. Compared with that in the TT + Resistance group, the glutamate content was significantly lower in the TT + Resistance + OE*-LPAR1* group (Fig. 6H).

## Discussion

We observed an abnormal increase in glutamate levels in TC-anlotinib-resistant cell lines during early-stage metabolome sequencing, accompanied by an abnormal decrease in *LPAR1* levels, as revealed by transcriptome sequencing, both of which were subsequently confirmed in tumor tissues. To delve deeper into the impact of the differentially abundant metabolite glutamate on TC-anlotinib resistance, we conducted glutamate intervention experiments. The findings revealed that glutamate notably enhanced the wound healing, invasion, and migration capabilities of both sensitive and resistant strains, stimulating cell proliferation and significantly decreasing the rate of apoptosis. Additionally, we assessed the effect of glutamate on *LPAR1* expression. The results indicated that glutamate significantly suppressed *LPAR1* expression in the K1 and 8305 C cell lines. However, the trend of *LPAR1* expression inhibition in TT-resistant strains after glutamate intervention remains unclear, and its regulatory relationship deserves further study. Glutamate metabolism has emerged as an appealing target for cancer therapy [19], as it regulates multiple biological processes, including nutrition, metabolism, signal transduction, and drug resistance [20]. A deeper understanding of the mechanisms by which glutamate drives tumor progression and drug resistance can offer a theoretical foundation for targeted tumor therapy. Research has illuminated the importance of the glutamate metabolic pathway in chemotherapy resistance, tumor growth, and metabolism [21]. Previous studies have shown that glutamate leads to neuronal death in glioblastoma, thus promoting tumor growth and participating in seizures and the possibility of malignant transformation in the early stage of pancreatic cancer [22].Nevertheless, the factors driving glutamate-mediated resistance to TC-anlotinib remain unreported. Our findings indicated that the biological environment fostered by glutamate enhanced the malignant transformation potential of TC cells and significantly suppressed the expression of *LPAR1*.

Lysophosphatidic acid receptor 1 *(LPAR1*), a 41 kDa protein composed of 364 amino acids [23]. *LPAR1* is widely expressed, and it mainly plays its role through activating MAPK, PI3K/Akt, Rho pathway, etc [24]. According to the literature, *LPAR1* is closely related to ovarian cancer, lung cancer, breast cancer, etc [25], but there is no report on thyroid tumors.Numerous previous studies have demonstrated that *LPAR1-6* play pivotal roles in numerous core processes of tumorigenesis and progression via the MAPK pathway, including proliferation, survival, migration, invasion, metastasis, cancer stem cells, the tumor microenvironment, and therapeutic resistance [26, 27]. Consequently, the MAPK pathway may also serve as a downstream substrate of *LPAR1*. Furthermore, the MAPK pathway represents a ubiquitous signal transduction pathway that regulates various aspects of life and frequently undergoes alterations in diseases, including its role in modulating cancer drug sensitivity and drug resistance [28]. Therefore, further investigations into whether the MAPK pathway truly represents a downstream substrate of *LPAR1* and whether the regulation of the MAPK pathway by LPAR1 contributes to the promotion of resistance to anlotinib in thyroid cancer are imperative.

In recent years, significant advancements have been made in cancer treatment and drug development to target the MAPK-MEK-ERK pathway. Extensive research on this pathway, along with the clarification of its sensitivity and resistance mechanisms, has yielded promising results [29–31]. Recent studies indicate that drug resistance often arises from reactivation of the MAPK/ERK pathway or the activation of other kinase signaling pathways [17, 32–34]. Through a comprehensive analysis utilizing transcriptomics and bioinformatics, coupled with a literature review, we observed that MAPK pathway proteins are closely related to *LPAR1* [13, 35, 36]. While our primary focus was on the role of *LPAR1* as a glutamate metabolic regulator in TC-anlotinib resistance, it is also possible that the MAPK pathway contributes to resistance, given its established association with chemotherapy resistance in various malignant tumors.

Notably, during our validation of glutamate function, the introduction of glutamate led to alterations in the expression of *LPAR1* and MAPK pathway proteins, suggesting a distinct regulatory relationship. Our findings also indicate that the overexpression of *LPAR1* significantly suppresses the expression and phosphorylation of MEK/ERK proteins, an effect that can be reversed by glutamate intervention. Elucidating this regulatory relationship is crucial for comprehending and analyzing the emergence of TC-anlotinib resistance. Consequently, we conducted in vivo experiments, and the results once again demonstrated that glutamate promoted the proliferation of TC cells and TC-anlotinib-resistant cells while also inhibiting *LPAR1* expression. *LPAR1* overexpression notably suppressed Ki67 expression and promoted apoptosis, whereas glutamate had the opposite effect. Moreover, LPAR1 overexpression significantly inhibited the expression of MAPK pathway-related p-ERK/ERK and p-MEK/MEK proteins, whereas glutamate intervention notably increased their expression. These data further support that the mechanism by which glutamate regulates TC-anlotinib resistance relies on its regulatory axis with *LPAR1* and the activation of the MAPK pathway. This is the first prospective study on the mechanism of TC-anlotinib resistance, revealing potential new therapeutic targets and promising prospects for treating anlotinib resistance in TC.

## Conclusion

**Fig. 7:**
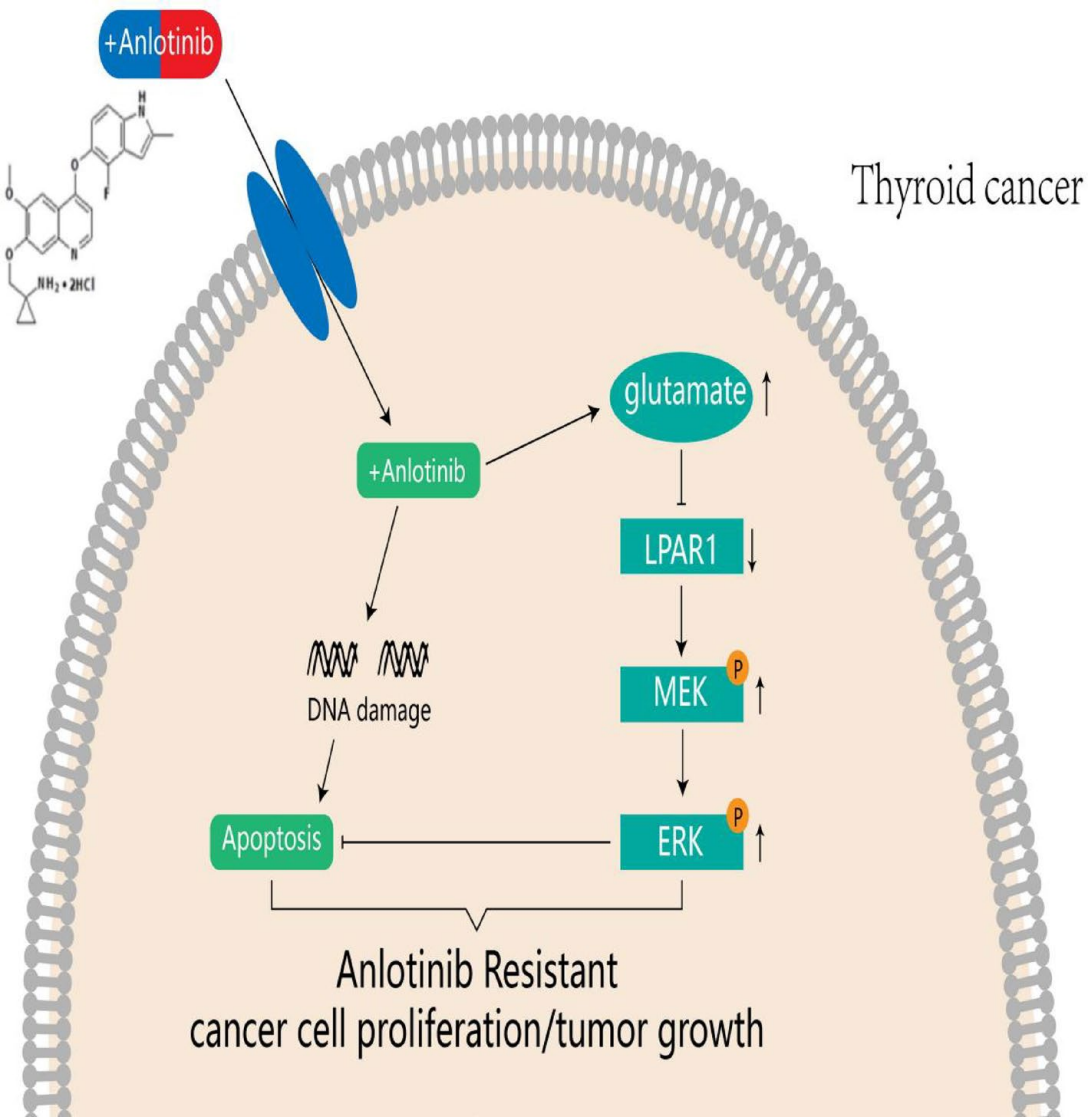
The potential regulatory mechanisms of glu and *LPAR1* in TC-Anlotinib resistance

Glutamate is one of the main metabolic substrates in the tumor microenvironment. It plays a pivotal role in drug resistance, engaging in complex interactions with tumor microenvironment components. The findings of this study indicate that alterations in glutamate metabolism can shield TC cells from anlotinib-induced apoptosis. Additionally, glutamate can suppress the expression of *LPAR1* and activate the MAPK signaling pathway. On the basis of these experimental results, we hypothesize that glutamate promotes resistance to anlotinib in TC by inhibiting *LPAR1* expression and activating the MAPK signaling pathway(Fig. 7). However, our results are solely derived from cell and animal experiments and lack further validation in tumors from human TC patients resistant to anlotinib. These limitations will be the focus of our future research.

In thyroid cancer, during Anlotinib treatment, it can trigger intracellular changes. Among them, the level of glutamate (Glu) is affected and activates LPAR1, which in turn phosphorylates and activates the MEK and ERK signaling pathways. The activation of this pathway inhibits the apoptosis process induced by Anlotinib, allowing cancer cells to evade apoptosis and ultimately leading to the proliferation of Anlotinib-resistant cancer cells and tumor growth. Glu and LPAR1 play a key potential regulatory role in TC - Anlotinib resistance through the regulation of the downstream MEK - ERK signaling pathway.

## Electronic supplementary material

Below is the link to the electronic supplementary material.

Supplementary Material 1

Supplementary Material 2

Supplementary Material 3

## Data Availability

All data involved in this study were obtained based on rigorous experimental design and implementation, ensuring the authenticity and reliability of the data. If researchers or institutions require access to the data from this study, please contact our corresponding author. We will provide necessary data support and sharing services according to specific conditions, while adhering to relevant regulations and policies.

## References

[1] Kotwal A, Fingeret A, Knape A, Patel A, Bradford Bell E, Goldner W. Thyroid Cancer survivorship: challenges and opportunities. Endocr Pract. 2024;30(11):1097–102. 10.1016/j.eprac.2024.08.003. Epub 2024 Aug 28. PMID: 39209023. 39209023 10.1016/j.eprac.2024.08.003

[2] Lim H, Devesa SS, Sosa JA, Check D, Kitahara CM. Trends in thyroid Cancer incidence and mortality in the united states, 1974–2013. JAMA. 2017;317(13):1338–48. 10.1001/jama.2017.2719. PMID: 28362912; PMCID: PMC8216772. 28362912 10.1001/jama.2017.2719 PMC8216772

[3] Fagin JA, Wells SA Jr. Biologic and clinical perspectives on thyroid Cancer. N Engl J Med. 2016;375(11):1054–67. 10.1056/NEJMra1501993. PMID: 27626519; PMCID: PMC5512163. 27626519 10.1056/NEJMra1501993 PMC5512163

[4] Silaghi H, Lozovanu V, Georgescu CE, Pop C, Nasui BA, Cătoi AF, Silaghi CA. State of the Art in the current management and future directions of targeted therapy for differentiated thyroid Cancer. Int J Mol Sci. 2022;23(7):3470. 10.3390/ijms23073470. PMID: 35408830; PMCID: PMC8998761. 35408830 10.3390/ijms23073470 PMC8998761

[5] Li S, Wang H. Research progress on mechanism and management of adverse drug reactions of anlotinib. Drug Des Devel Ther. 2023;17:3429–37. 10.2147/DDDT.S426898. PMID: 38024530; PMCID: PMC10657757. 38024530 10.2147/DDDT.S426898 PMC10657757

[6] Gao Y, Liu P, Shi R. Anlotinib as a molecular targeted therapy for tumors. Oncol Lett. 2020;20(2):1001–14. 10.3892/ol.2020.11685. Epub 2020 May 28. PMID: 32724339; PMCID: PMC7377159. 32724339 10.3892/ol.2020.11685 PMC7377159

[7] Huang NS, Wei WJ, Xiang J, Chen JY, Guan Q, Lu ZW, Ma B, Sun GH, Wang YL, Ji QH, Wang Y. The efficacy and safety of anlotinib in neoadjuvant treatment of locally advanced thyroid cancer: A Single-Arm phase II clinical trial. Thyroid. 2021;31(12):1808–13. 10.1089/thy.2021.0307. Epub 2021 Nov 29. PMID: 34610756. 34610756 10.1089/thy.2021.0307

[8] Li D, Chi Y, Chen X, Ge M, Zhang Y, Guo Z, Wang J, Chen J, Zhang J, Cheng Y, Li Z, Liu H, Qin J, Zhu J, Cheng R, Xu Z, Zheng X, Tang P, Gao M. Anlotinib in locally advanced or metastatic medullary thyroid carcinoma: A randomized, Double-Blind phase IIB trial. Clin Cancer Res. 2021;27(13):3567–75. 10.1158/1078-0432.CCR-20-2950. Epub 2021 Apr 8. PMID: 33832949. 33832949 10.1158/1078-0432.CCR-20-2950

[9] Zhang Y, Xing Z, Liu T, Tang M, Mi L, Zhu J, Wu W, Wei T. Targeted therapy and drug resistance in thyroid cancer. Eur J Med Chem. 2022;238:114500. 10.1016/j.ejmech.2022.114500. Epub 2022 May 29. PMID: 35675754. 35675754 10.1016/j.ejmech.2022.114500

[10] Sun Y, Du F, Gao M, et al. Anlotinib for the treatment of patients with locally advanced or metastatic medullary thyroid Cancer. THYROID. 2018;28(11):1455–61. 10.1089/thy.2018.0022. 30142994 10.1089/thy.2018.0022

[11] Liu B, Peng Y, Su Y, Diao C, Qian J, Zhan X, Cheng R. Transcriptome and metabolome sequencing identifies glutamate and LPAR1 as potential factors of anlotinib resistance in thyroid cancer. Anticancer Drugs. 2024;35(8):741–51. Epub 2024 May 31. PMID: 38820067. 38820067 10.1097/CAD.0000000000001626

[12] Kajitani N, Okada-Tsuchioka M, Inoue A, Miyano K, Masuda T, Boku S, Iwamoto K, Ohtsuki S, Uezono Y, Aoki J, Takebayashi M. G protein-biased LPAR1 agonism of prototypic antidepressants: implication in the identification of novel therapeutic target for depression. Neuropsychopharmacology. 2024;49(3):561–72. 10.1038/s41386-023-01727-9. Epub 2023 Sep 6. PMID: 37673966; PMCID: PMC10789764. 37673966 10.1038/s41386-023-01727-9 PMC10789764

[13] Plastira I, Bernhart E, Joshi L, Koyani CN, Strohmaier H, Reicher H, Malle E, Sattler W. MAPK signaling determines lysophosphatidic acid (LPA)-induced inflammation in microglia. J Neuroinflammation. 2020;17(1):127. 10.1186/s12974-020-01809-1. PMID: 32326963; PMCID: PMC7178949. 32326963 10.1186/s12974-020-01809-1 PMC7178949

[14] Feng L, Wang C, Zhang C, Zhang W, Zhu W, He Y, Xia Z, Song W. p38 MAPK inhibitor SB202190 suppresses ferroptosis in the glutamate-induced retinal excitotoxicity glaucoma model. Neural Regen Res. 2024;19(10):2299–309. 10.4103/1673-5374.391193. Epub 2023 Dec 21. PMID: 38488564; PMCID: PMC11034608. 38488564 10.4103/1673-5374.391193 PMC11034608

[15] Gazzeri S, Zubchuk N, Montaudon E, Nemati F, Huot-Marchand S, Berardi G, Pucciarelli A, Dib Y, Nerini D, Oddou C, Pezet M, David-Boudet L, Ardin C, de Fraipont F, Maraver A, Girard N, Decaudin D, Toffart AC, Eymin B. PPP3CB overexpression mediates EGFR TKI resistance in lung tumors via calcineurin/mek/erk signaling. Life Sci Alliance. 2024;7(12):e202402873. 10.26508/lsa.202402873. PMID: 39353739; PMCID: PMC11447527. 39353739 10.26508/lsa.202402873 PMC11447527

[16] Yan M, Luo X, Han H, Qiu J, Ye Q, Zhang L, Wang Y. ROCK2 increases drug resistance in acute myeloid leukemia via metabolic reprogramming and MAPK/PI3K/AKT signaling. Int Immunopharmacol. 2024;140:112897. 10.1016/j.intimp.2024.112897. Epub 2024 Aug 9. PMID: 39126734. 39126734 10.1016/j.intimp.2024.112897

[17] Schubert L, Mariko ML, Clerc J, Huillard O, Groussin L. MAPK pathway inhibitors in thyroid cancer: preclinical and clinical data. Cancers (Basel). 2023;15(3):710. 10.3390/cancers15030710. PMID: 36765665; PMCID: PMC9913385. 36765665 10.3390/cancers15030710 PMC9913385

[18] Zeng J, Zhang L, Huang L, Yu X, Han L, Zheng Y, Wang T, Zhang N, Yang M. MAZ promotes thyroid cancer progression by driving transcriptional reprogram and enhancing ERK1/2 activation. Cancer Lett. 2024;602:217201. 10.1016/j.canlet.2024.217201. Epub 2024 Aug 27. PMID: 39197582. 39197582 10.1016/j.canlet.2024.217201

[19] Kimura T, Doolittle WKL, Kruhlak M, Zhao L, Hwang E, Zhu X, Tang B, Wolcott KM, Cheng SY. Inhibition of MEK signaling attenuates Cancer stem cell activity in anaplastic thyroid Cancer. Thyroid. 2024;34(4):484–95. 10.1089/thy.2023.0521. Epub 2024 Jan 16. PMID: 38115586; PMCID: PMC10998707. 38115586 10.1089/thy.2023.0521 PMC10998707

[20] Koda S, Hu J, Ju X, Sun G, Shao S, Tang RX, Zheng KY, Yan J. The role of glutamate receptors in the regulation of the tumor microenvironment. Front Immunol. 2023;14:1123841. 10.3389/fimmu.2023.1123841. PMID: 36817470; PMCID: PMC9929049. 36817470 10.3389/fimmu.2023.1123841 PMC9929049

[21] Brosnan JT, Brosnan ME. Glutamate: a truly functional amino acid. Amino Acids. 2013;45(3):413-8. 10.1007/s00726-012-1280-4. Epub 2012 Apr 18. PMID: 22526238. 10.1007/s00726-012-1280-4 22526238

[22] Cui Q, Wang JQ, Assaraf YG, Ren L, Gupta P, Wei L, Ashby CR Jr, Yang DH, Chen ZS. Modulating ROS to overcome multidrug resistance in cancer. Drug Resist Updat. 2018;41:1–25. Epub 2018 Nov 14. PMID: 30471641. 30471641 10.1016/j.drup.2018.11.001

[23] Stepulak A, Rola R, Polberg K, Ikonomidou C. Glutamate and its receptors in cancer. J Neural Transm (Vienna). 2014;121(8):933 – 44. doi: 10.1007/s00702-014-1182-6. Epub 2014 Mar 9. PMID: 24610491; PMCID: PMC4133641. 10.1007/s00702-014-1182-6 PMC4133641 24610491

[24] van Meeteren LA, Moolenaar WH. Regulation and biological activities of the autotaxin-LPA axis. Prog Lipid Res. 2007;46(2):145 – 60. 10.1016/j.plipres.2007.02.001. Epub 2007 Mar 16. PMID: 17459484. 10.1016/j.plipres.2007.02.001 17459484

[25] Ishii I, Fukushima N, Ye X, Chun J. Lysophospholipid receptors: signaling and biology. ANNU REV BIOCHEM. 2004-01-01; 73 321 – 54. 10.1146/annurev.biochem.73.011303.073731. PMID: 15189145. 10.1146/annurev.biochem.73.011303.073731 15189145

[26] Xiang H, Lu Y, Shao M, Wu T. Lysophosphatidic acid receptors: biochemical and clinical implications in different diseases. J Cancer. 2020;11(12):3519–35. 10.7150/jca.41841. PMID: 32284748; PMCID: PMC7150451. 32284748 10.7150/jca.41841 PMC7150451

[27] Quan M, Cui JJ, Feng X, Huang Q. The critical role and potential target of the autotaxin/lysophosphatidate axis in pancreatic cancer. Tumour Biol. 2017;39(3):1010428317694544. 10.1177/1010428317694544. PMID: 28347252. 10.1177/1010428317694544 28347252

[28] Lin YH, Lin YC, Chen CC. Lysophosphatidic acid receptor antagonists and cancer: the current trends, clinical implications, and trials. Cells. 2021;10(7):1629. 10.3390/cells10071629. PMID: 34209775; PMCID: PMC8306951. 34209775 10.3390/cells10071629 PMC8306951

[29] Lee S, Rauch J, Kolch W. Targeting MAPK signaling in cancer: mechanisms of drug resistance and sensitivity. Int J Mol Sci. 2020;21(3):1102. 10.3390/ijms21031102. PMID: 32046099; PMCID: PMC7037308. 32046099 10.3390/ijms21031102 PMC7037308

[30] Pashirzad M, Khorasanian R, Fard MM, Arjmand MH, Langari H, Khazaei M, Soleimanpour S, Rezayi M, Ferns GA, Hassanian SM, Avan A. The Therapeutic Potential of MAPK/ERK Inhibitors in the Treatment of Colorectal Cancer. Curr Cancer Drug Targets. 2021;21(11):932–943. 10.2174/1568009621666211103113339. PMID: 34732116. 10.2174/1568009621666211103113339 34732116

[31] Pandian J, Ganesan K. Delineation of gastric tumors with activated ERK/MAPK signaling cascades for the development of targeted therapeutics. Exp Cell Res. 2022;410(1):112956. 10.1016/j.yexcr.2021.112956. Epub 2021 Dec 2. PMID: 34864005. 34864005 10.1016/j.yexcr.2021.112956

[32] Chen N, Fang W, Lin Z, Peng P, Wang J, Zhan J, Hong S, Huang J, Liu L, Sheng J, Zhou T, Chen Y, Zhang H, Zhang L. KRAS mutation-induced upregulation of PD-L1 mediates immune escape in human lung adenocarcinoma. Cancer Immunol Immunother. 2017;66(9):1175–87. Epub 2017 Apr 27. PMID: 28451792; PMCID: PMC5579171. 28451792 10.1007/s00262-017-2005-z PMC5579171

[33] Scerri J, Scerri C, Schäfer-Ruoff F, Fink S, Templin M, Grech G. PKC-mediated phosphorylation and activation of the MEK/ERK pathway as a mechanism of acquired trastuzumab resistance in HER2-positive breast cancer. Front Endocrinol (Lausanne). 2022;13:1010092. 10.3389/fendo.2022.1010092. PMID: 36329884; PMCID: PMC9623415. 36329884 10.3389/fendo.2022.1010092 PMC9623415

[34] Rebecca V, Jagirdar K, Portuallo M, Wei M, Wilhide M, Bravo J, Robertson B, Alicea G, Aguh C, Xiao M, Godok T, Fingerman D, Brown G, Herlyn M, Guo B, Toska E, Zabransky D, Wubbenhorst B, Nathanson K, Kwatra S, Goyal Y, Ji H, Liu Q. ERK Hyperactivation Serves as a Unified Mechanism of Escape in Intrinsic and Acquired CDK4/6 Inhibitor Resistance in Acral Lentiginous Melanoma. Res Sq [Preprint]. 2023 Apr 20:rs.3.rs-2817876. 10.21203/rs.3.rs-2817876/v1. Update in: Oncogene. 2024;43(6):395–405. doi: 10.1038/s41388-023-02900-6. PMID: 37131684; PMCID: PMC10153386. 10.1038/s41388-023-02900-6 PMC10837073 38066089

[35] Ma W, Tian M, Hu L, Ruan X, Zhang W, Zheng X, Gao M. Early combined SHP2 targeting reverses the therapeutic resistance of Vemurafenib in thyroid Cancer. J Cancer. 2023;14(9):1592–604. 10.7150/jca.83853. PMID: 37325052; PMCID: PMC10266257. 37325052 10.7150/jca.83853 PMC10266257

[36] Lee GH, Cheon J, Kim D, Jun HS. Lysophosphatidic acid promotes epithelial-Mesenchymal transition in kidney epithelial cells via the LPAR1/MAPK-AKT/KLF5 signaling pathway in diabetic nephropathy. Int J Mol Sci. 2022;23(18):10497. 10.3390/ijms231810497. PMID: 36142408; PMCID: PMC9500642. 36142408 10.3390/ijms231810497 PMC9500642

