# Supplementary material for: Glutamate activates the MAPK pathway by inhibiting LPAR1 expression and promotes anlotinib resistance in thyroid cancer
Source: Discov Oncol. 2025 Jun 13;16:1082. doi: 10.1007/s12672-025-02853-0 (PMC12165934; doi:10.1007/s12672-025-02853-0)
Supplement: Supplementary file 1 — Supplementary Material 1 [file 12672_2025_2853_MOESM1_ESM.pptx]

## Slide 1
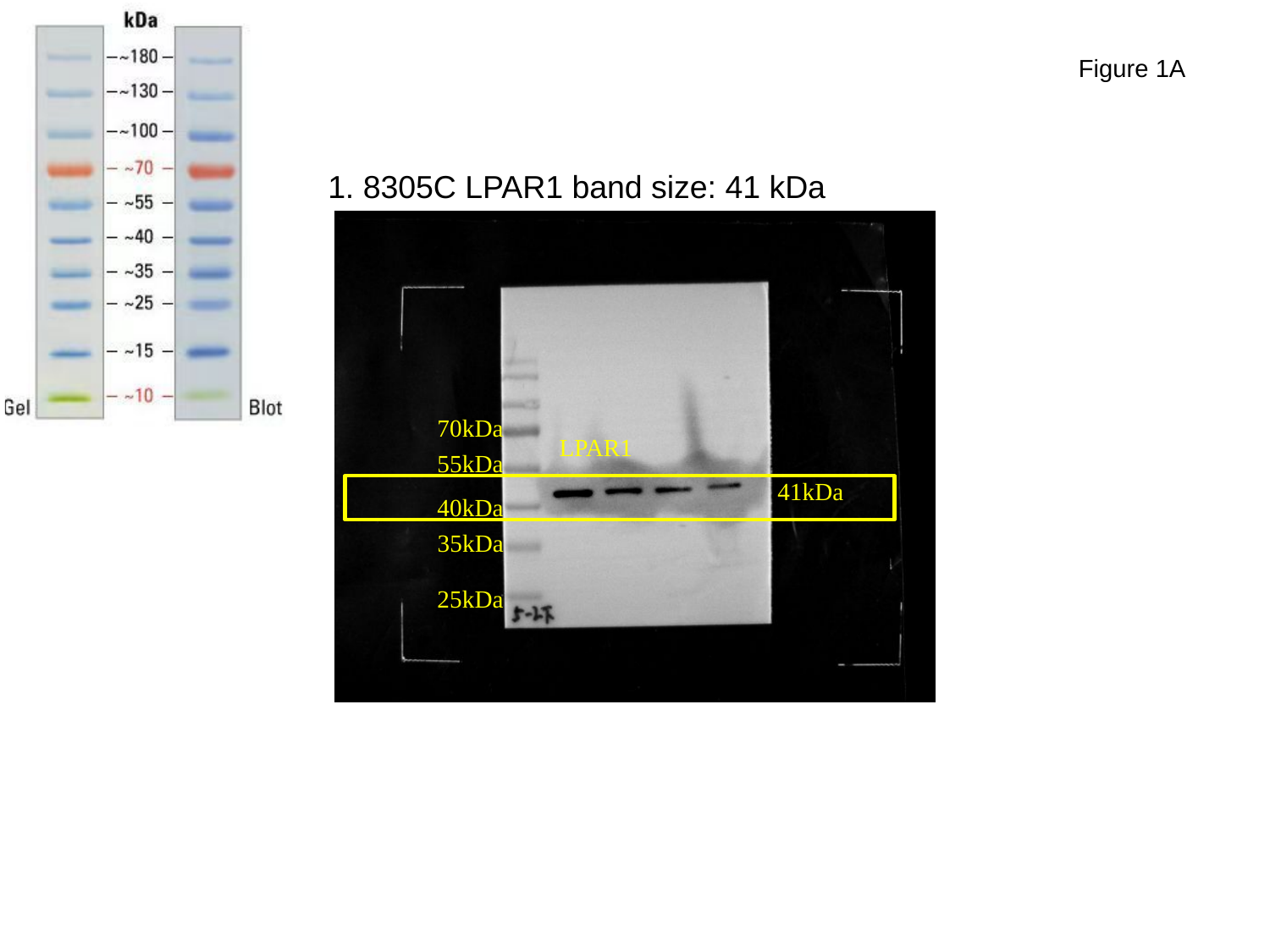

Figure 1A
1. 8305C LPAR1 band size: 41 kDa
70kDa
LPAR1
55kDa
41kDa
40kDa
35kDa
25kDa

## Slide 2
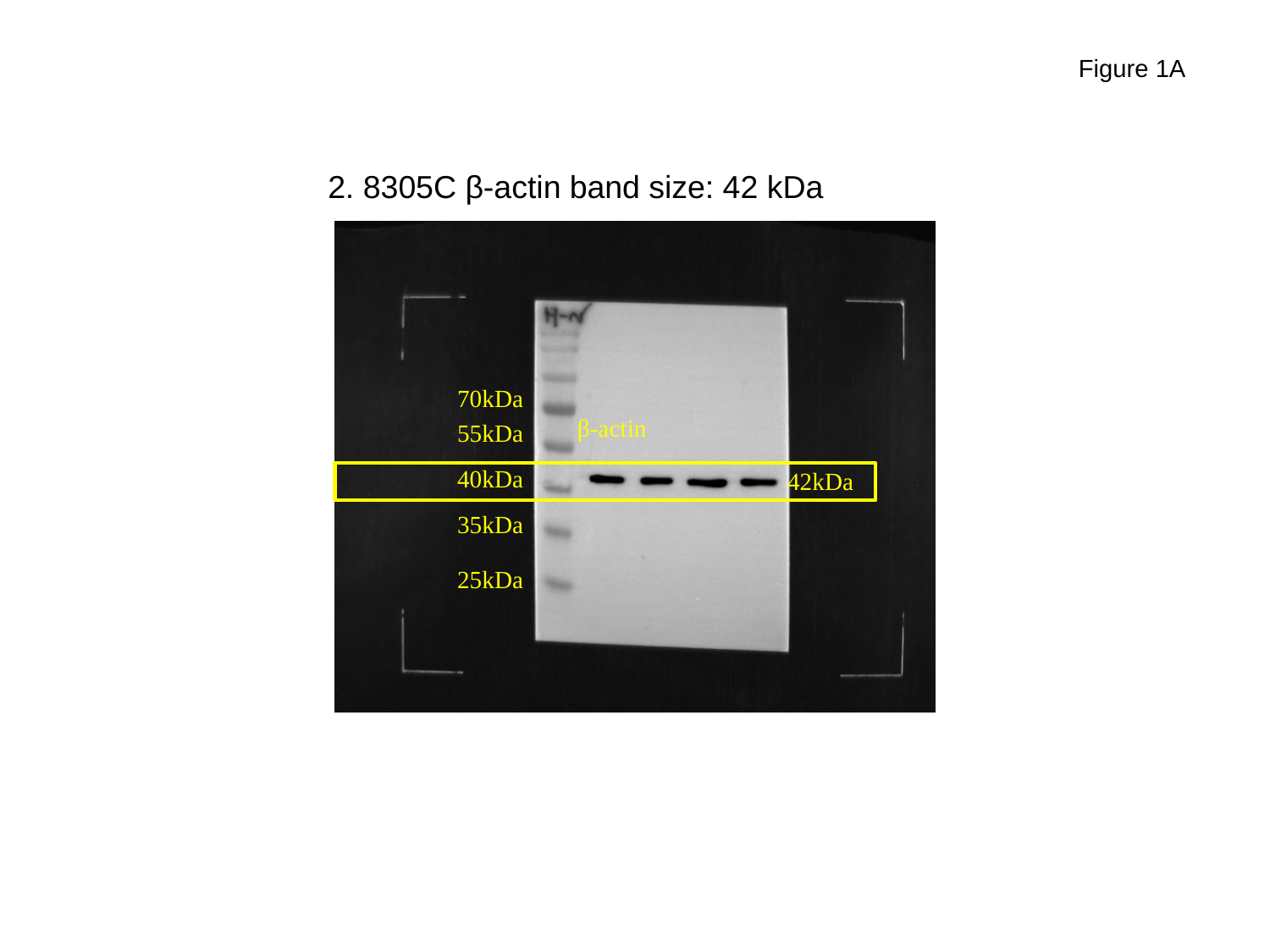

Figure 1A
2. 8305C β-actin band size: 42 kDa
70kDa
β-actin
55kDa
40kDa
42kDa
35kDa
25kDa

## Slide 3
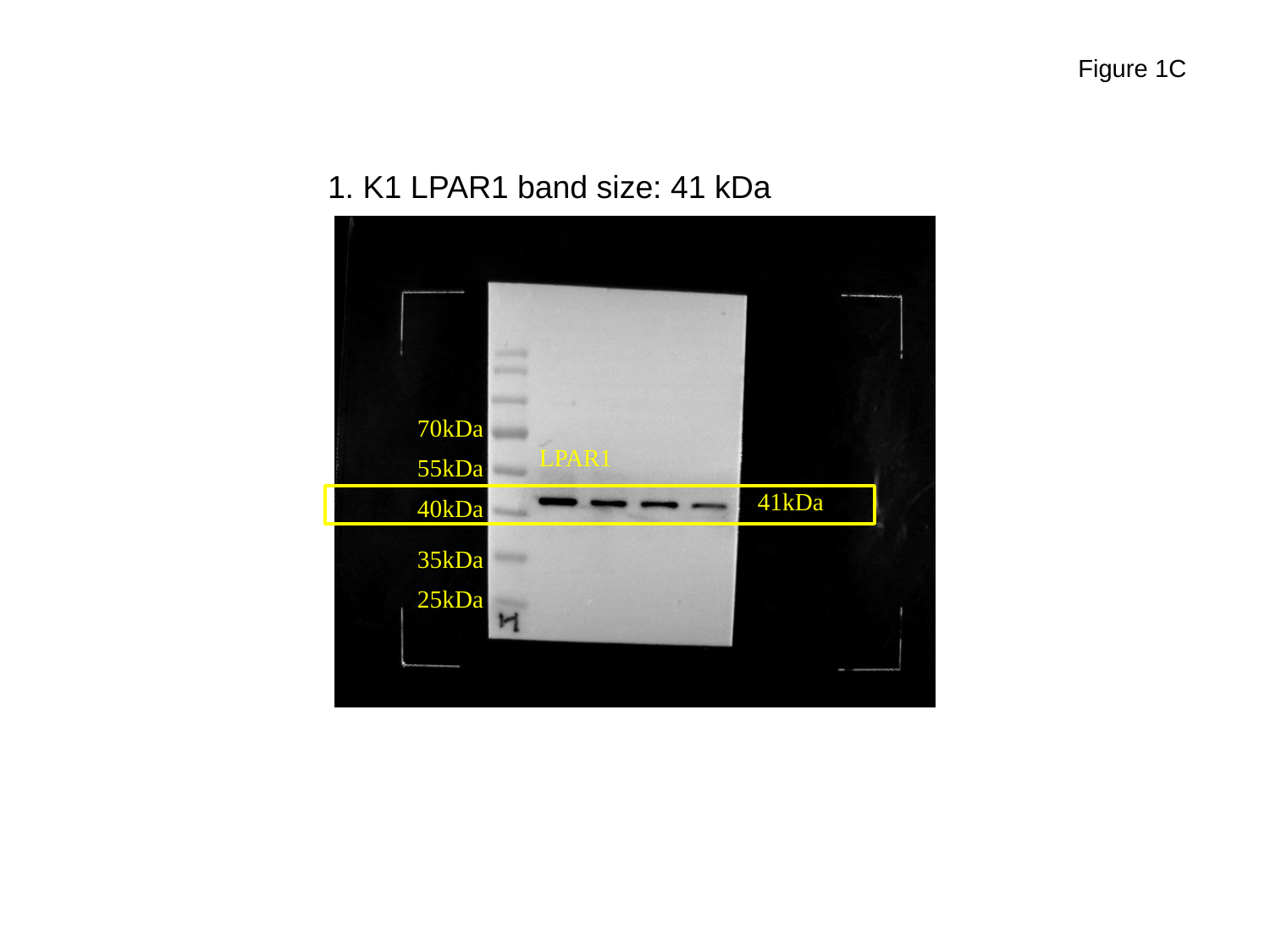

Figure 1C
1. K1 LPAR1 band size: 41 kDa
70kDa
LPAR1
55kDa
41kDa
40kDa
35kDa
25kDa

## Slide 4
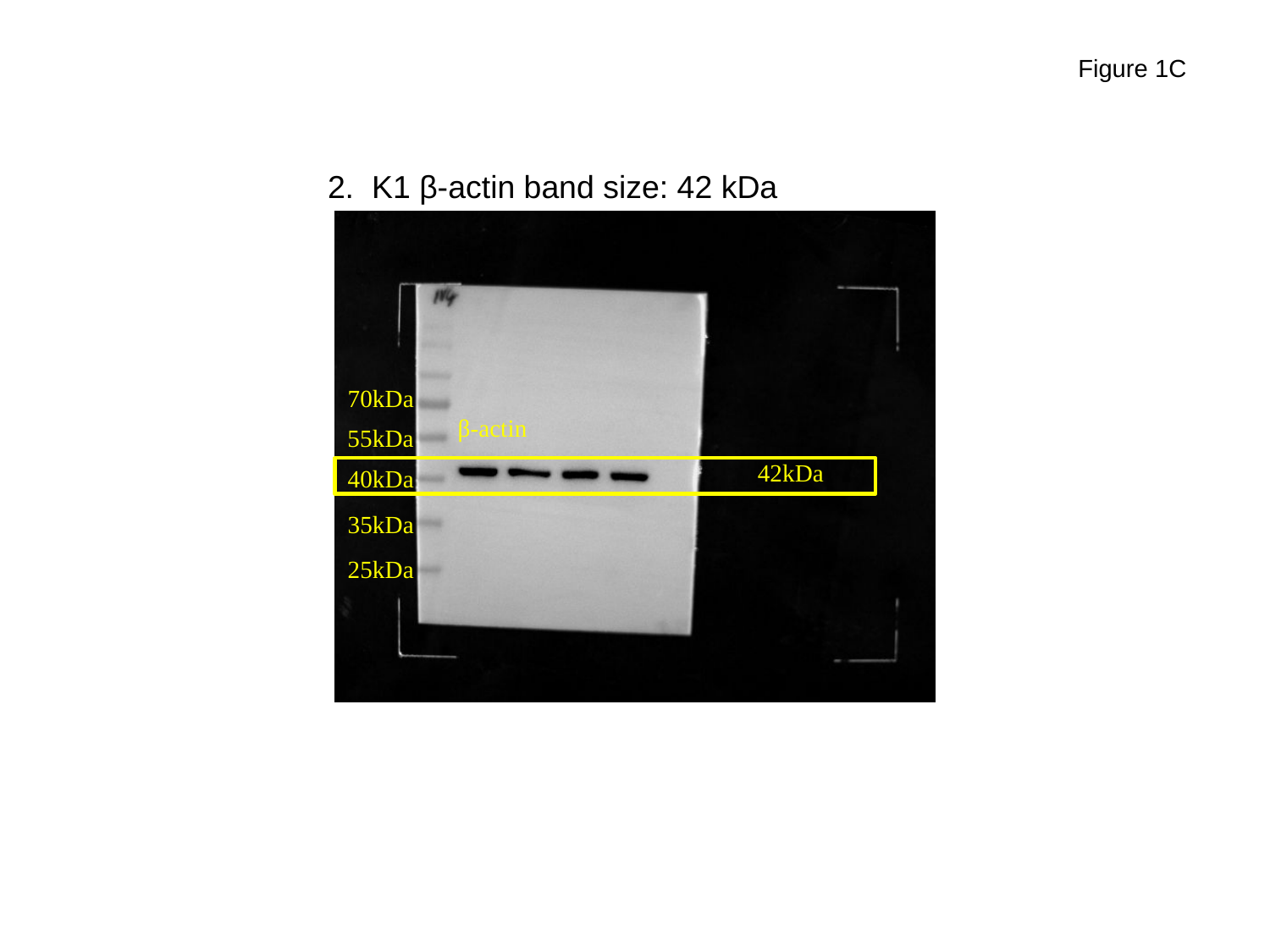

Figure 1C
2. K1 β-actin band size: 42 kDa
70kDa
β-actin
55kDa
42kDa
40kDa
35kDa
25kDa

## Slide 5
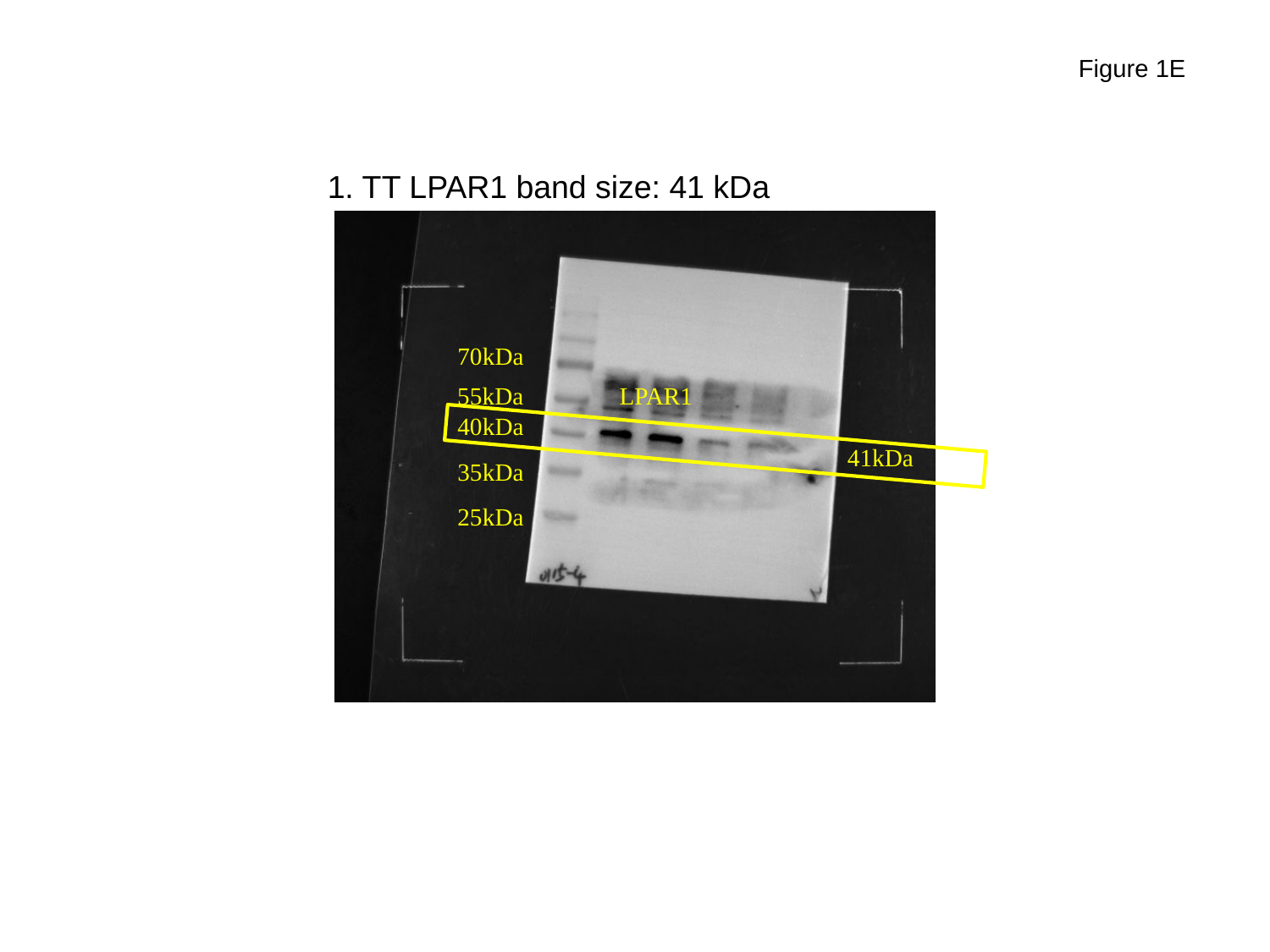

Figure 1E
1. TT LPAR1 band size: 41 kDa
70kDa
LPAR1
55kDa
40kDa
41kDa
35kDa
25kDa

## Slide 6
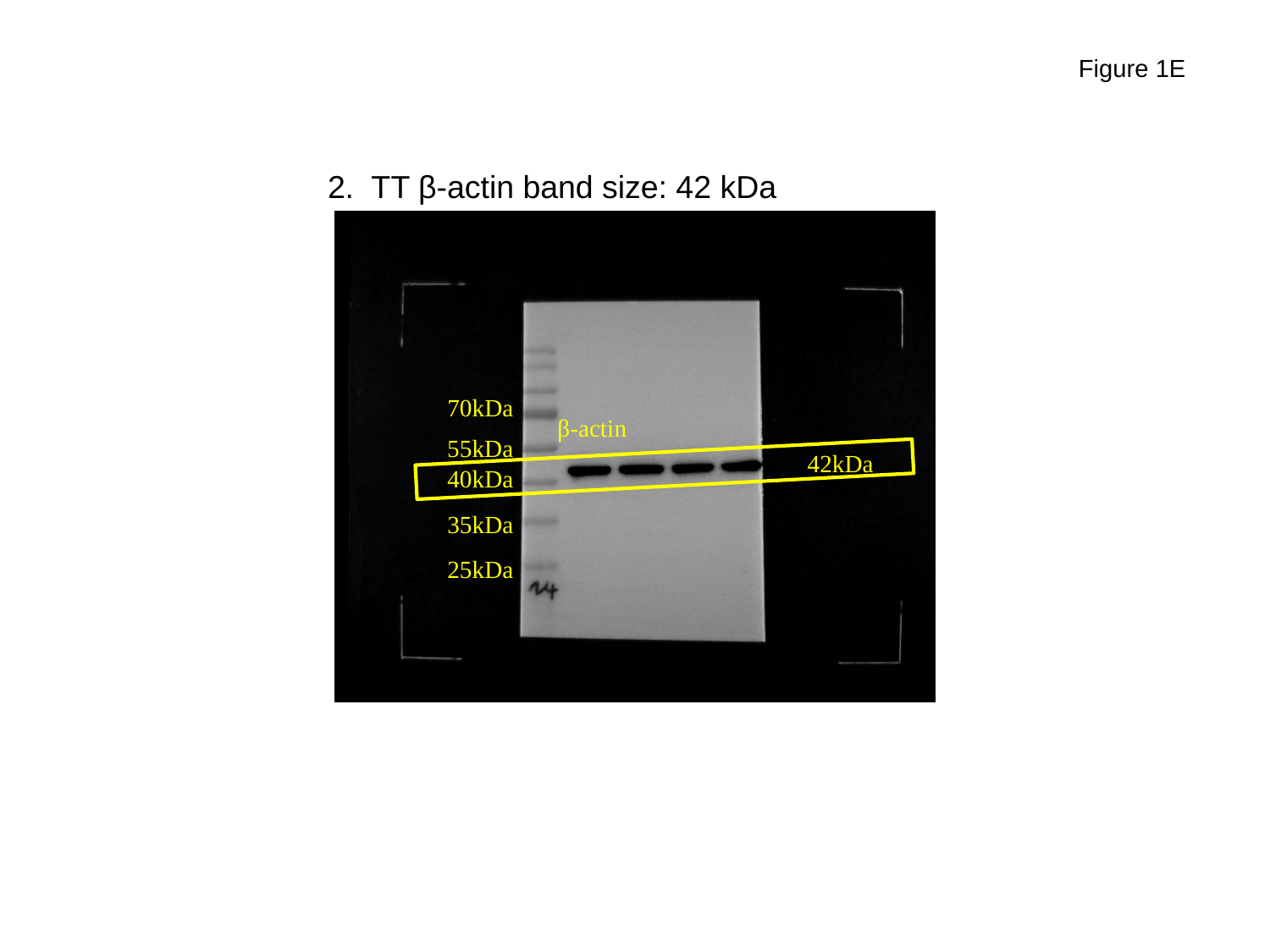

Figure 1E
2. TT β-actin band size: 42 kDa
70kDa
β-actin
55kDa
42kDa
40kDa
35kDa
25kDa

## Slide 7
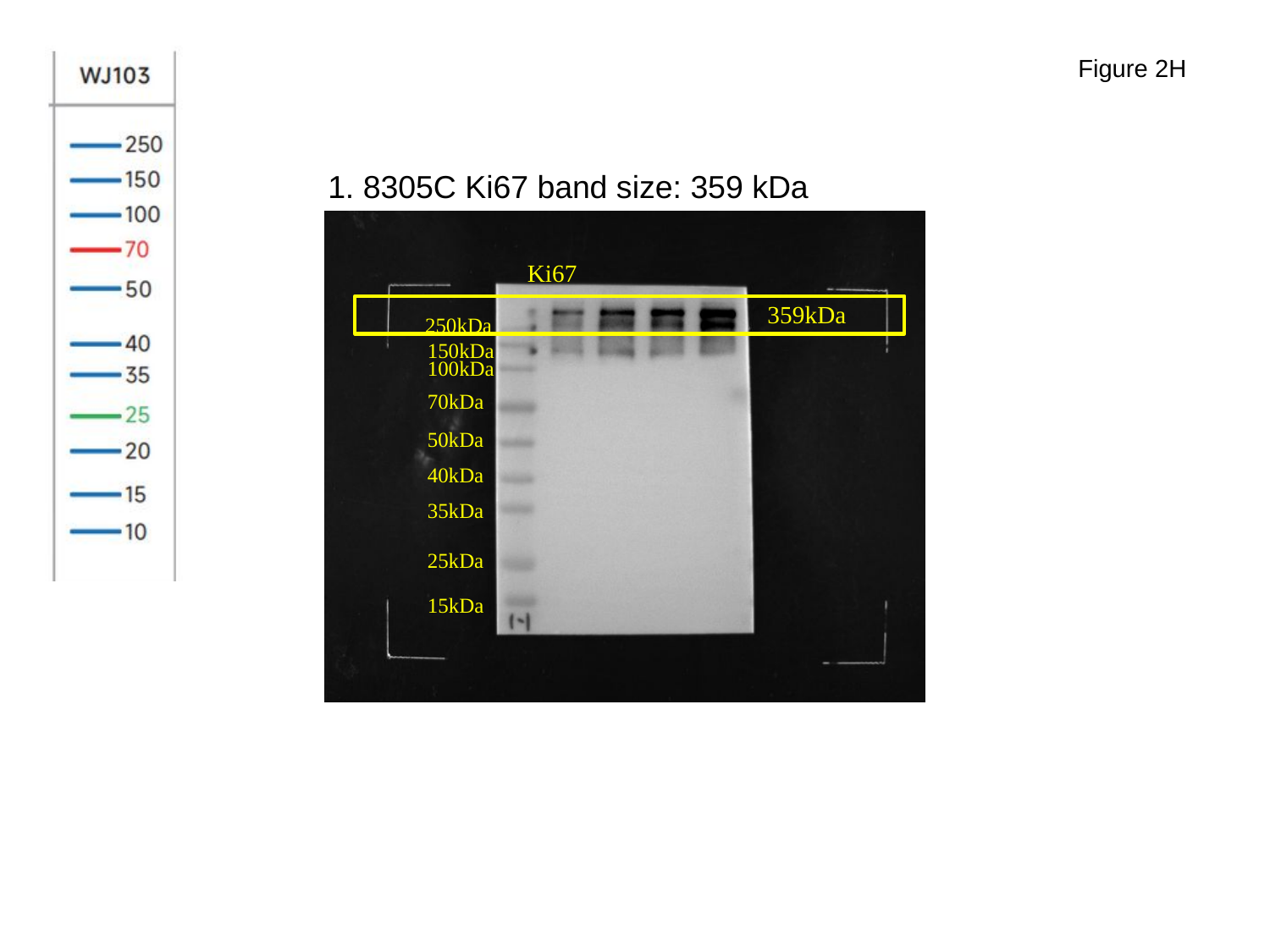

Figure 2H
1. 8305C Ki67 band size: 359 kDa
Ki67
359kDa
250kDa
150kDa
100kDa
70kDa
50kDa
40kDa
35kDa
25kDa
15kDa

## Slide 8
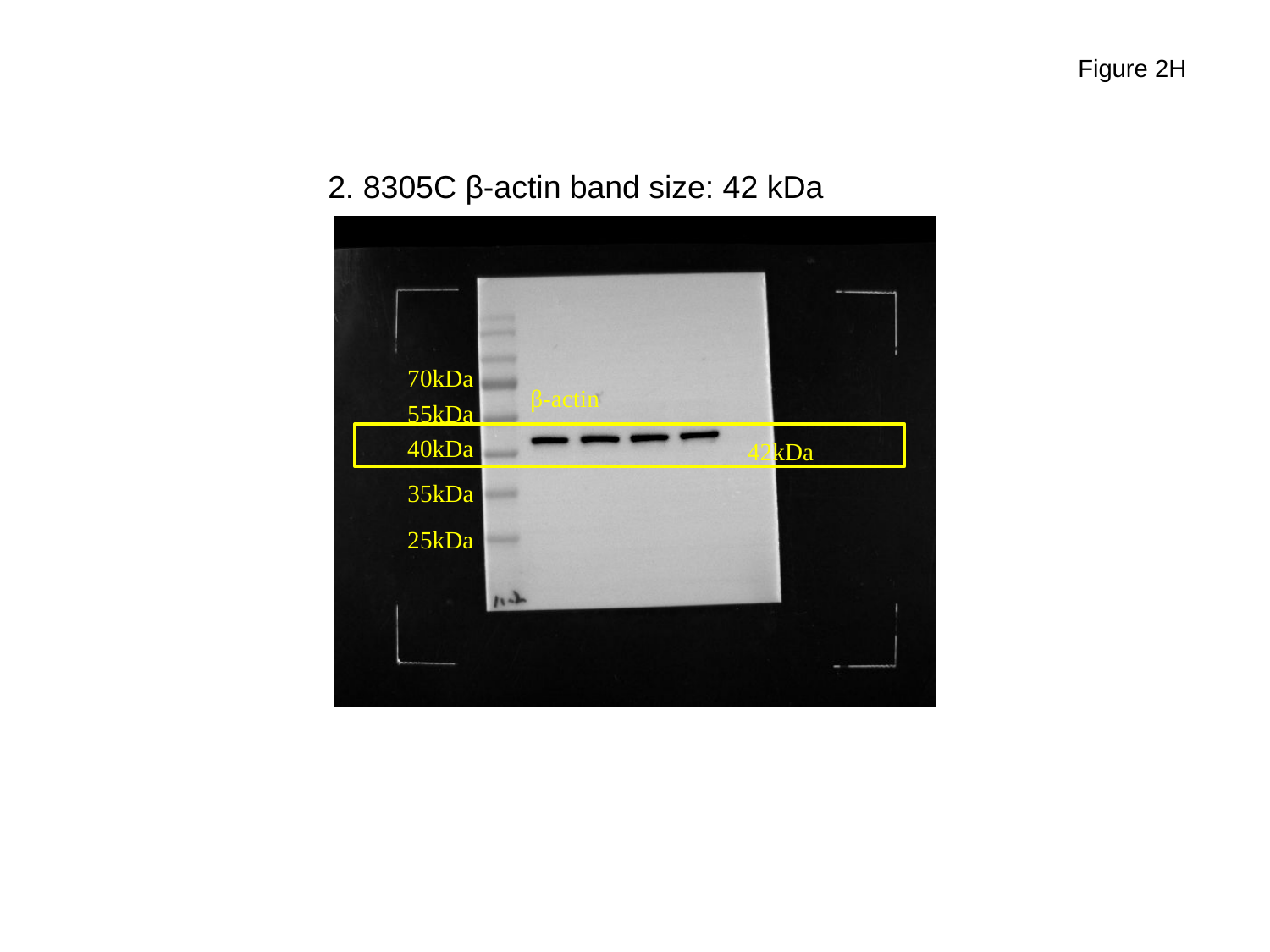

Figure 2H
2. 8305C β-actin band size: 42 kDa
70kDa
β-actin
55kDa
40kDa
42kDa
35kDa
25kDa

## Slide 9
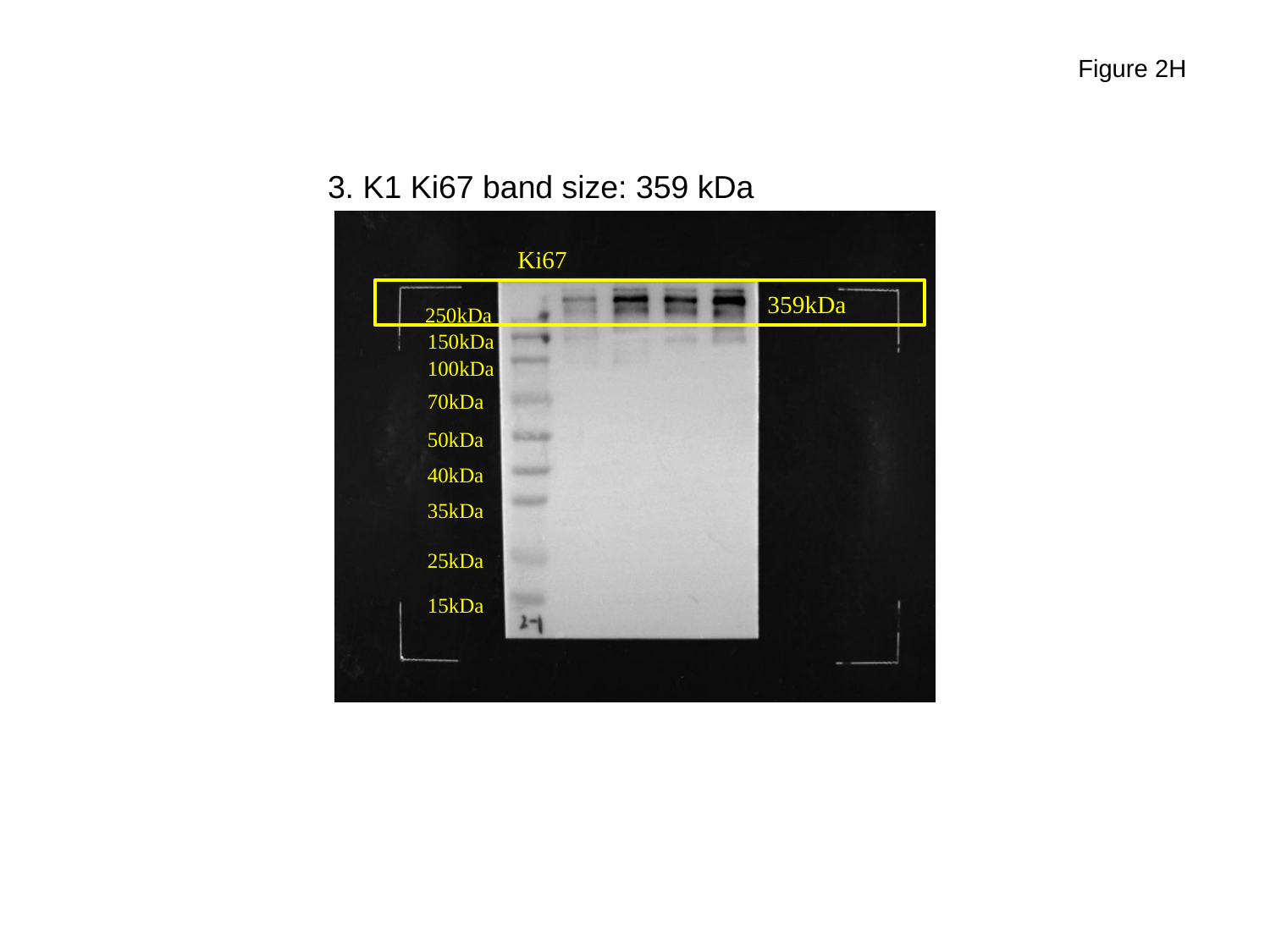

Figure 2H
3. K1 Ki67 band size: 359 kDa
Ki67
359kDa
250kDa
150kDa
100kDa
70kDa
50kDa
40kDa
35kDa
25kDa
15kDa

## Slide 10
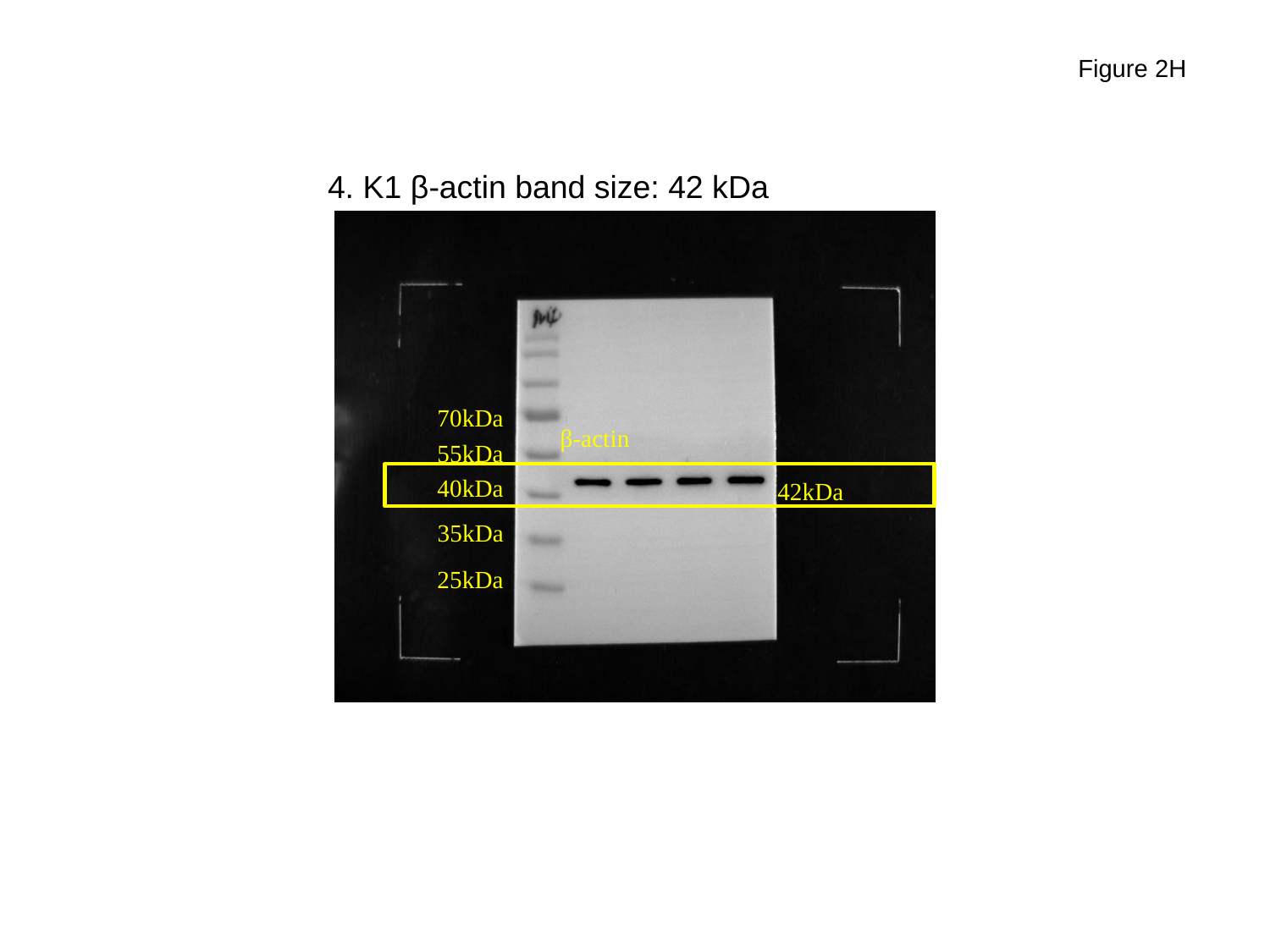

Figure 2H
4. K1 β-actin band size: 42 kDa
70kDa
β-actin
55kDa
40kDa
42kDa
35kDa
25kDa

## Slide 11
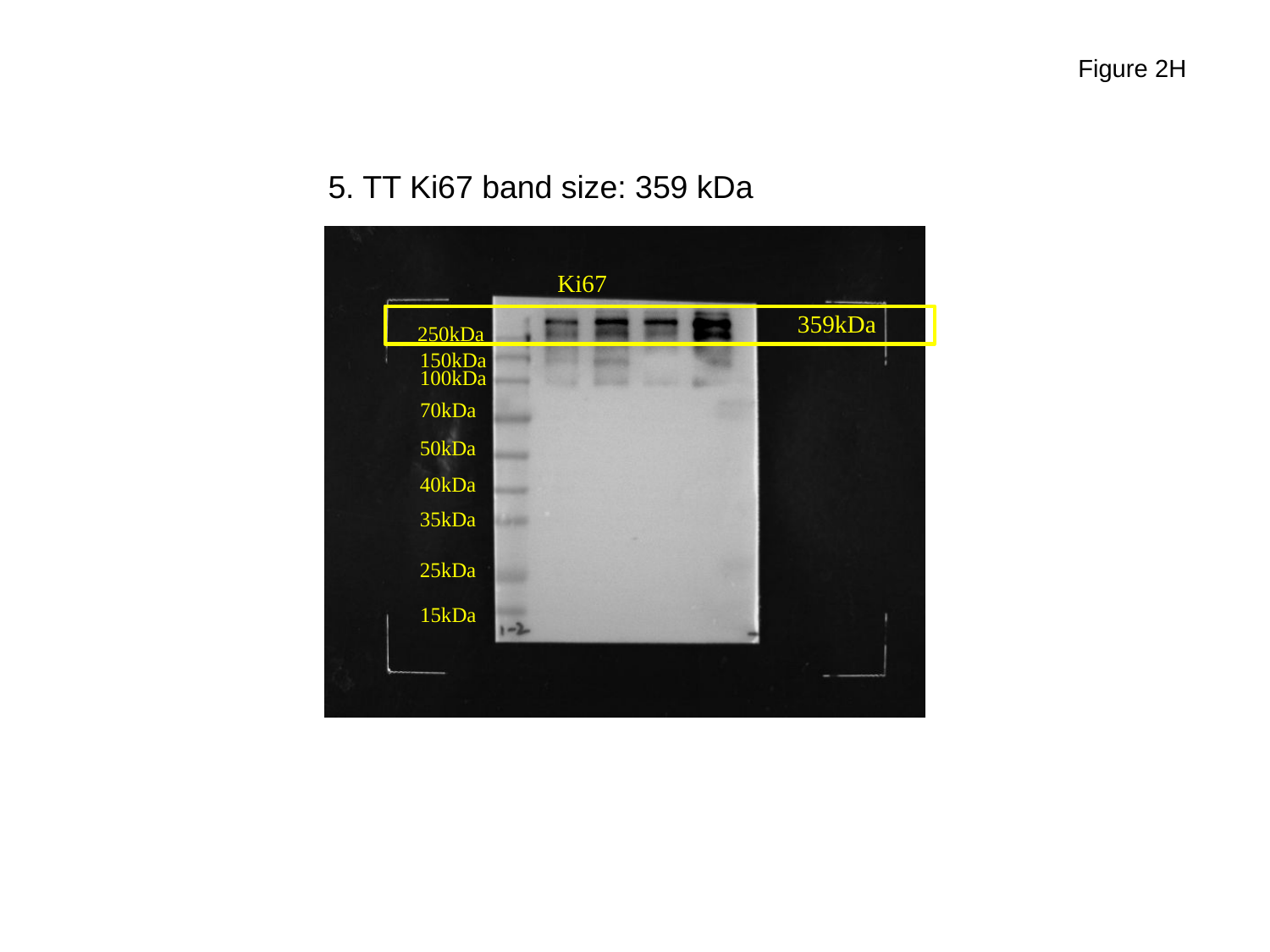

Figure 2H
5. TT Ki67 band size: 359 kDa
Ki67
359kDa
250kDa
150kDa
100kDa
70kDa
50kDa
40kDa
35kDa
25kDa
15kDa

## Slide 12
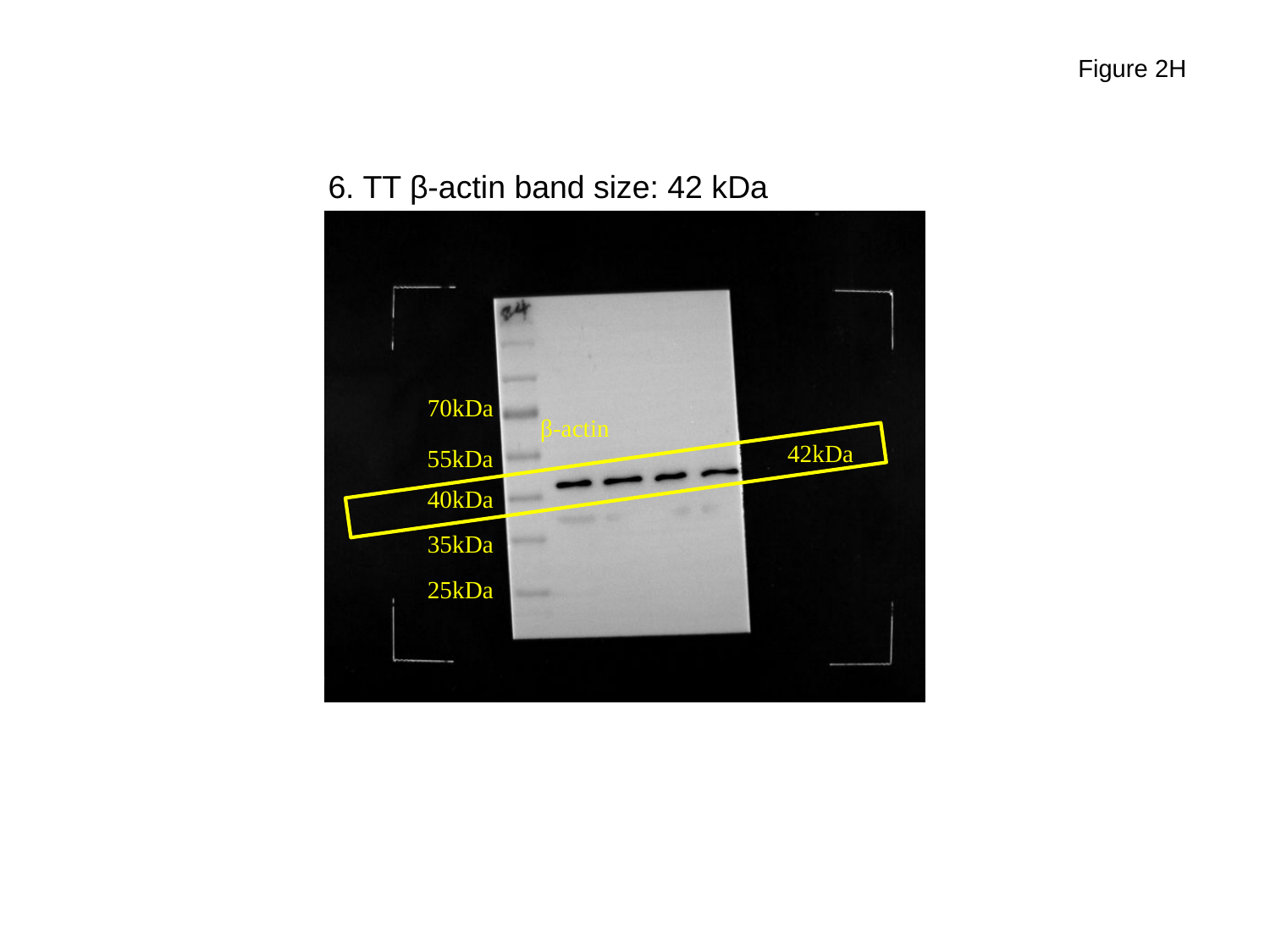

Figure 2H
6. TT β-actin band size: 42 kDa
70kDa
β-actin
42kDa
55kDa
40kDa
35kDa
25kDa

## Slide 13
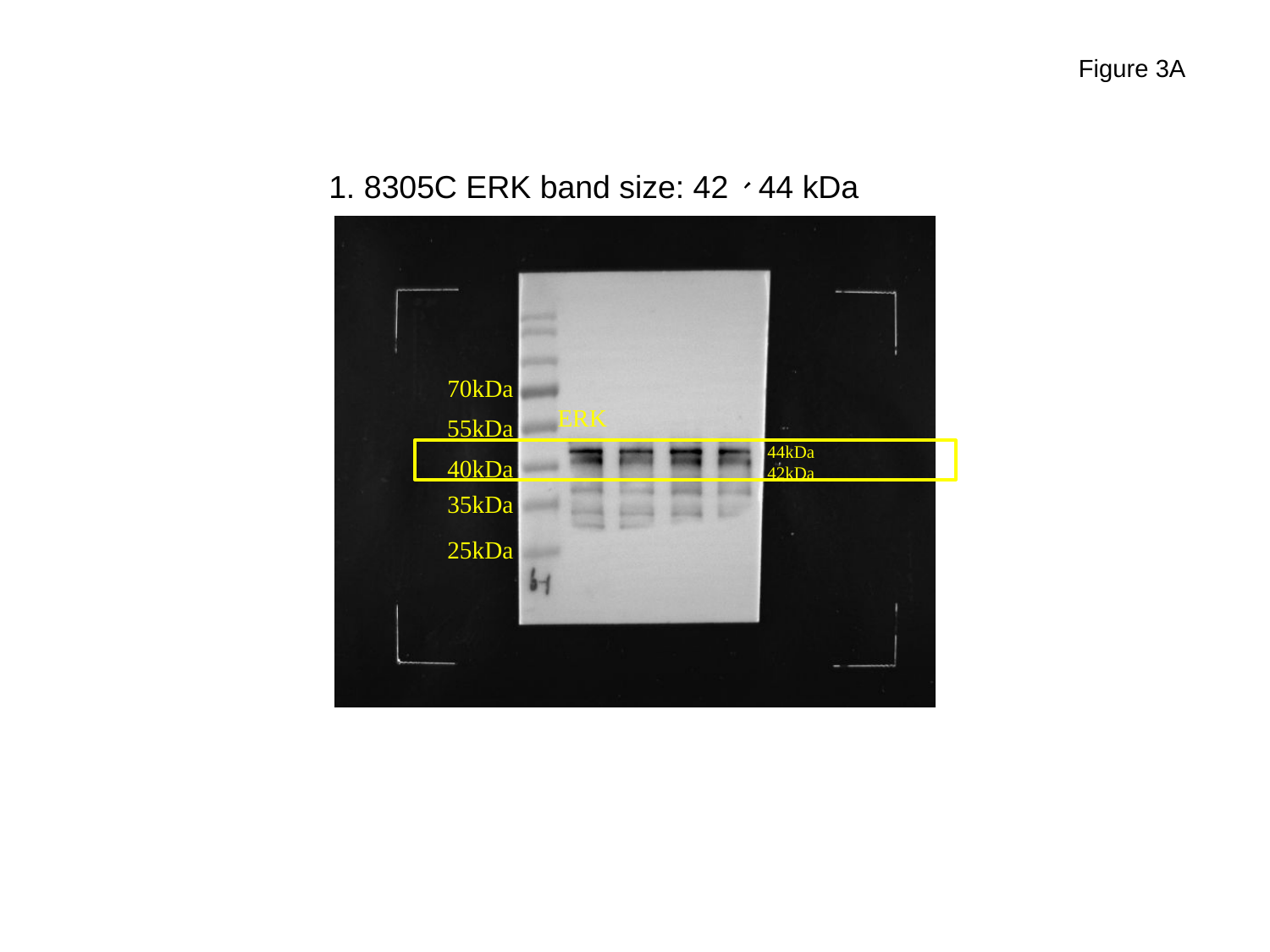

Figure 3A
1. 8305C ERK band size: 42、44 kDa
70kDa
ERK
55kDa
44kDa
42kDa
40kDa
35kDa
25kDa

## Slide 14
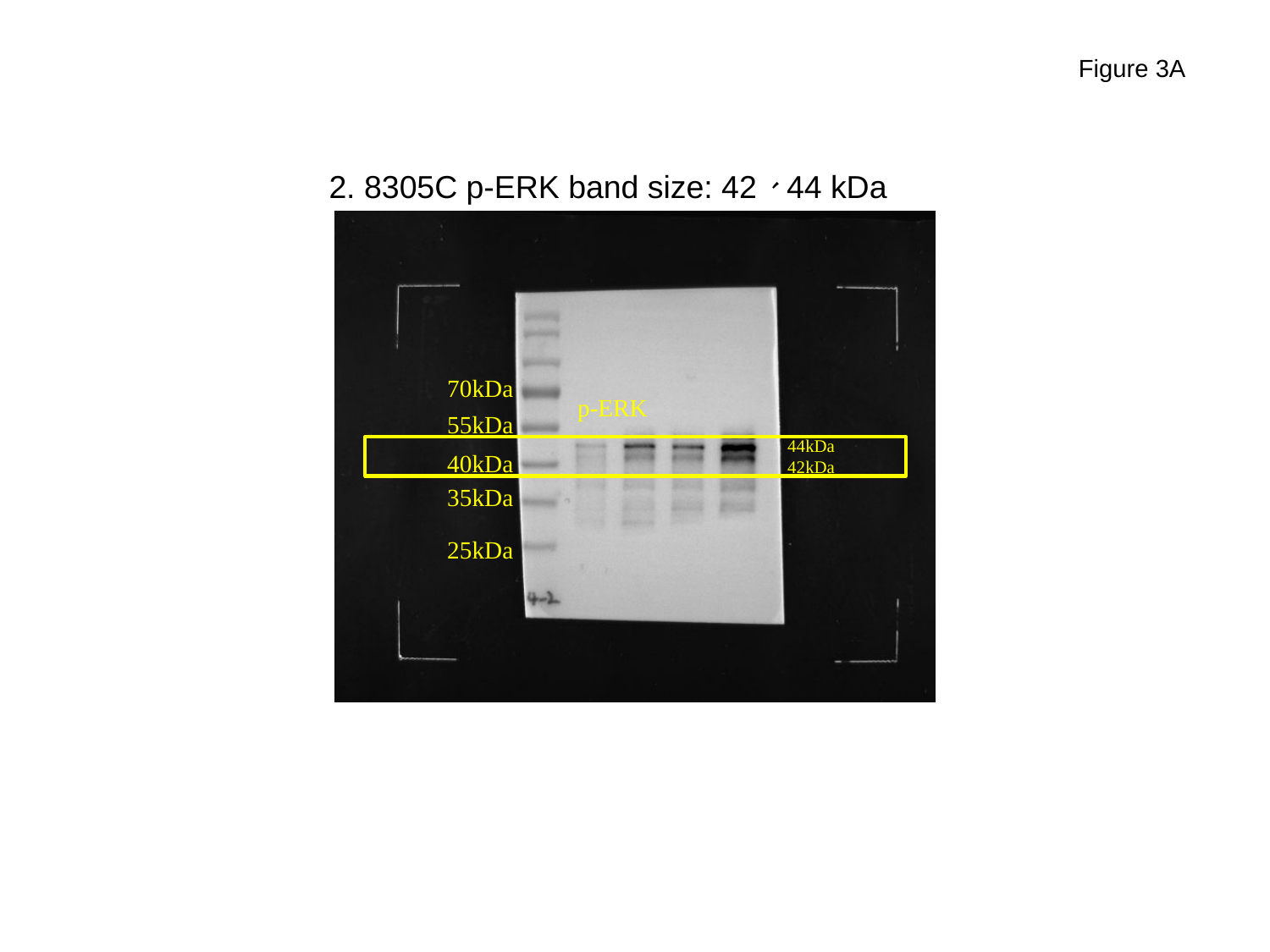

Figure 3A
2. 8305C p-ERK band size: 42、44 kDa
70kDa
p-ERK
55kDa
44kDa
42kDa
40kDa
35kDa
25kDa

## Slide 15
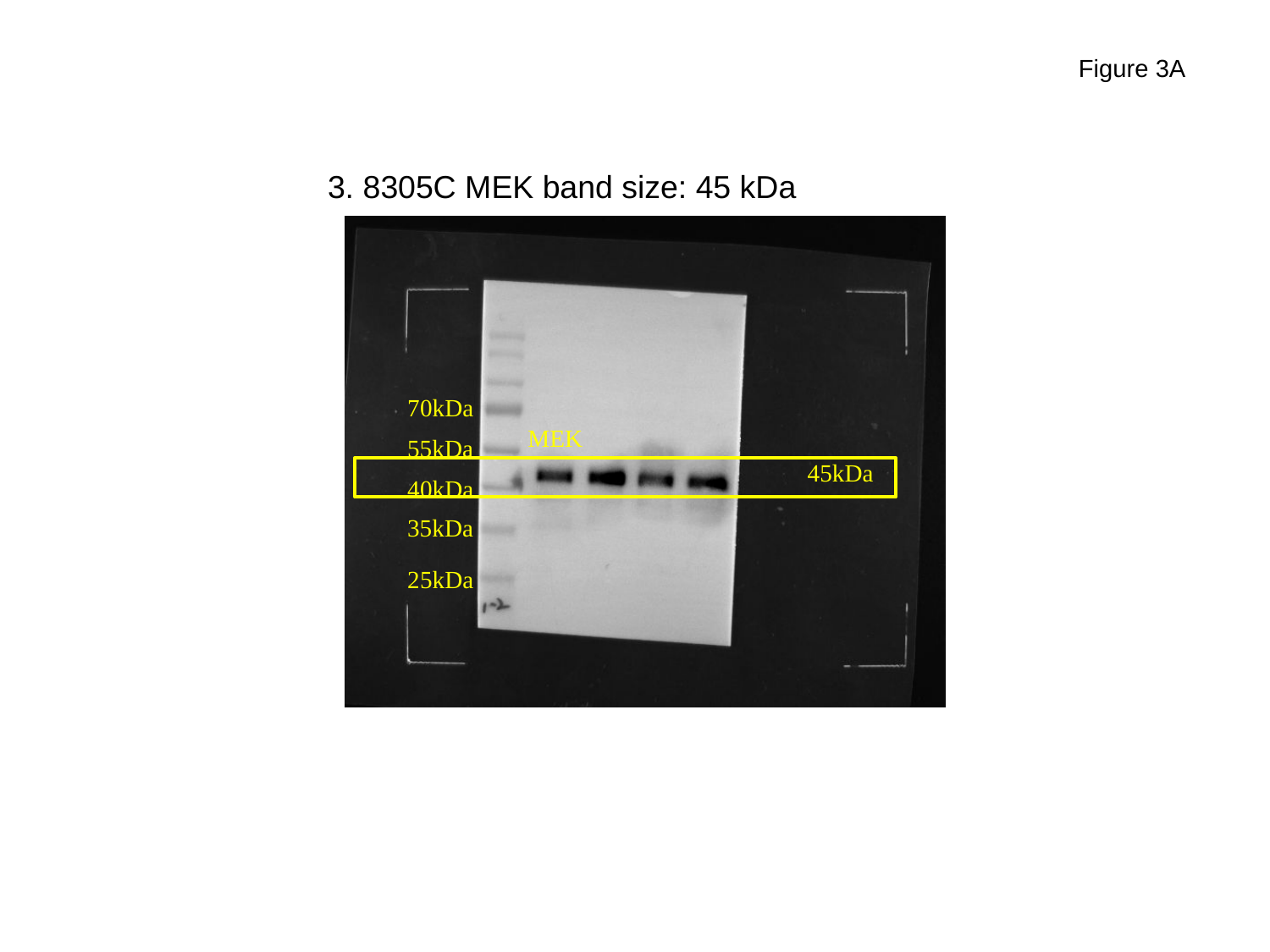

Figure 3A
3. 8305C MEK band size: 45 kDa
70kDa
MEK
55kDa
45kDa
40kDa
35kDa
25kDa

## Slide 16
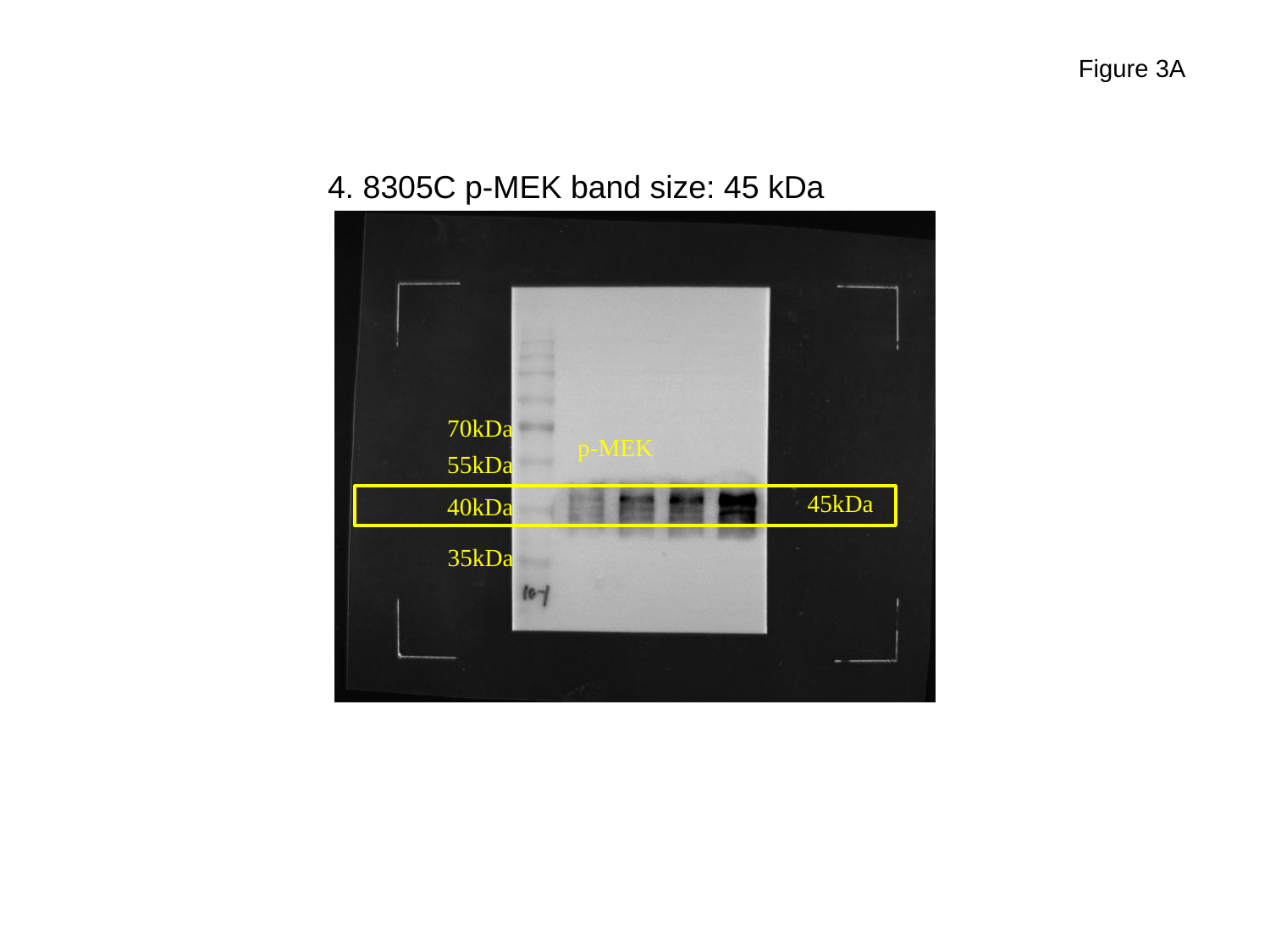

Figure 3A
4. 8305C p-MEK band size: 45 kDa
70kDa
p-MEK
55kDa
45kDa
40kDa
35kDa

## Slide 17
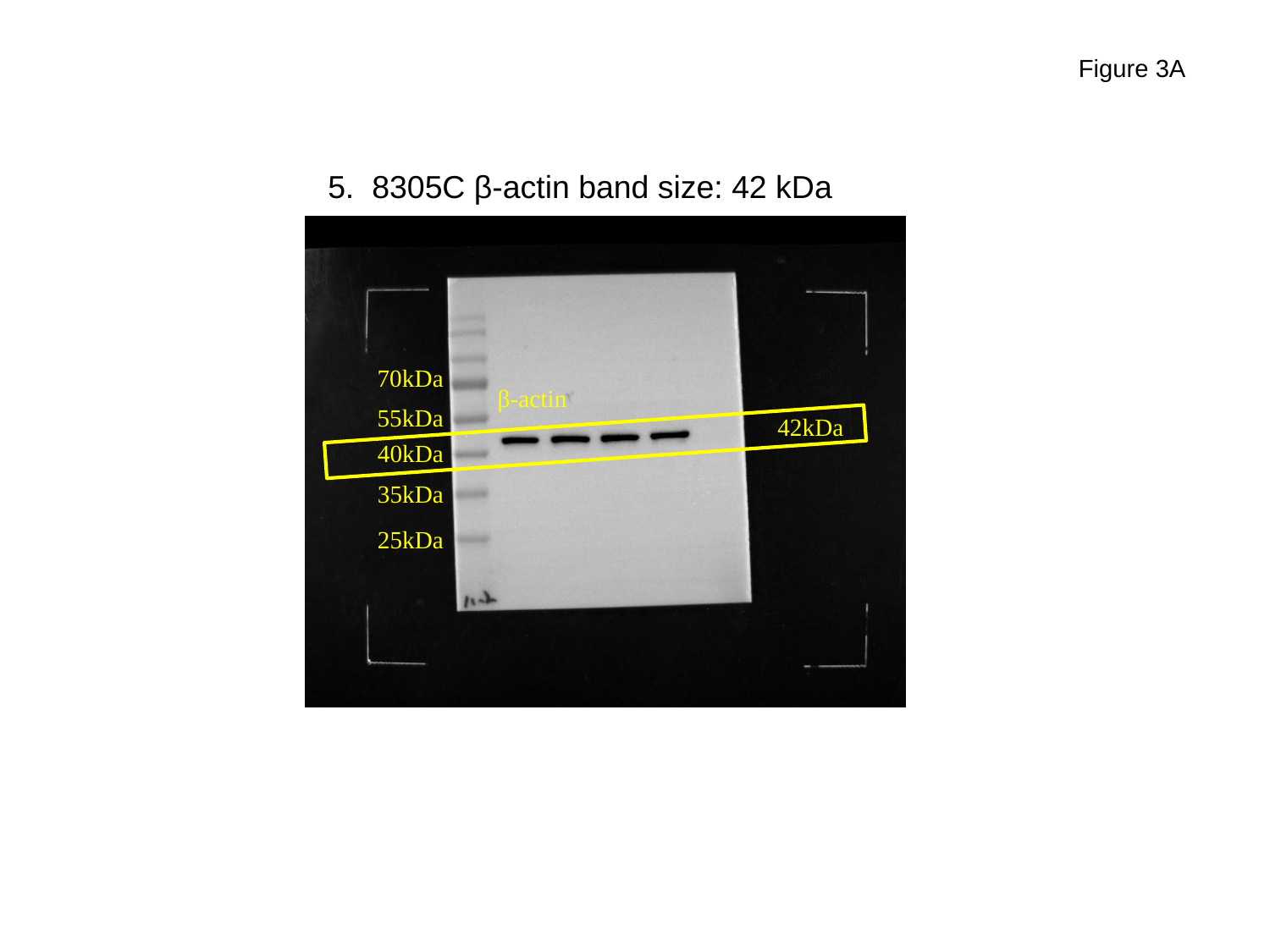

Figure 3A
5. 8305C β-actin band size: 42 kDa
70kDa
β-actin
55kDa
42kDa
40kDa
35kDa
25kDa

## Slide 18
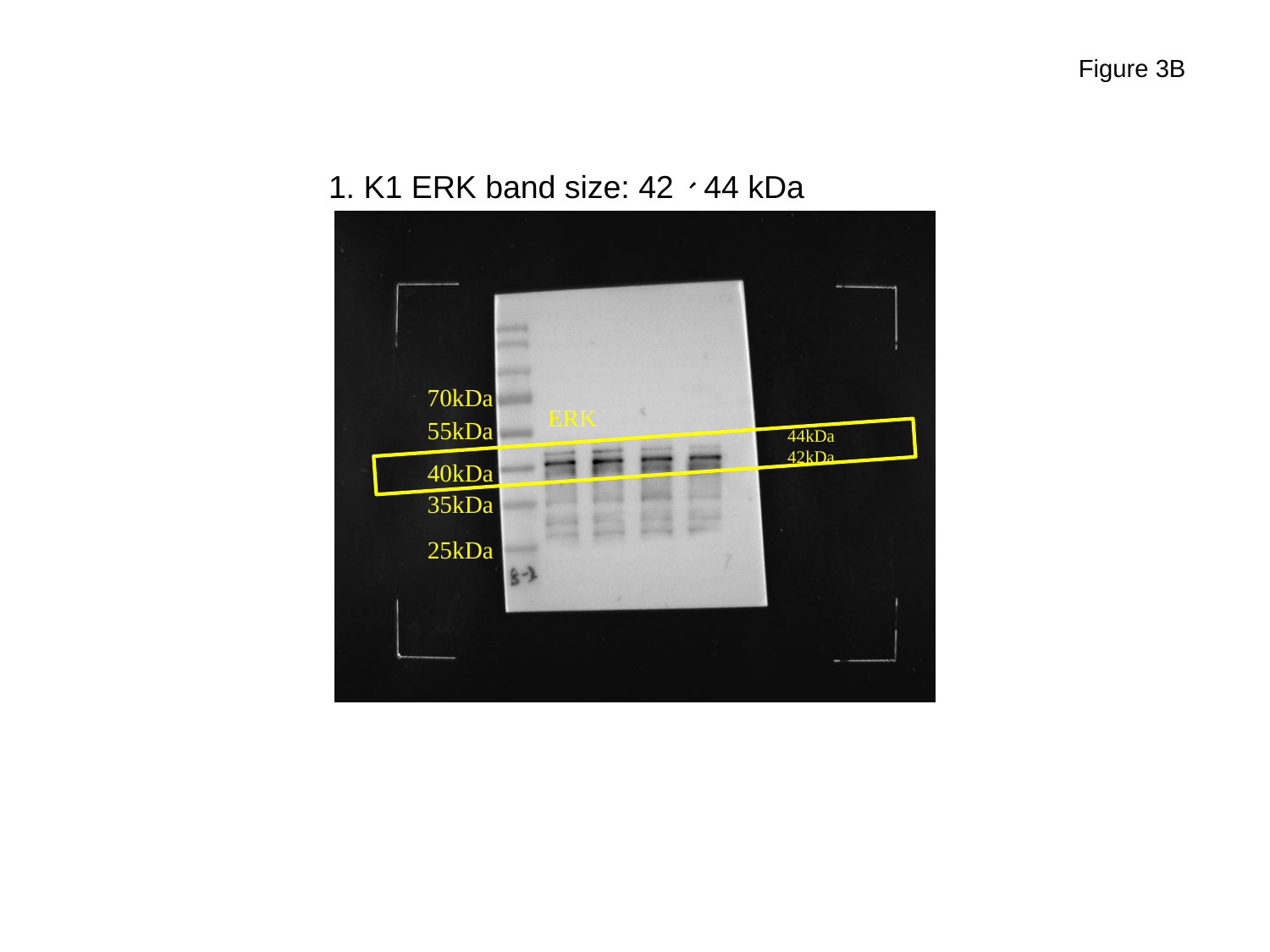

Figure 3B
1. K1 ERK band size: 42、44 kDa
70kDa
ERK
55kDa
44kDa
42kDa
40kDa
35kDa
25kDa

## Slide 19
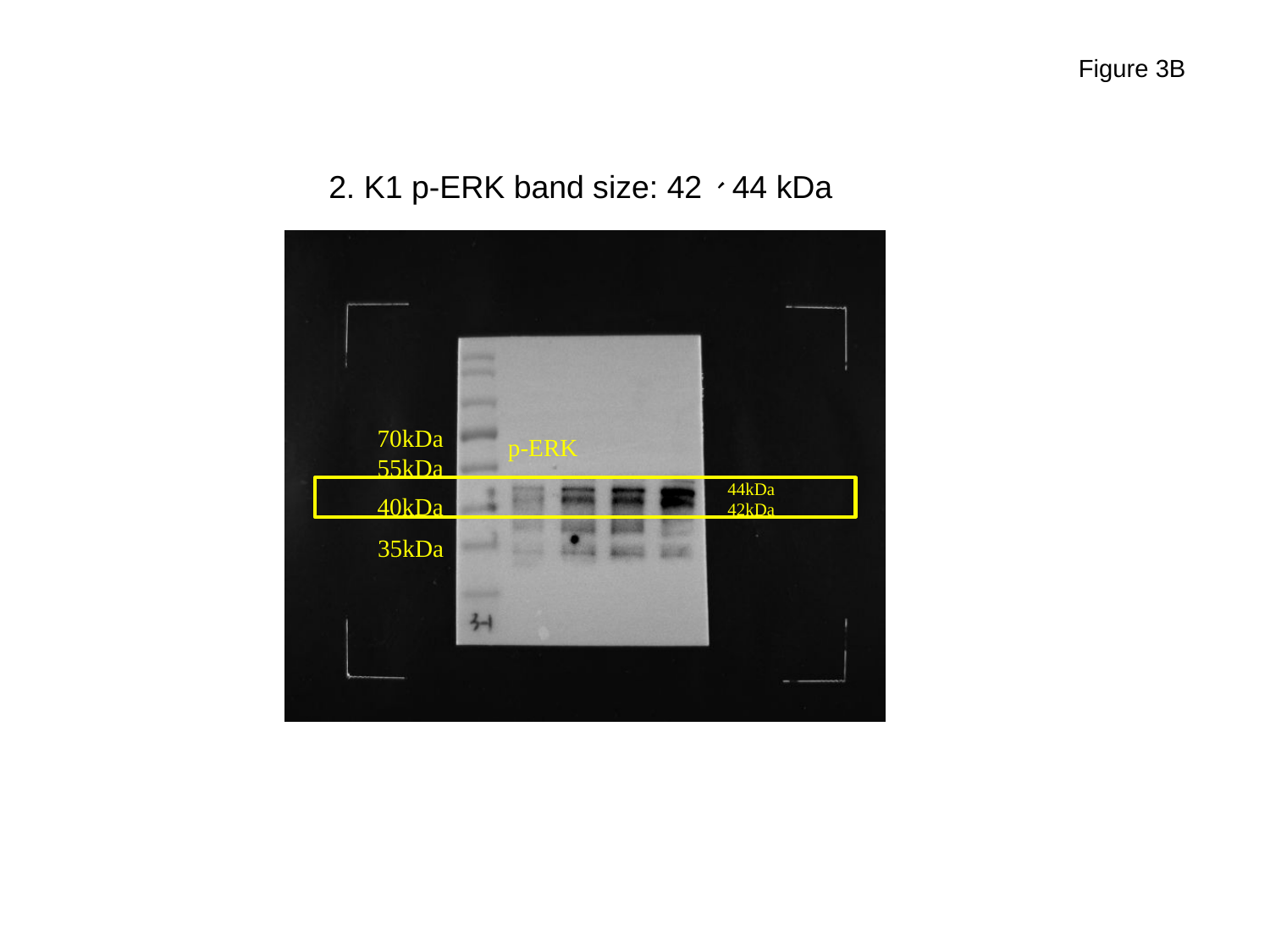

Figure 3B
2. K1 p-ERK band size: 42、44 kDa
70kDa
p-ERK
55kDa
44kDa
42kDa
40kDa
35kDa

## Slide 20
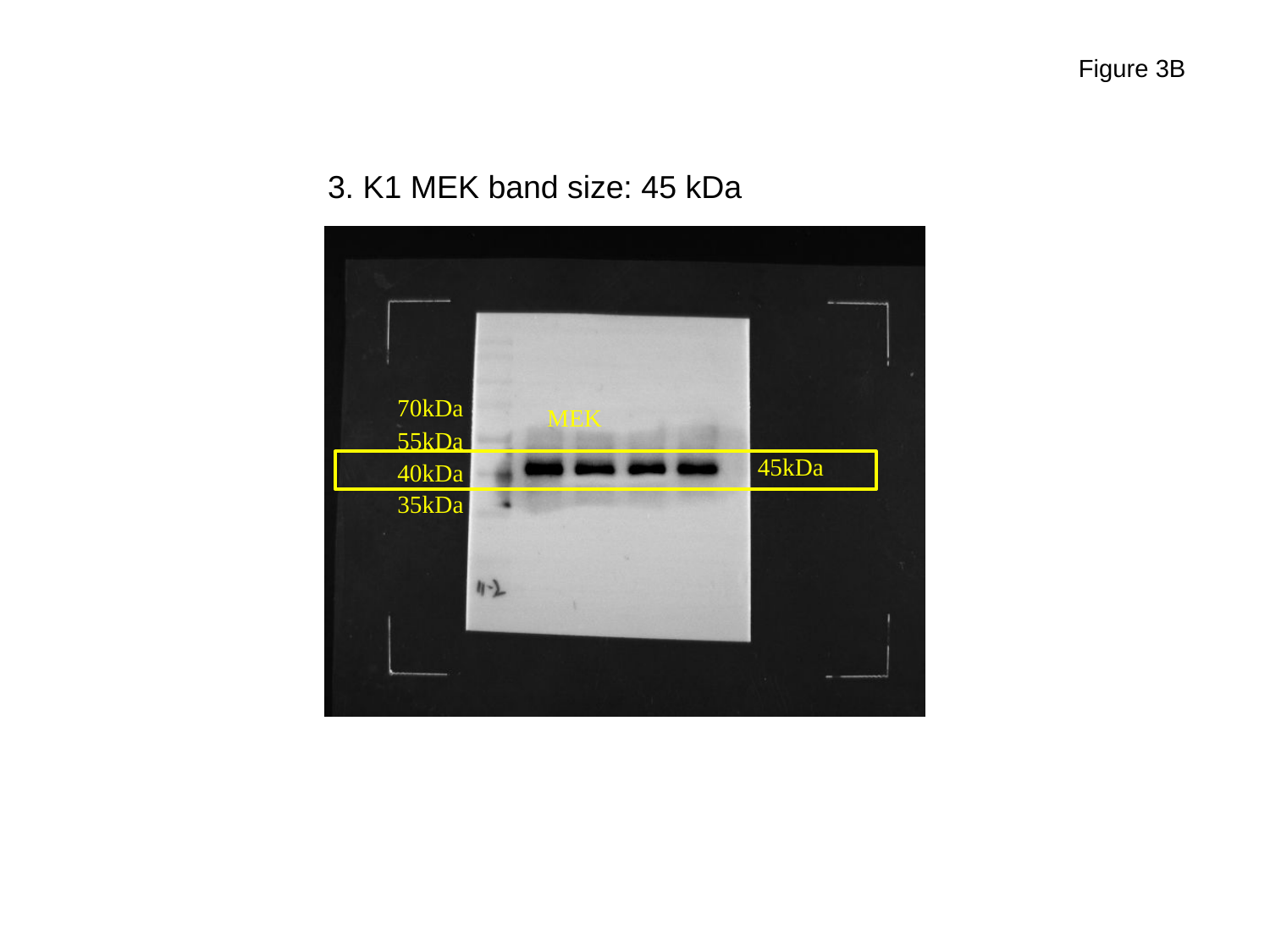

Figure 3B
3. K1 MEK band size: 45 kDa
70kDa
MEK
55kDa
45kDa
40kDa
35kDa

## Slide 21
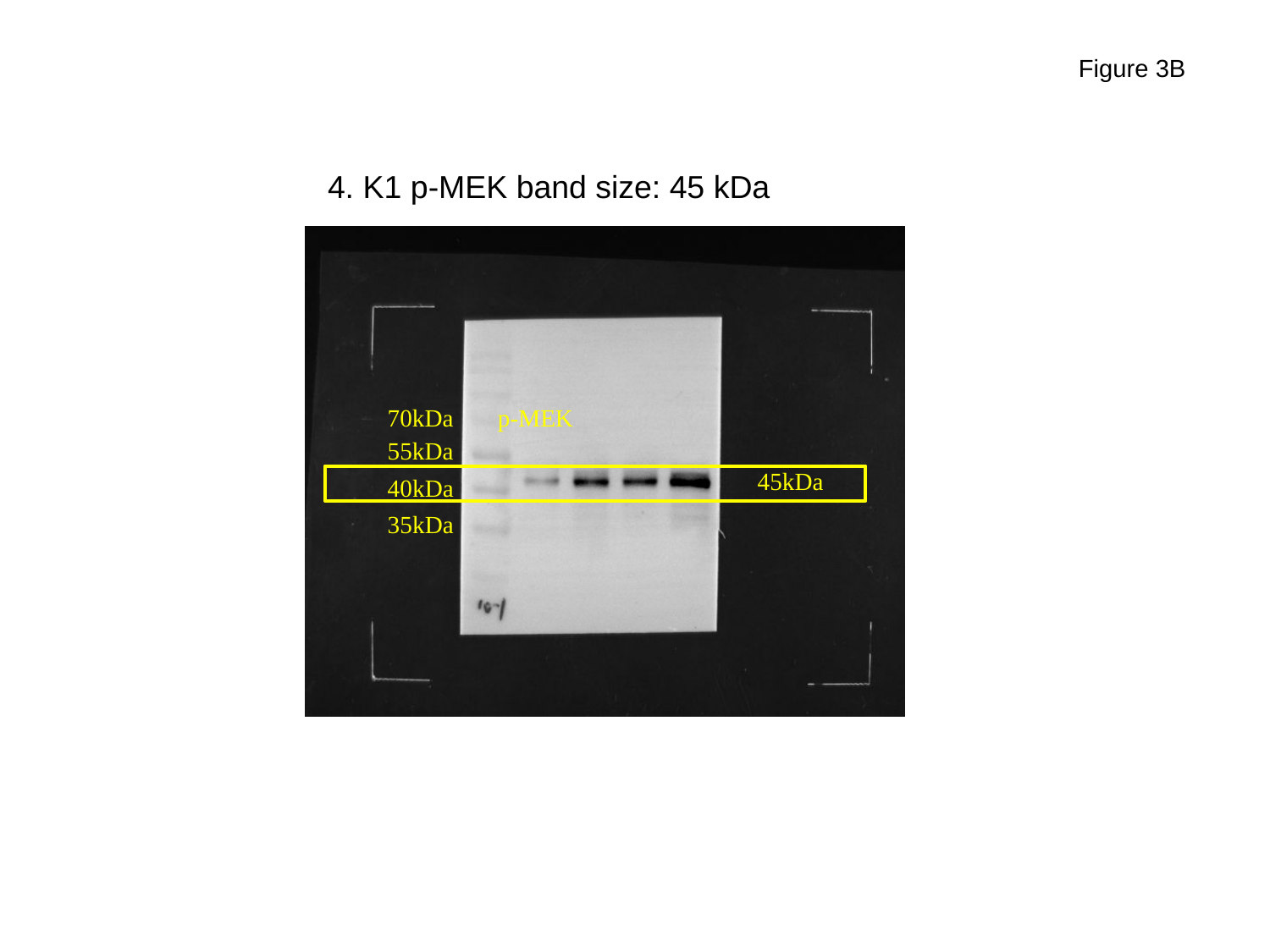

Figure 3B
4. K1 p-MEK band size: 45 kDa
70kDa
p-MEK
55kDa
45kDa
40kDa
35kDa

## Slide 22
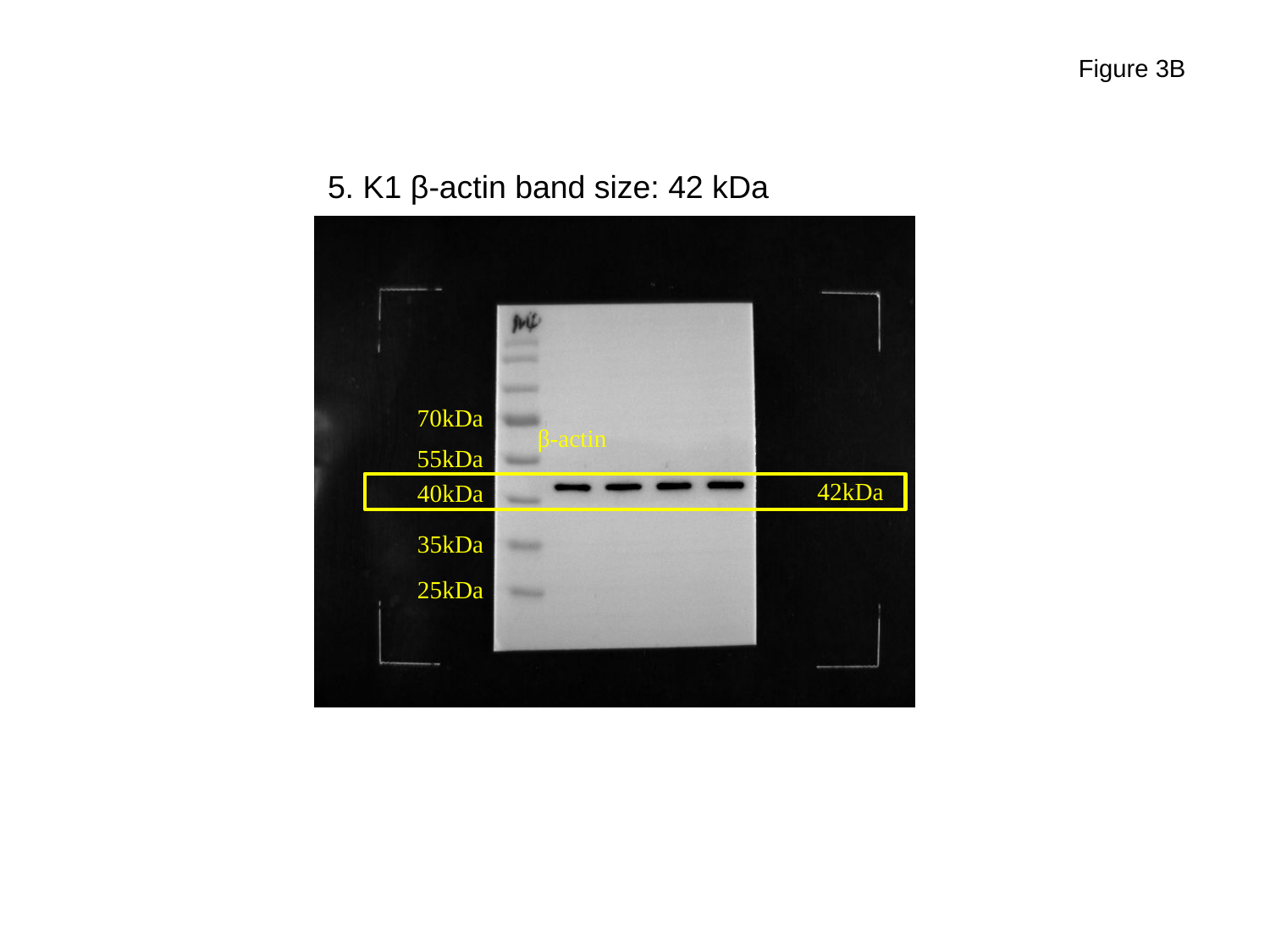

Figure 3B
5. K1 β-actin band size: 42 kDa
70kDa
β-actin
55kDa
42kDa
40kDa
35kDa
25kDa

## Slide 23
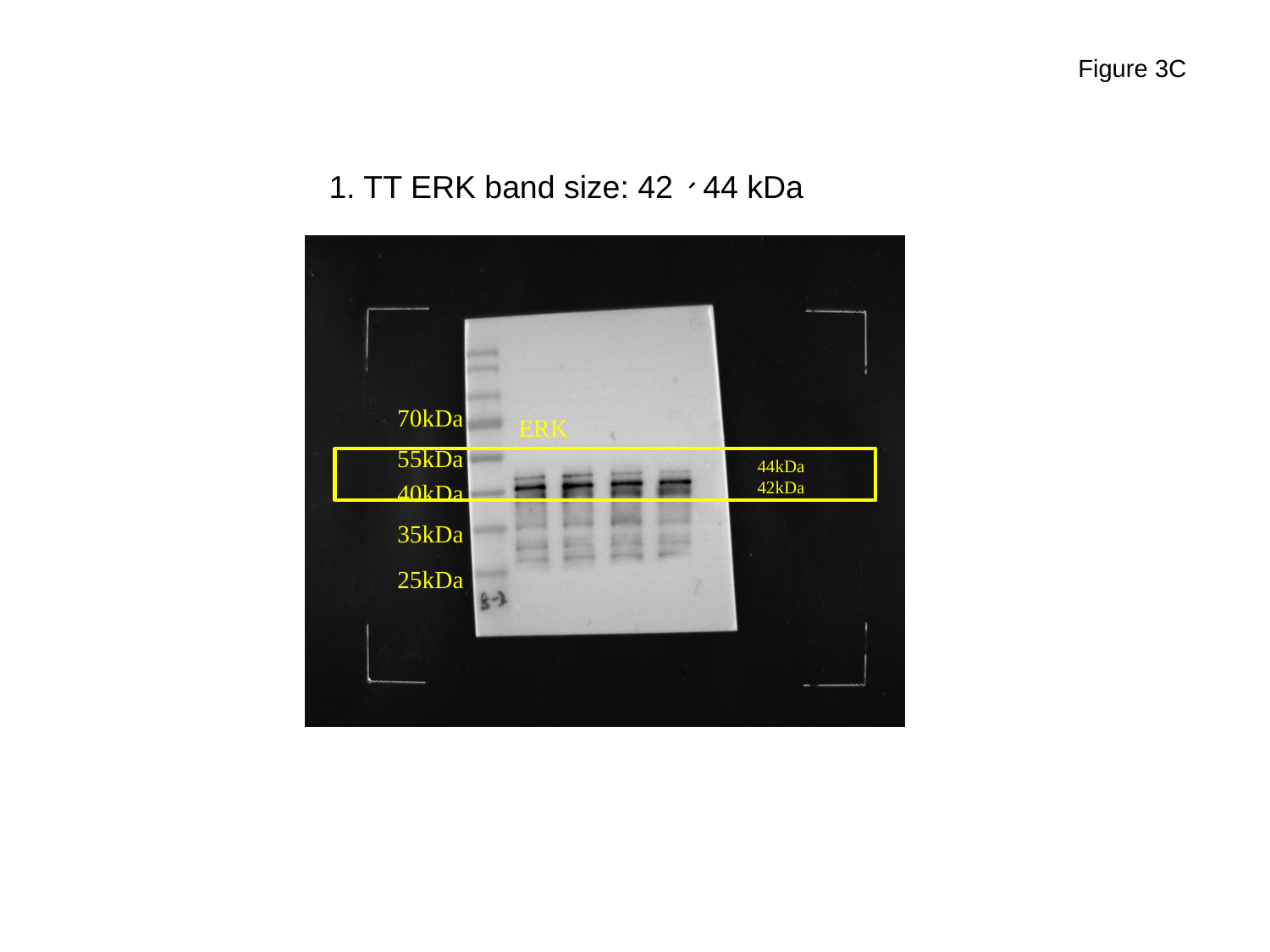

Figure 3C
1. TT ERK band size: 42、44 kDa
70kDa
ERK
55kDa
44kDa
42kDa
40kDa
35kDa
25kDa

## Slide 24
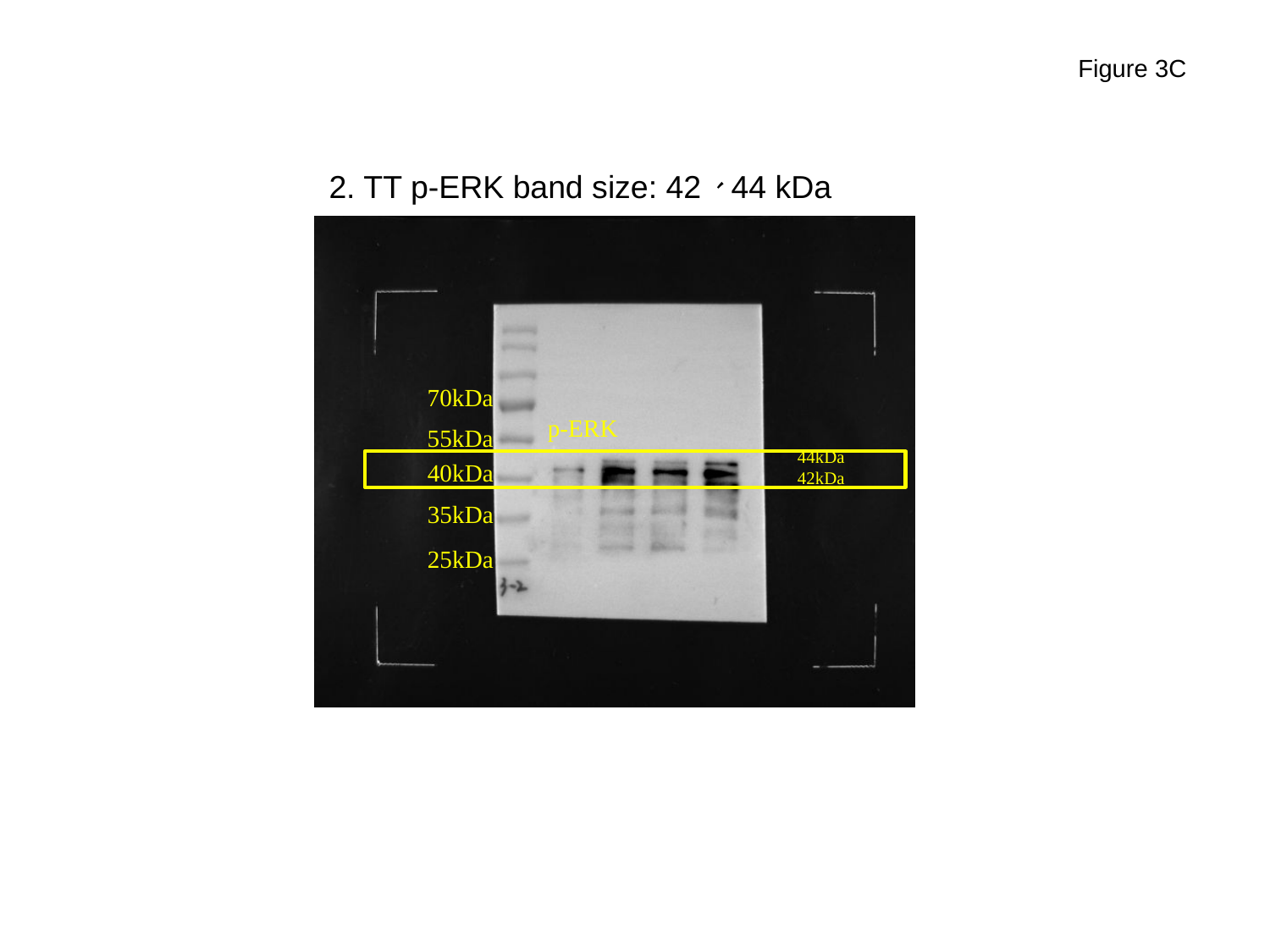

Figure 3C
2. TT p-ERK band size: 42、44 kDa
70kDa
p-ERK
55kDa
44kDa
42kDa
40kDa
35kDa
25kDa

## Slide 25
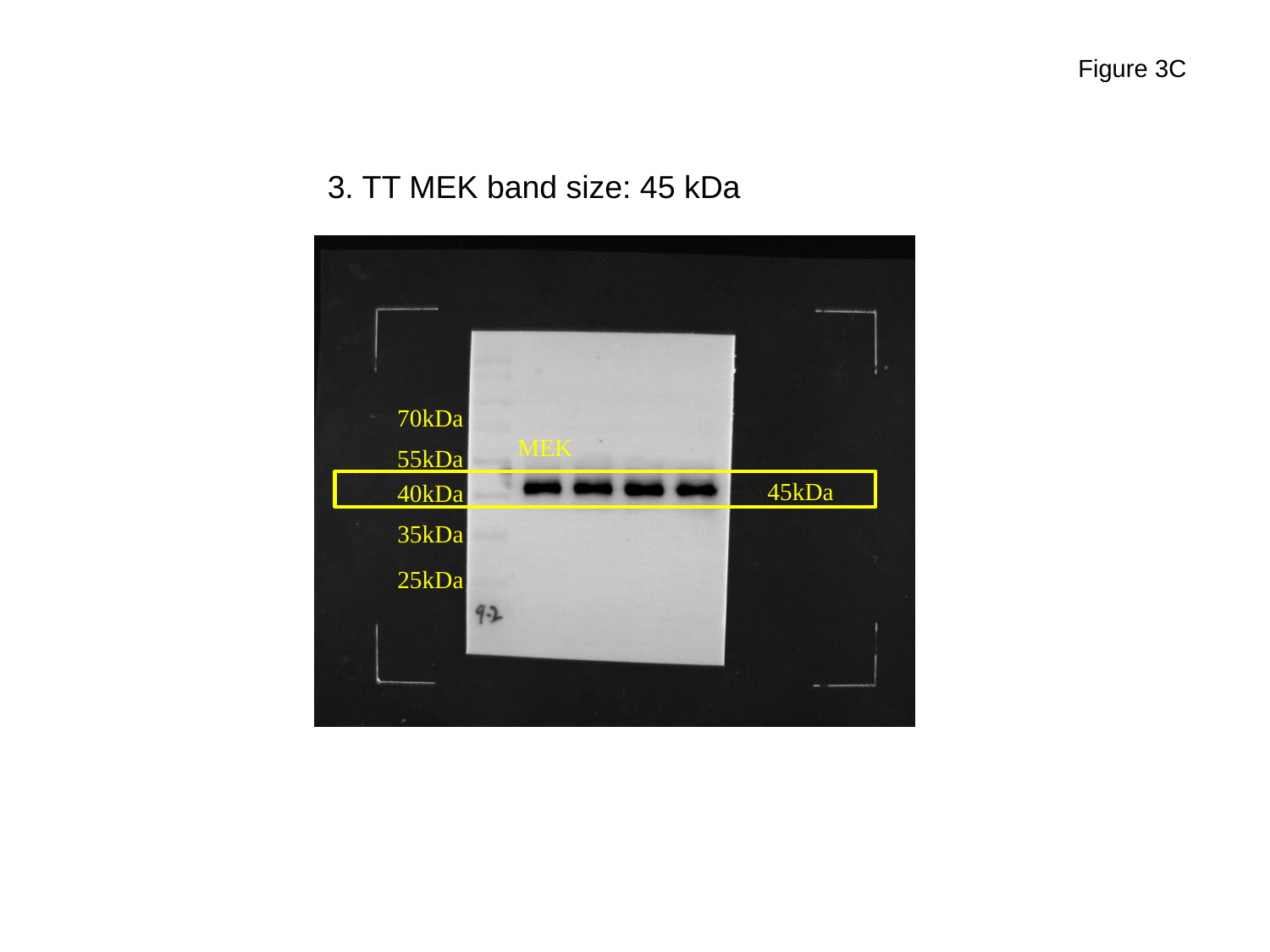

Figure 3C
3. TT MEK band size: 45 kDa
70kDa
MEK
55kDa
45kDa
40kDa
35kDa
25kDa

## Slide 26
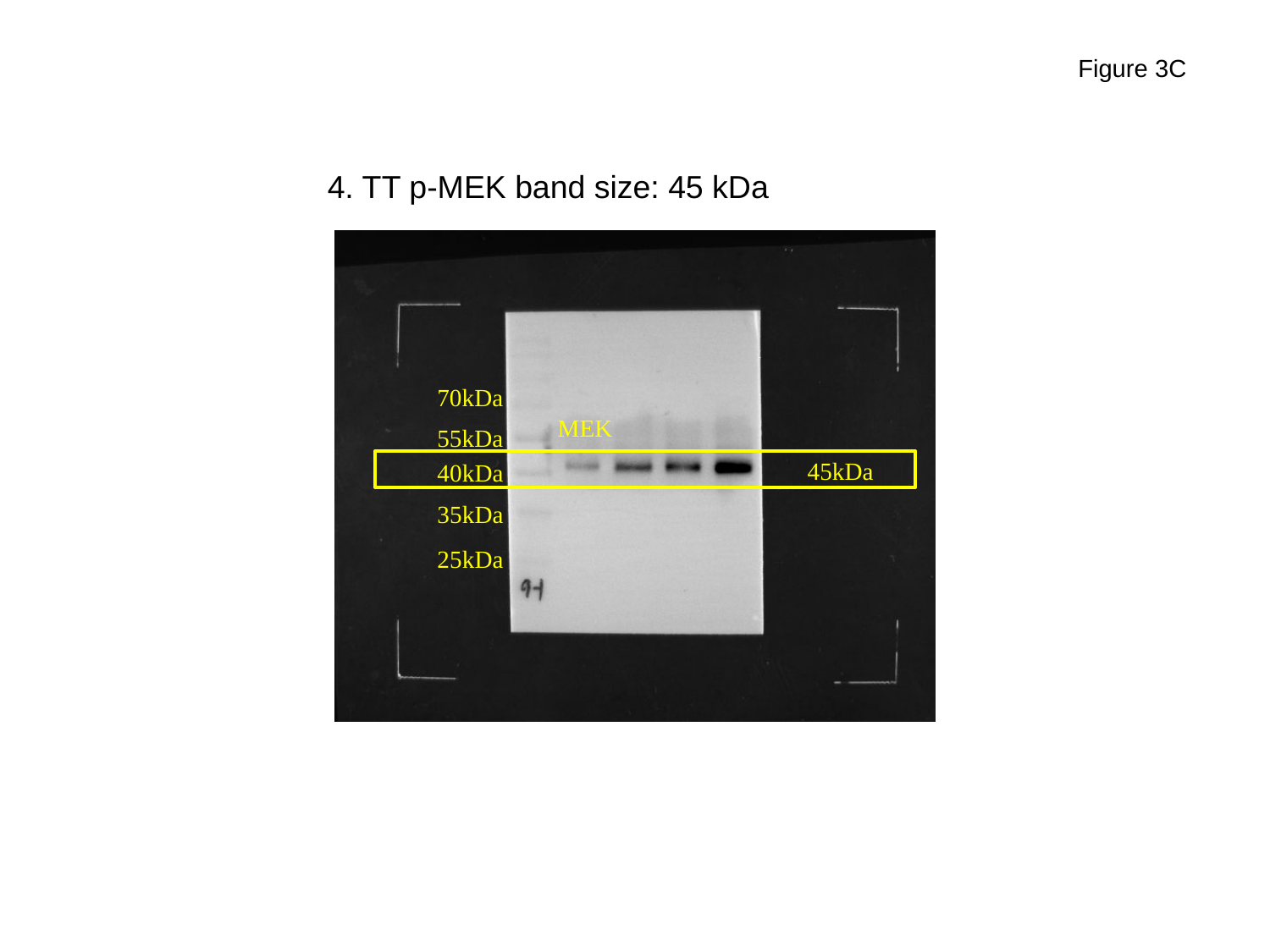

Figure 3C
4. TT p-MEK band size: 45 kDa
70kDa
MEK
55kDa
45kDa
40kDa
35kDa
25kDa

## Slide 27
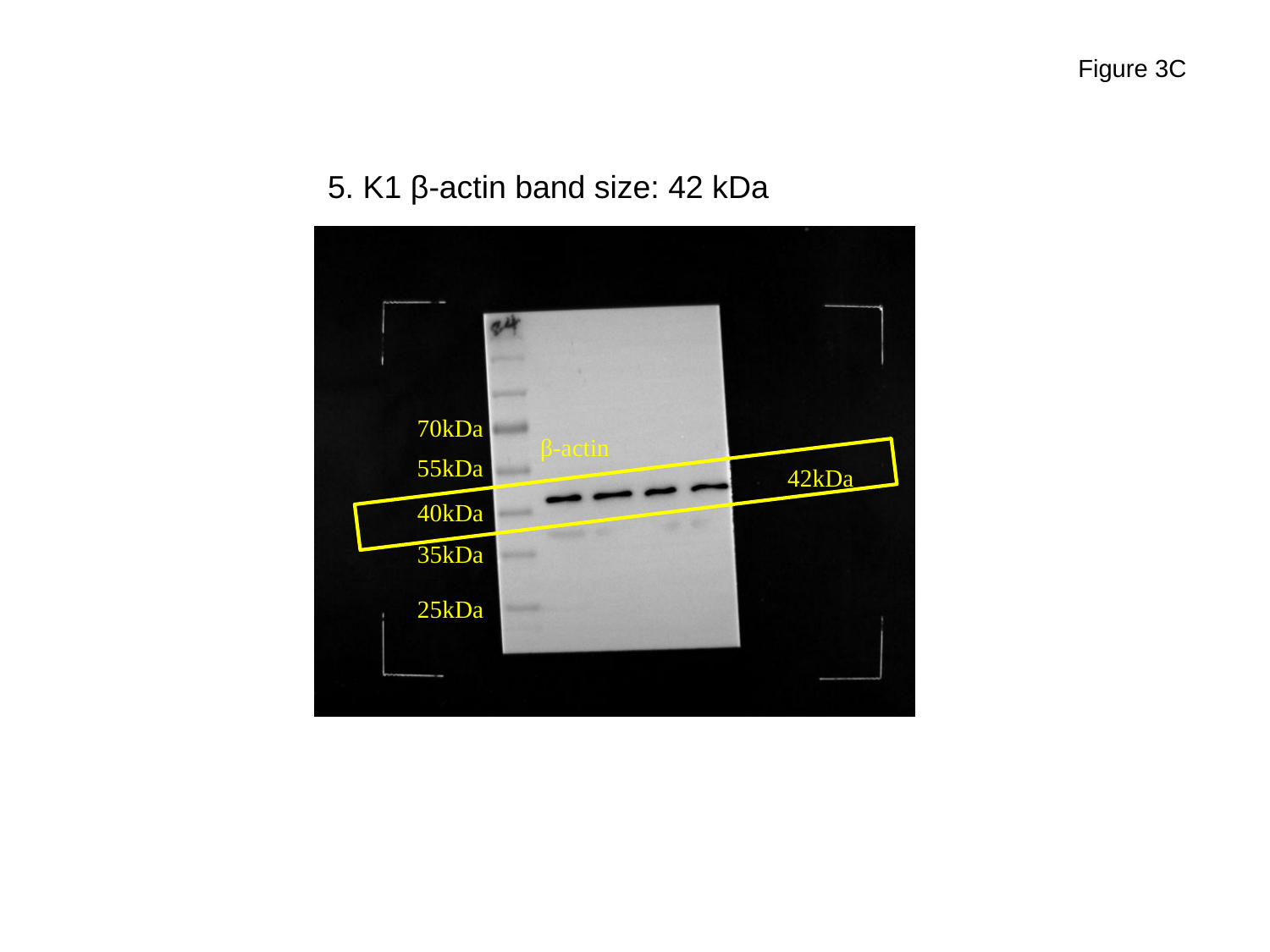

Figure 3C
5. K1 β-actin band size: 42 kDa
70kDa
β-actin
55kDa
42kDa
40kDa
35kDa
25kDa

## Slide 28
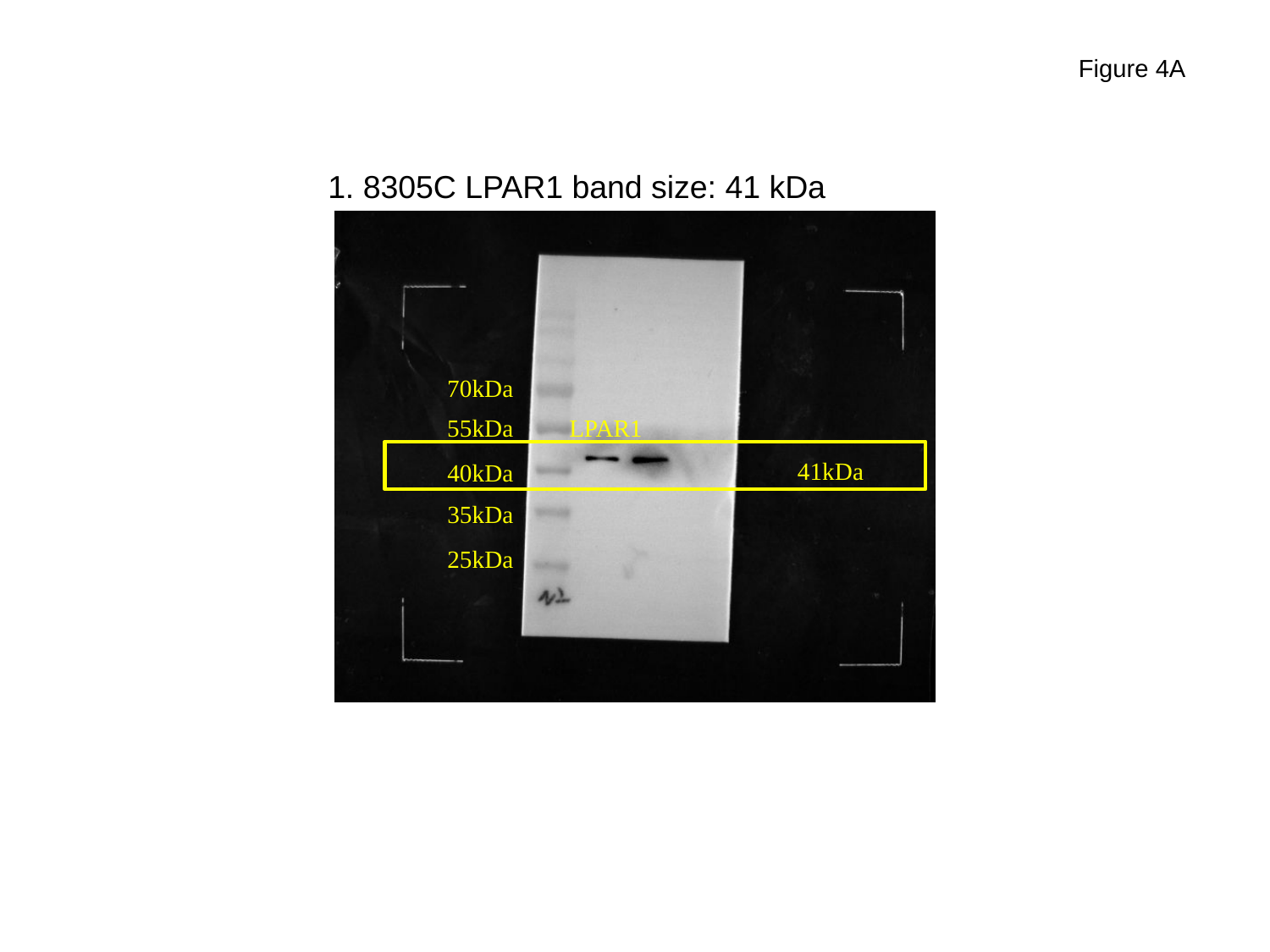

Figure 4A
1. 8305C LPAR1 band size: 41 kDa
70kDa
LPAR1
55kDa
41kDa
40kDa
35kDa
25kDa

## Slide 29
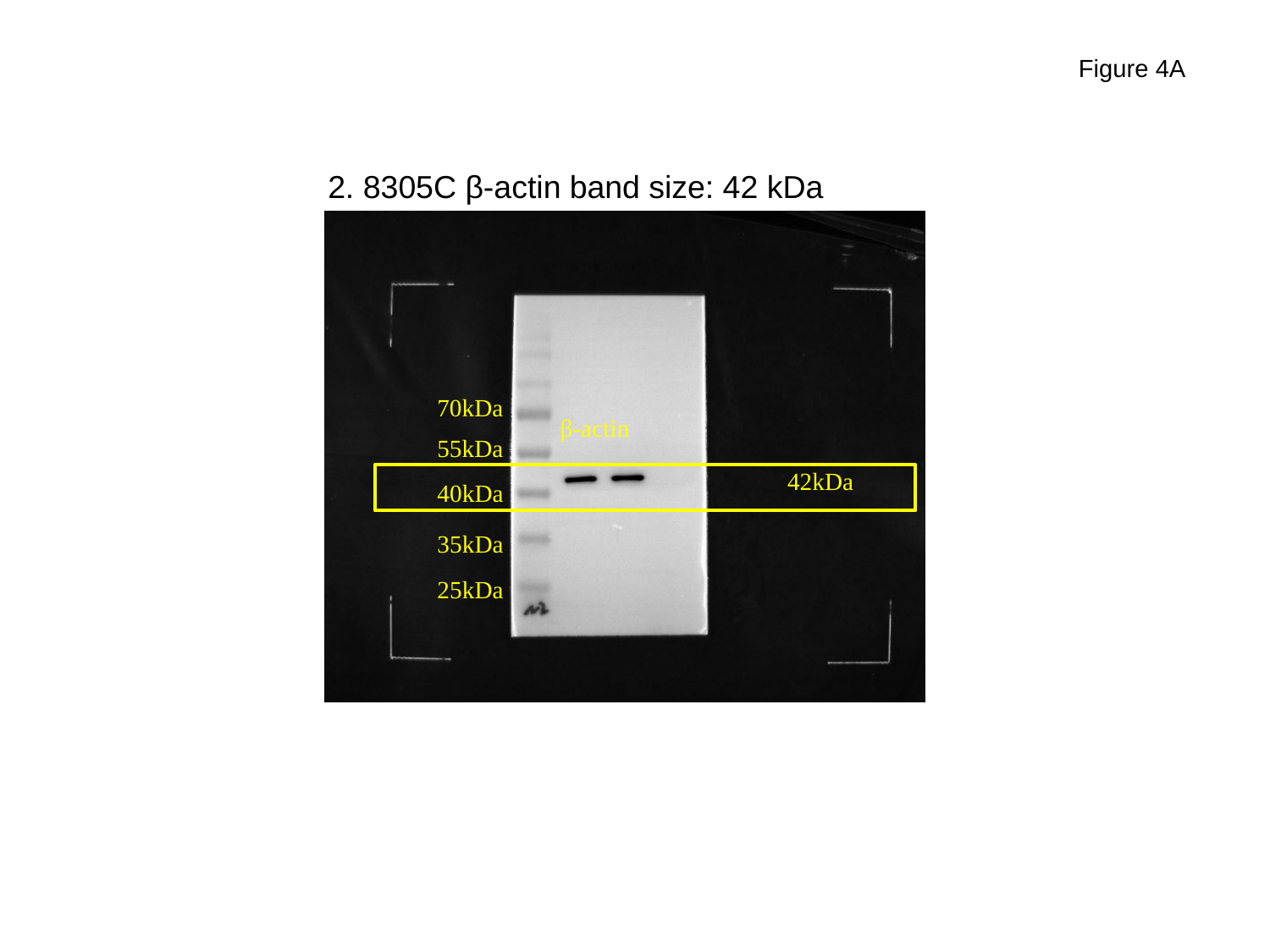

Figure 4A
2. 8305C β-actin band size: 42 kDa
70kDa
β-actin
55kDa
42kDa
40kDa
35kDa
25kDa

## Slide 30
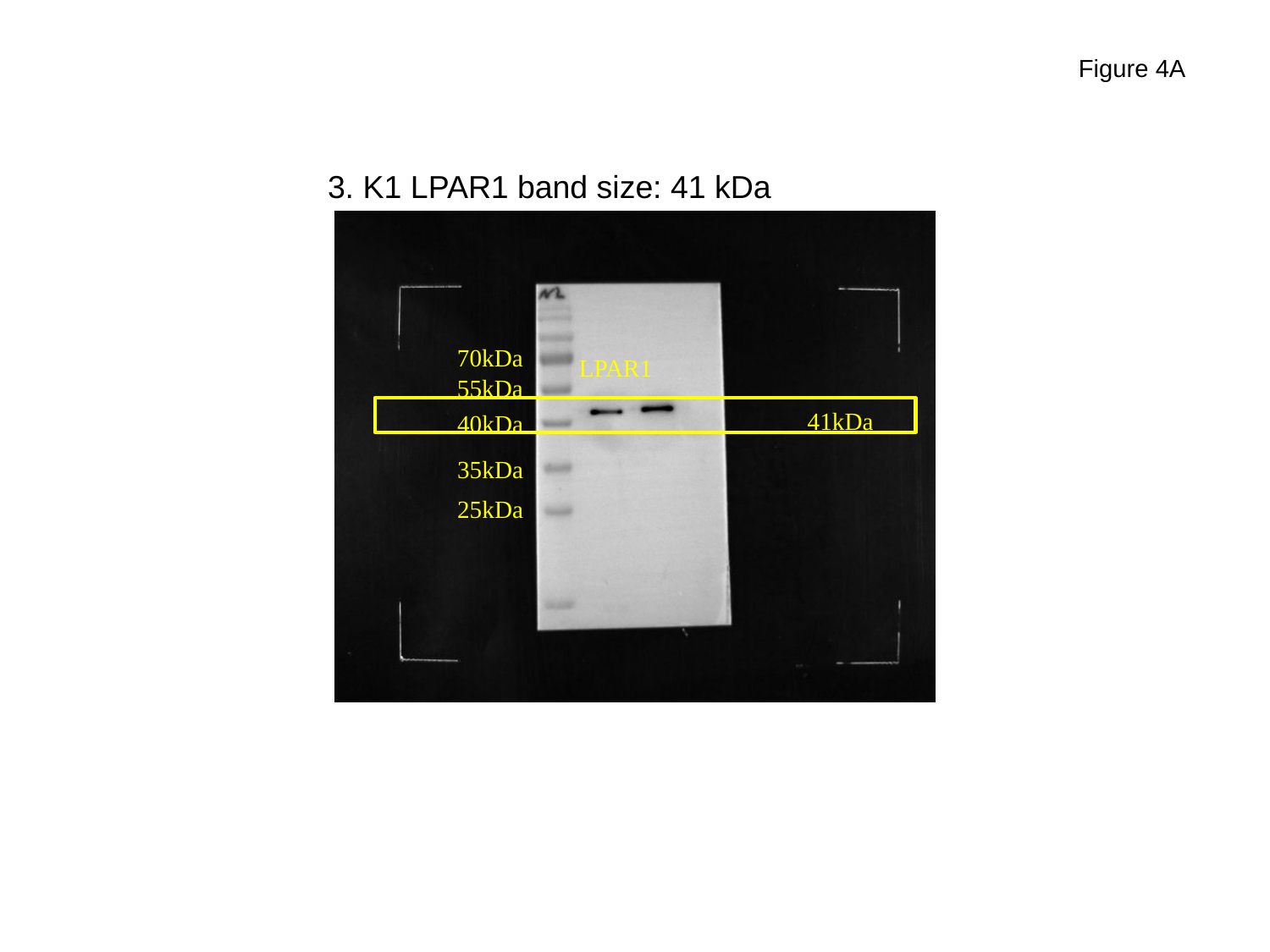

Figure 4A
3. K1 LPAR1 band size: 41 kDa
70kDa
LPAR1
55kDa
41kDa
40kDa
35kDa
25kDa

## Slide 31
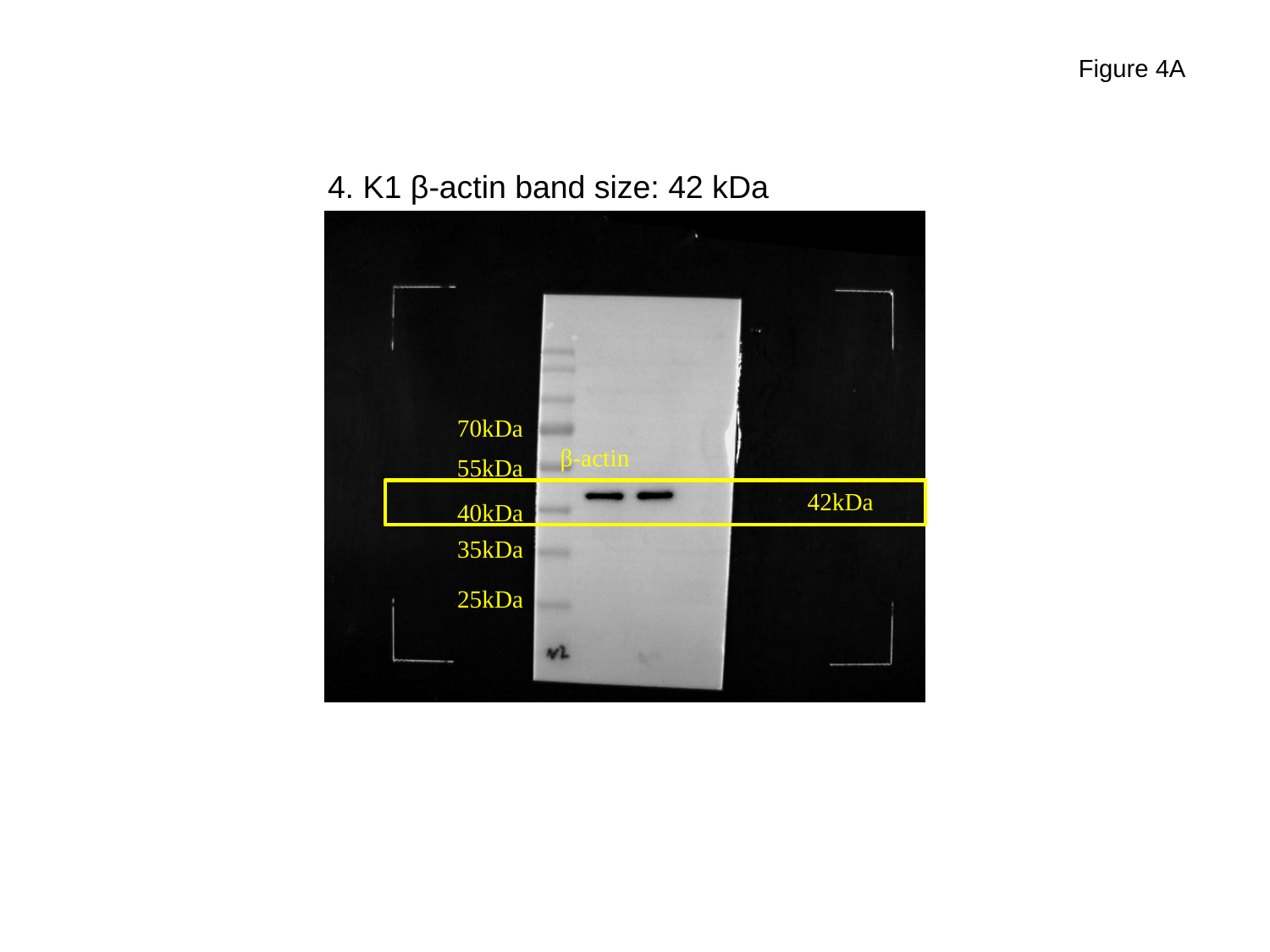

Figure 4A
4. K1 β-actin band size: 42 kDa
70kDa
β-actin
55kDa
42kDa
40kDa
35kDa
25kDa

## Slide 32
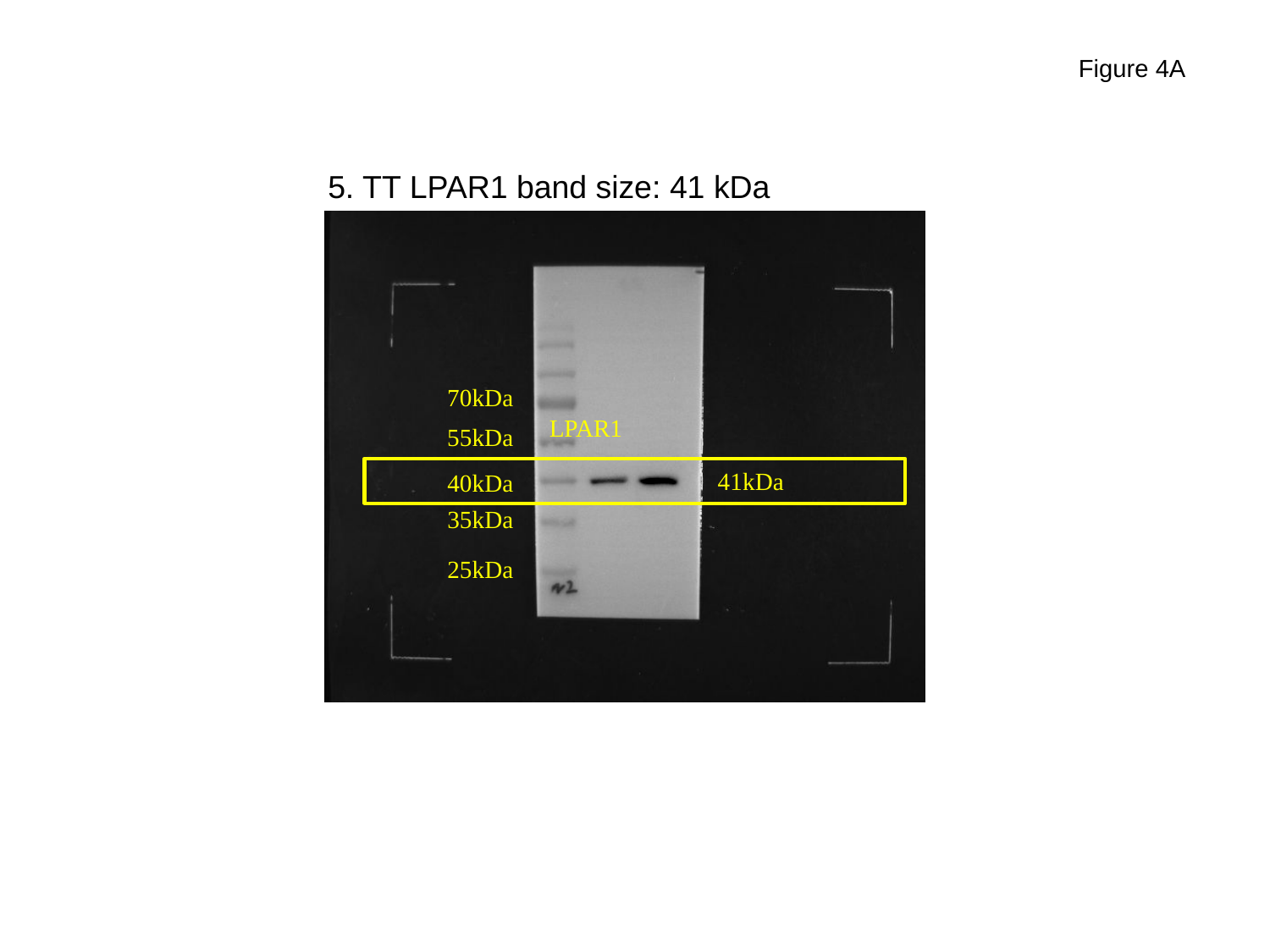

Figure 4A
5. TT LPAR1 band size: 41 kDa
70kDa
LPAR1
55kDa
41kDa
40kDa
35kDa
25kDa

## Slide 33
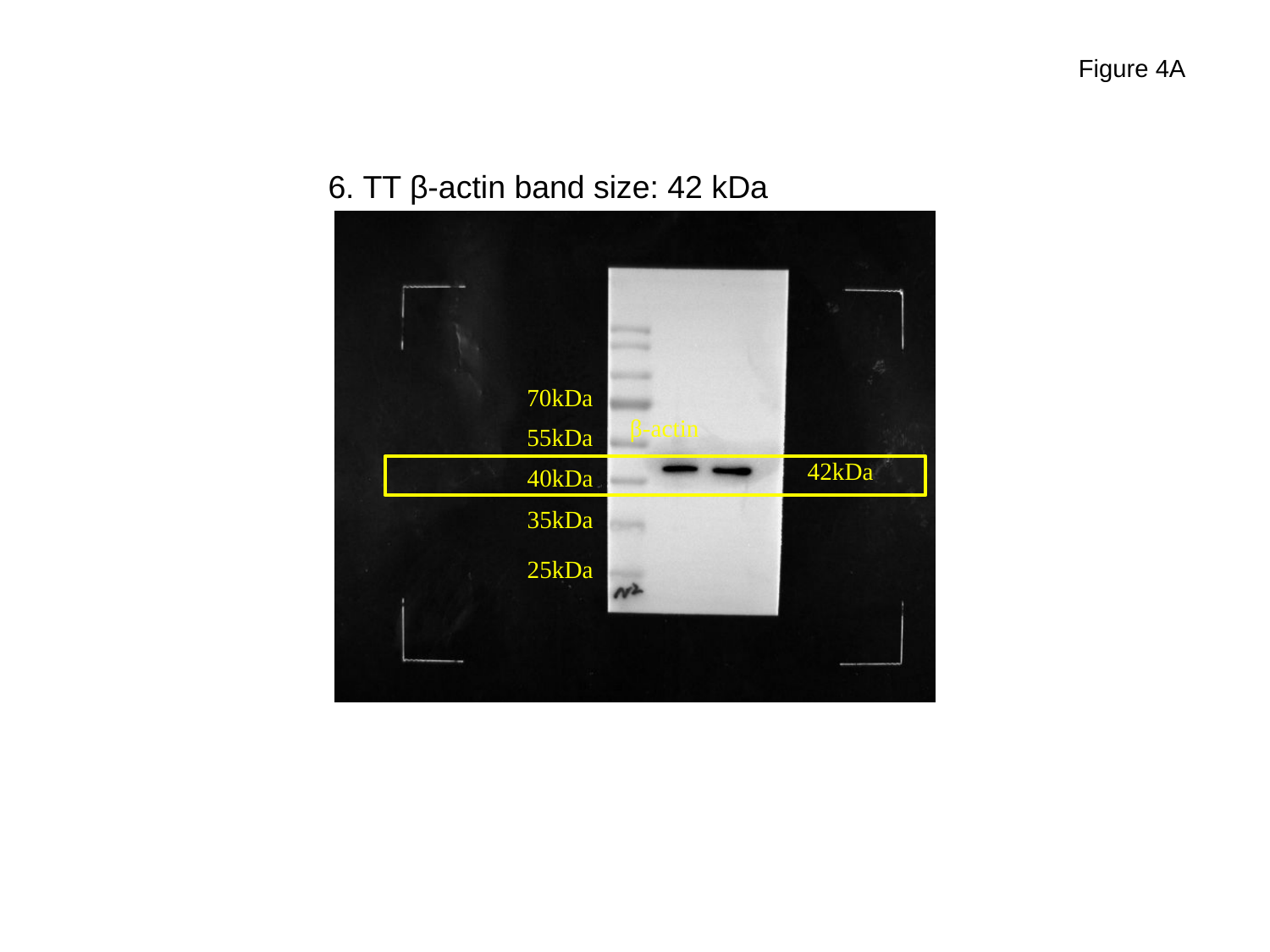

Figure 4A
6. TT β-actin band size: 42 kDa
70kDa
β-actin
55kDa
42kDa
40kDa
35kDa
25kDa

## Slide 34
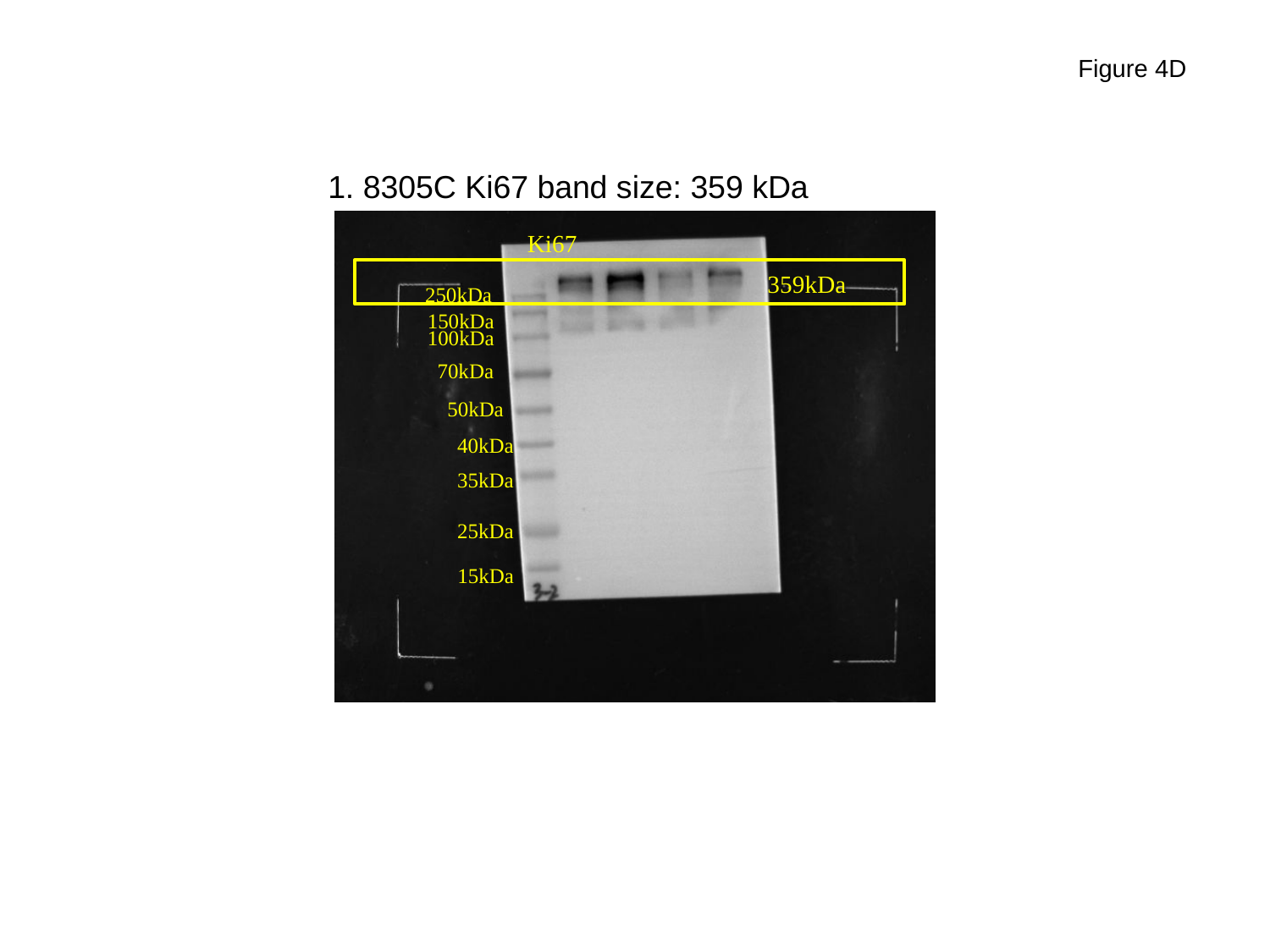

Figure 4D
1. 8305C Ki67 band size: 359 kDa
Ki67
359kDa
250kDa
150kDa
100kDa
70kDa
50kDa
40kDa
35kDa
25kDa
15kDa

## Slide 35
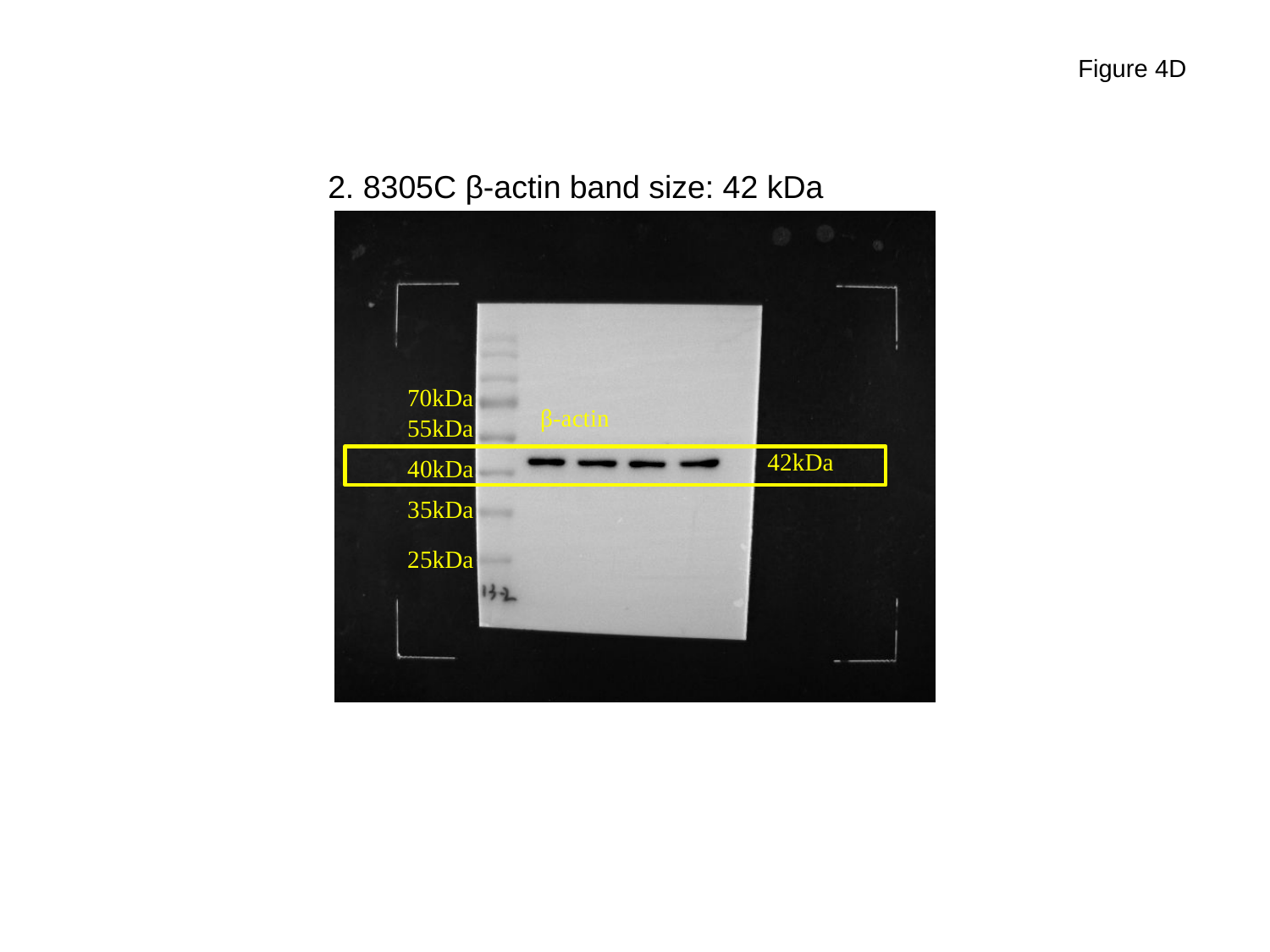

Figure 4D
2. 8305C β-actin band size: 42 kDa
70kDa
β-actin
55kDa
42kDa
40kDa
35kDa
25kDa

## Slide 36
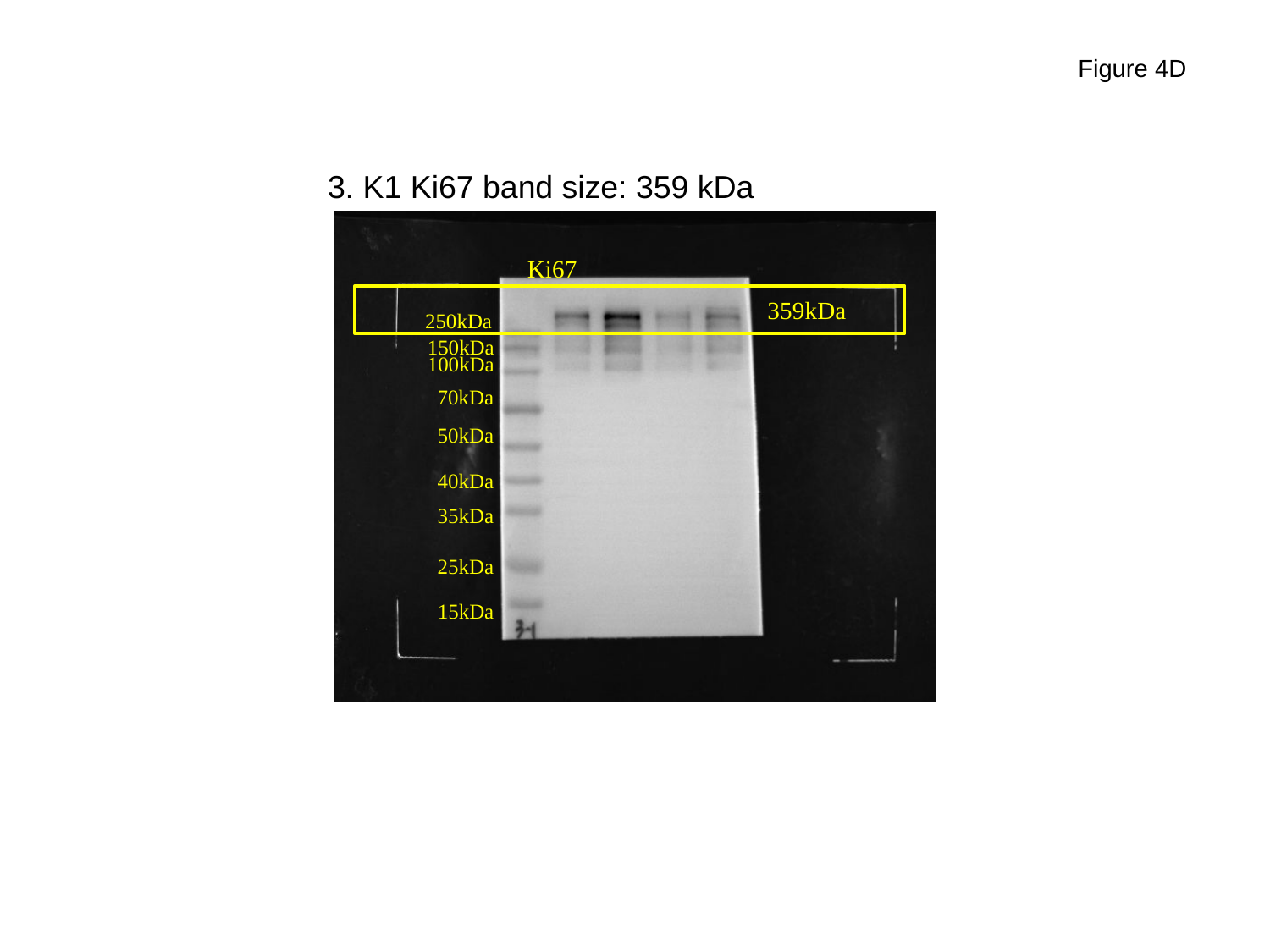

Figure 4D
3. K1 Ki67 band size: 359 kDa
Ki67
359kDa
250kDa
150kDa
100kDa
70kDa
50kDa
40kDa
35kDa
25kDa
15kDa

## Slide 37
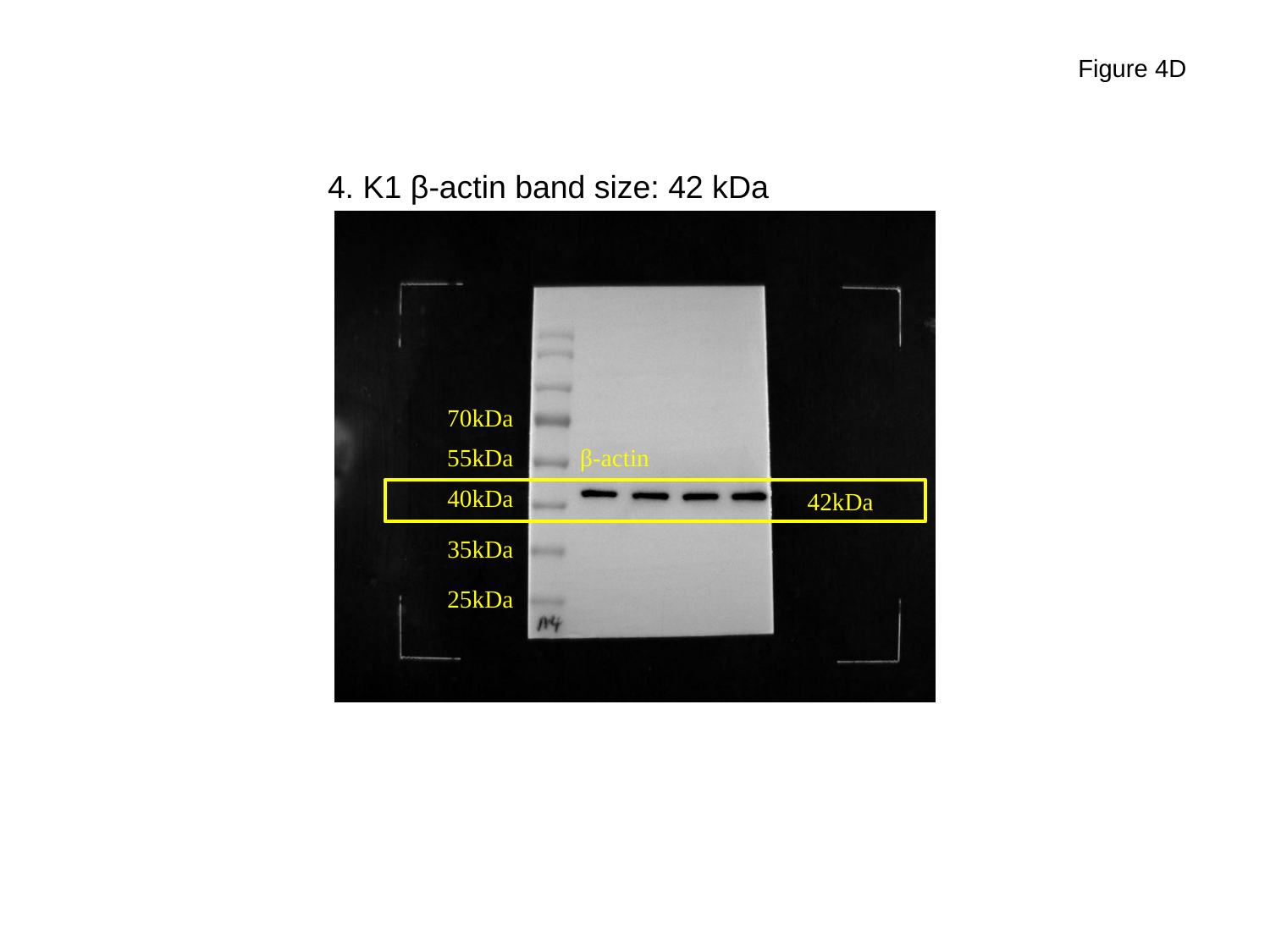

Figure 4D
4. K1 β-actin band size: 42 kDa
70kDa
55kDa
β-actin
40kDa
42kDa
35kDa
25kDa

## Slide 38
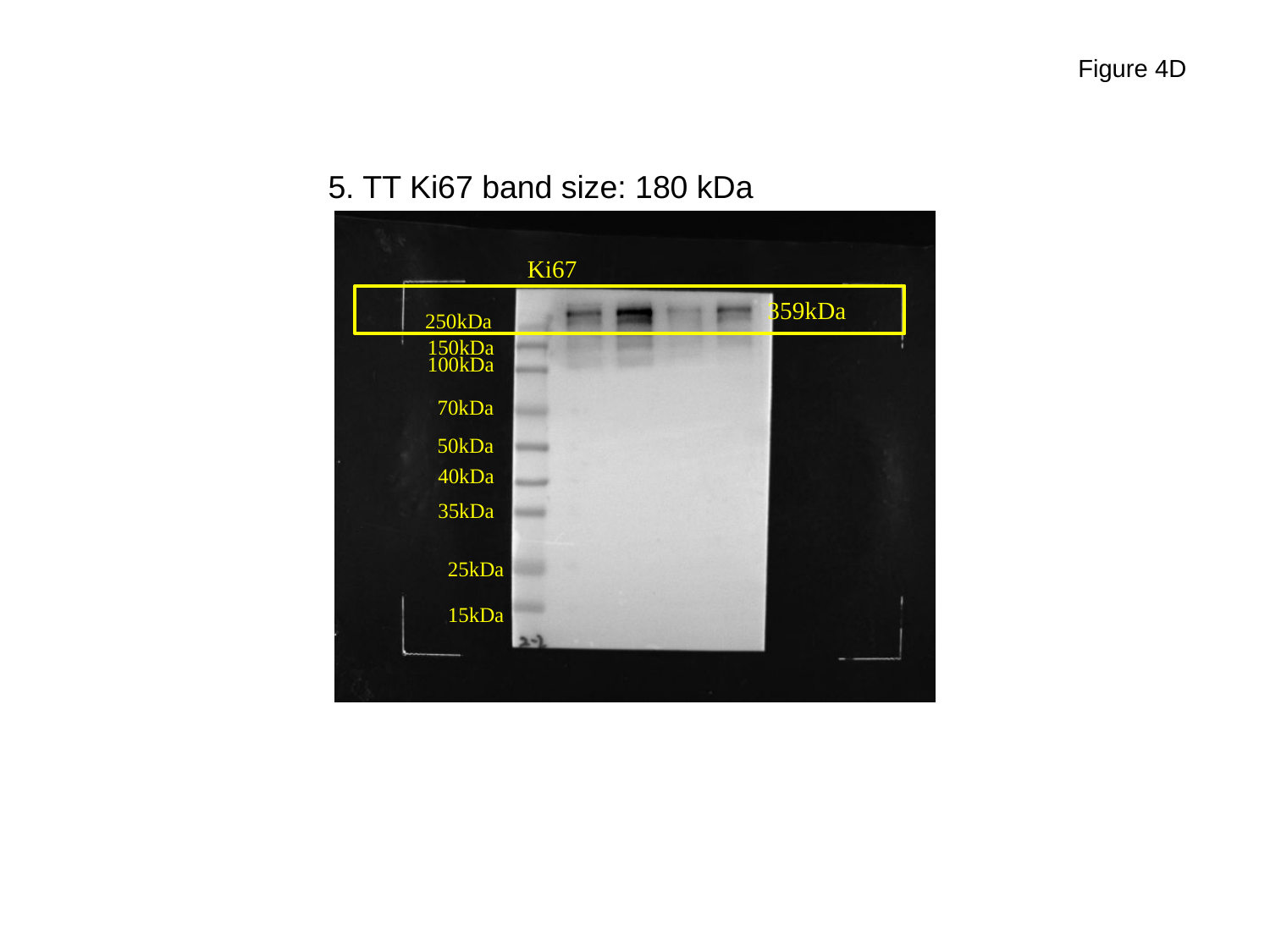

Figure 4D
5. TT Ki67 band size: 180 kDa
Ki67
359kDa
250kDa
150kDa
100kDa
70kDa
50kDa
40kDa
35kDa
25kDa
15kDa

## Slide 39
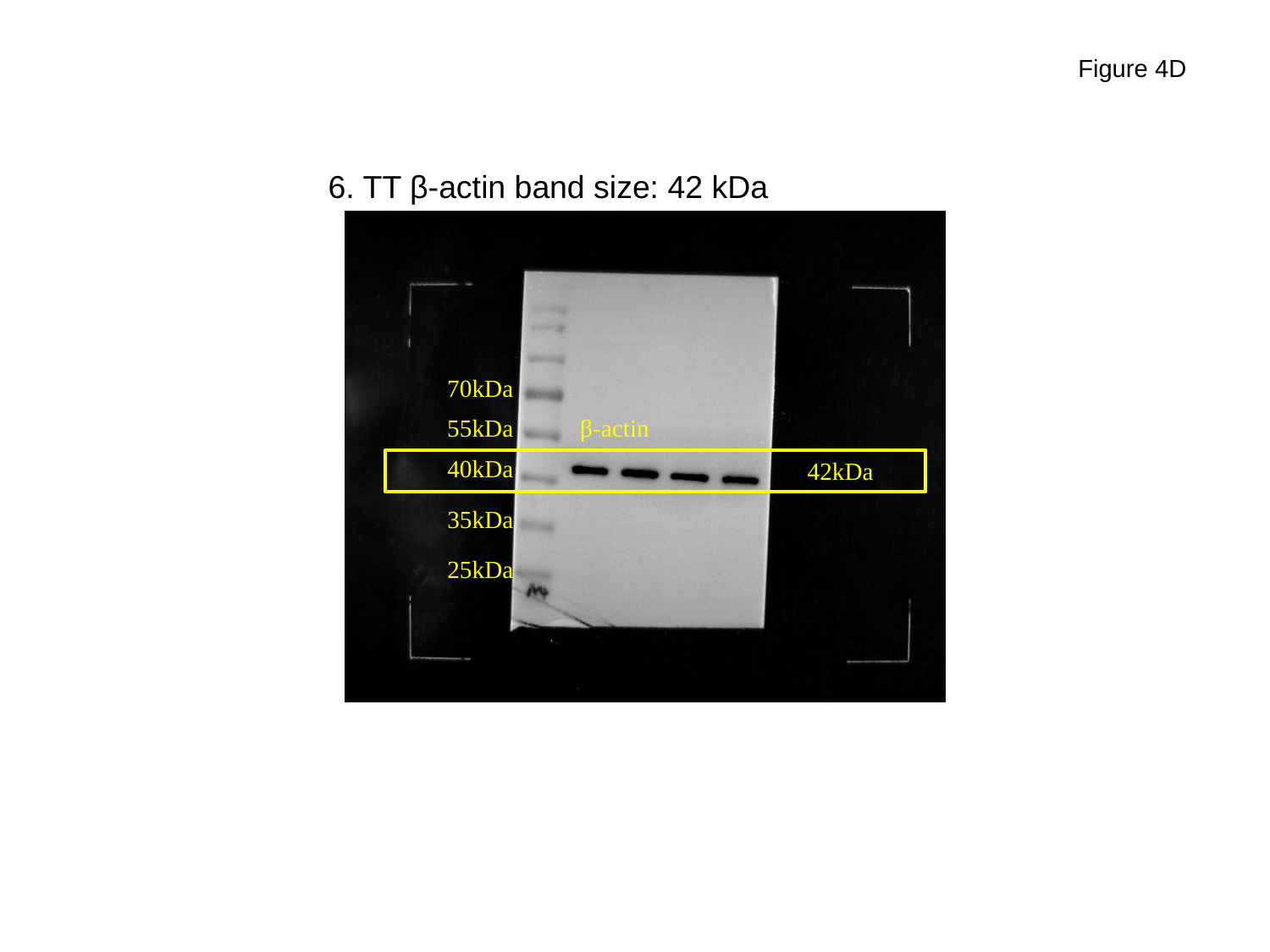

Figure 4D
6. TT β-actin band size: 42 kDa
70kDa
55kDa
β-actin
40kDa
42kDa
35kDa
25kDa

## Slide 40
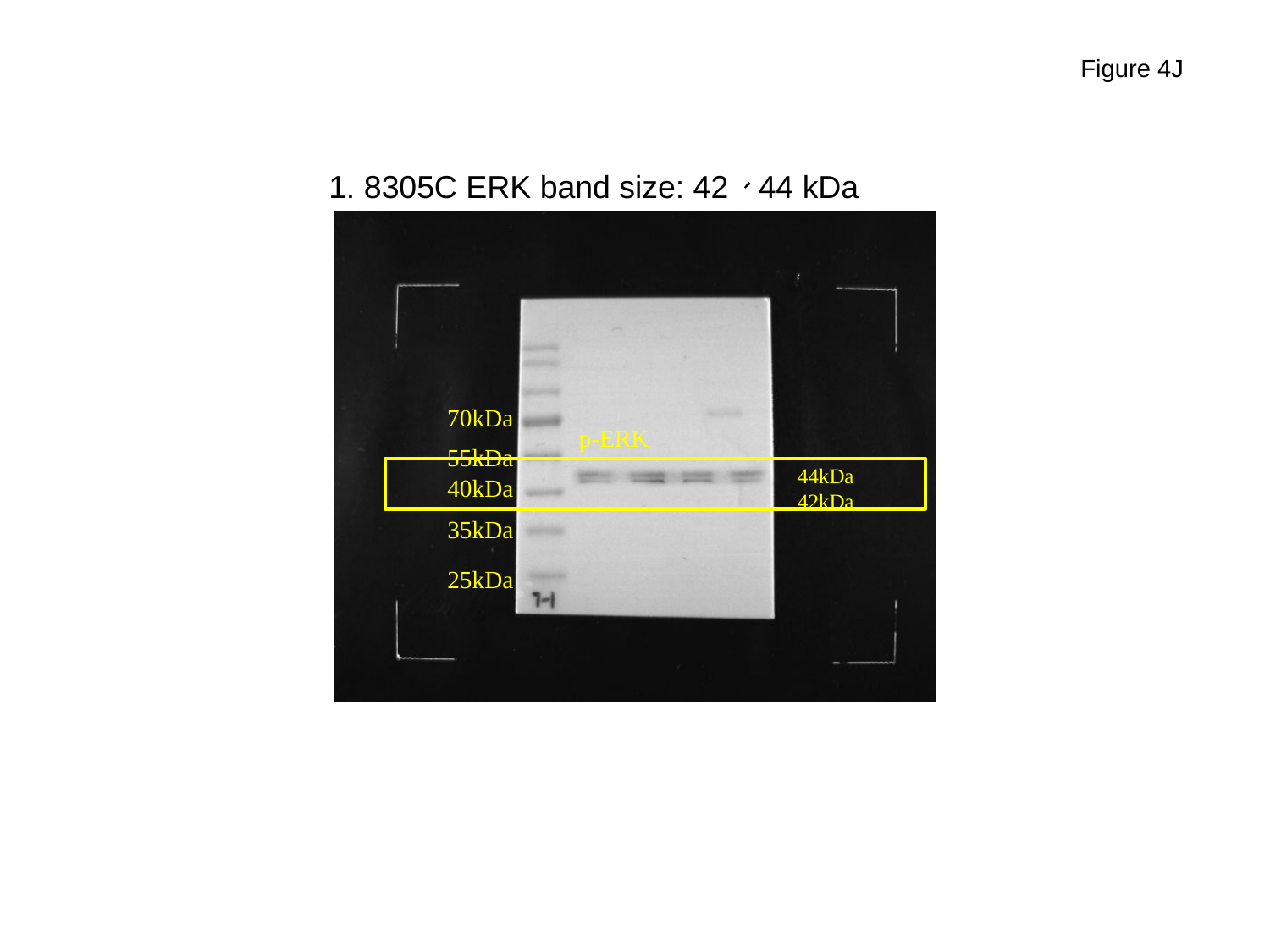

Figure 4J
1. 8305C ERK band size: 42、44 kDa
70kDa
p-ERK
55kDa
44kDa
42kDa
40kDa
35kDa
25kDa

## Slide 41
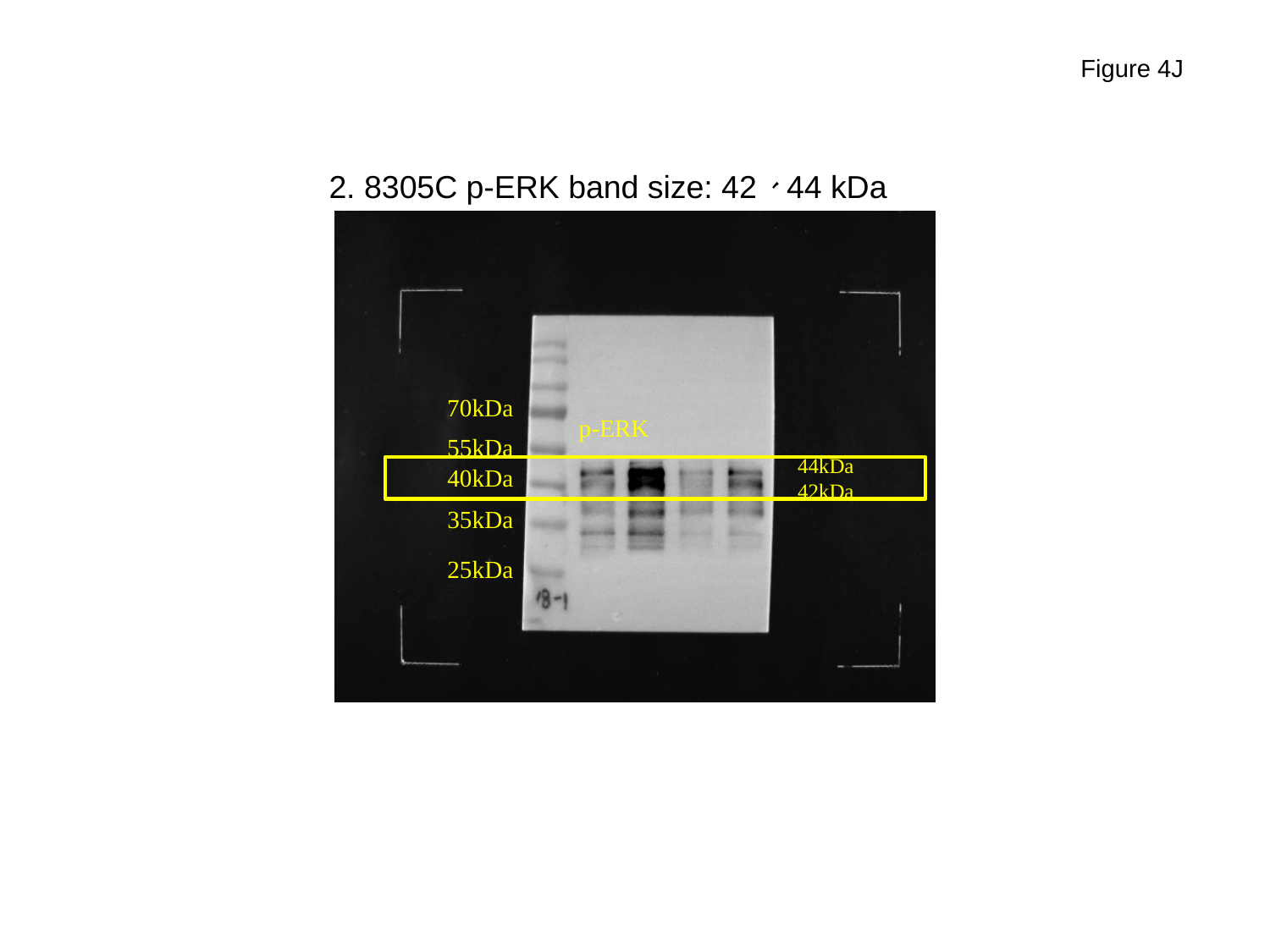

Figure 4J
2. 8305C p-ERK band size: 42、44 kDa
70kDa
p-ERK
55kDa
44kDa
42kDa
40kDa
35kDa
25kDa

## Slide 42
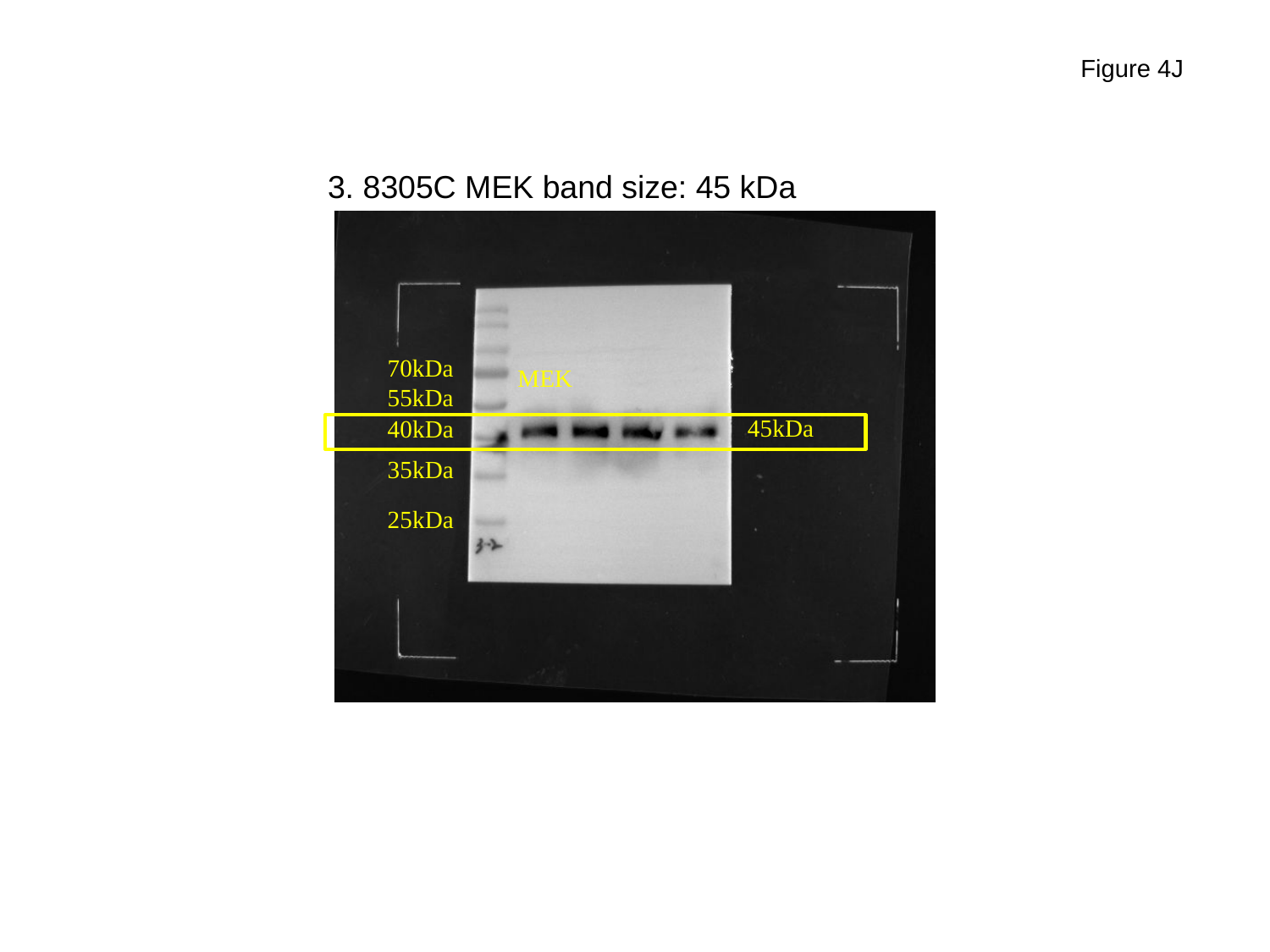

Figure 4J
3. 8305C MEK band size: 45 kDa
70kDa
MEK
55kDa
45kDa
40kDa
35kDa
25kDa

## Slide 43
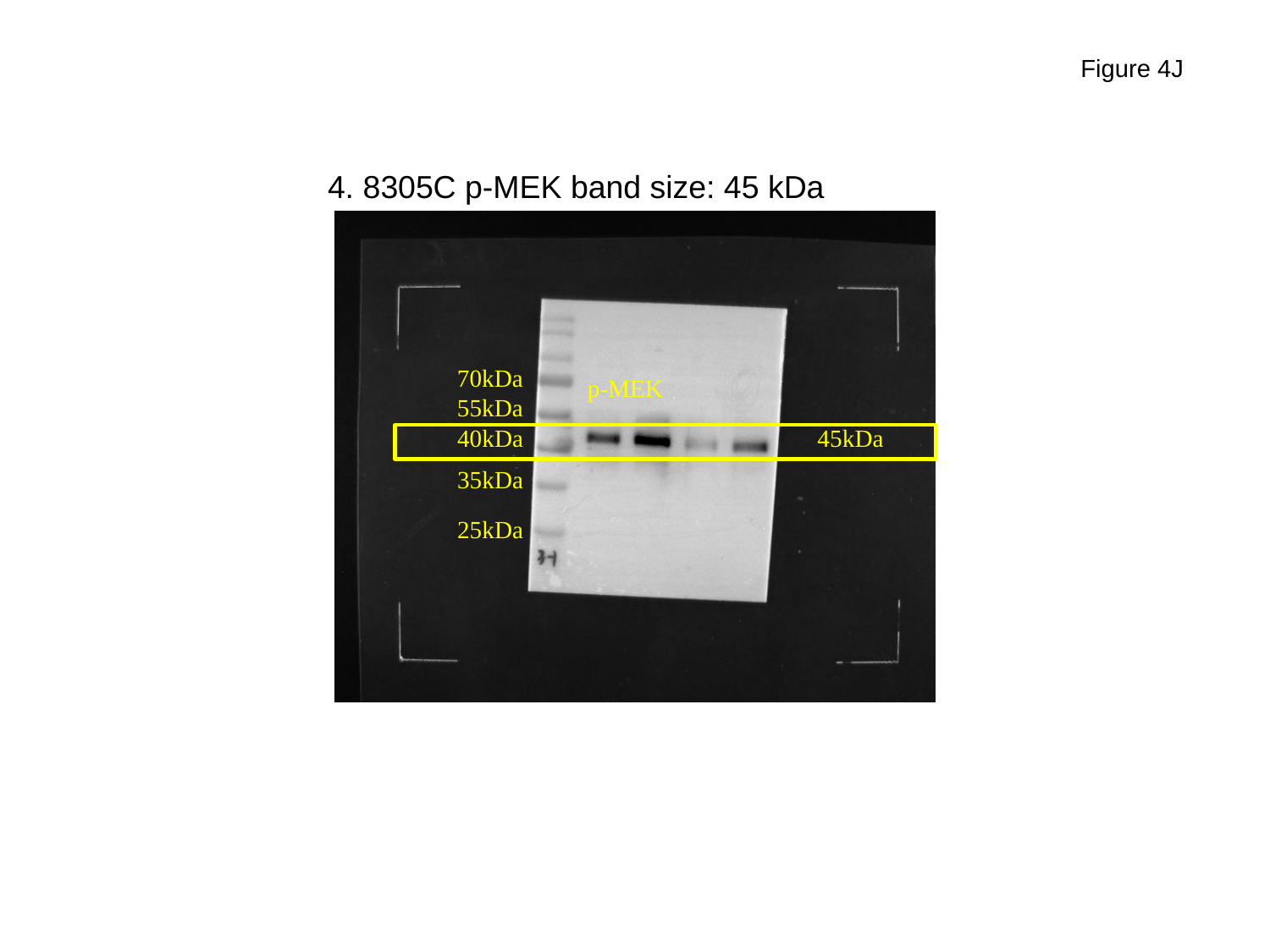

Figure 4J
4. 8305C p-MEK band size: 45 kDa
70kDa
p-MEK
55kDa
45kDa
40kDa
35kDa
25kDa

## Slide 44
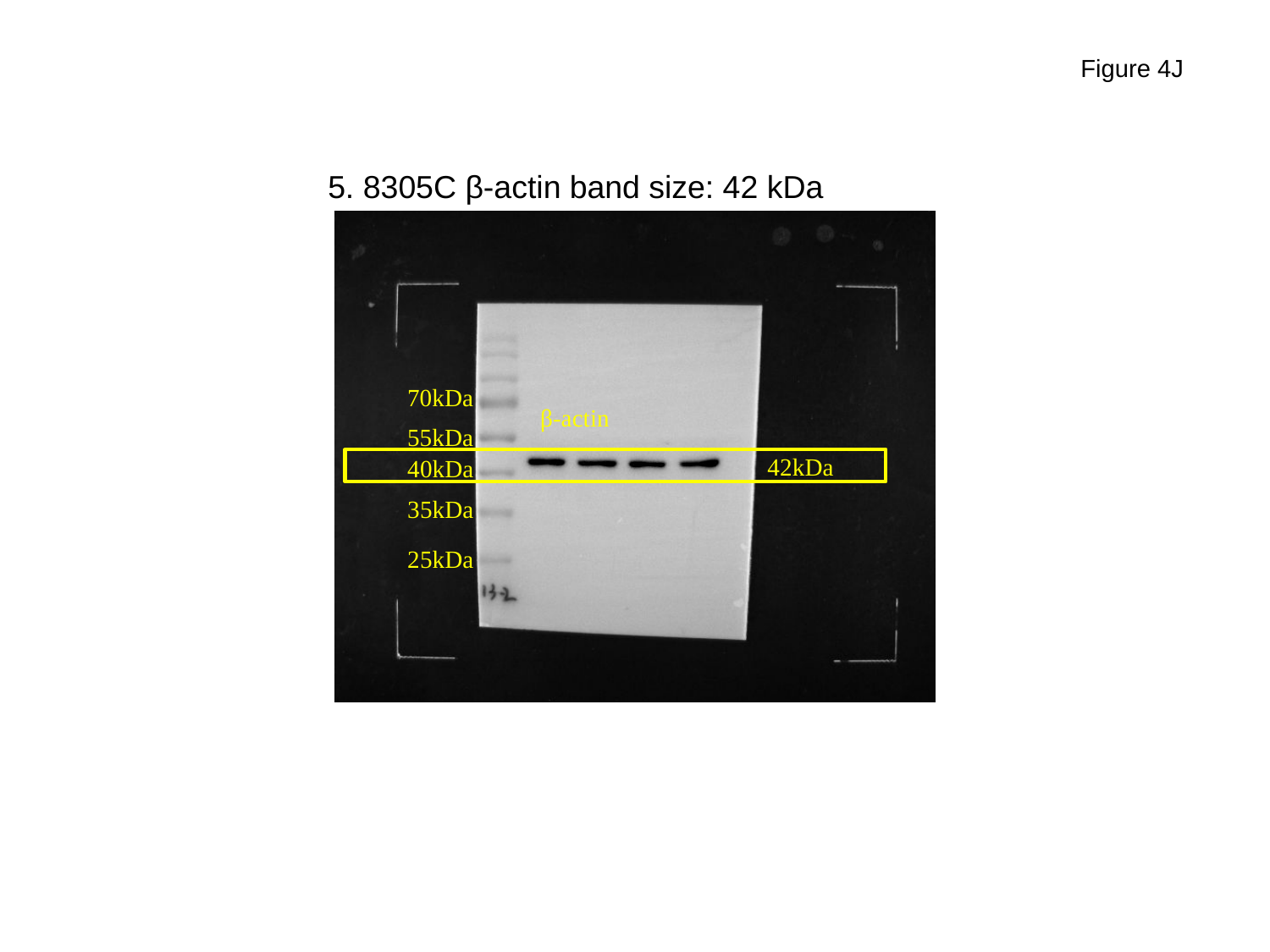

Figure 4J
5. 8305C β-actin band size: 42 kDa
70kDa
β-actin
55kDa
42kDa
40kDa
35kDa
25kDa

## Slide 45
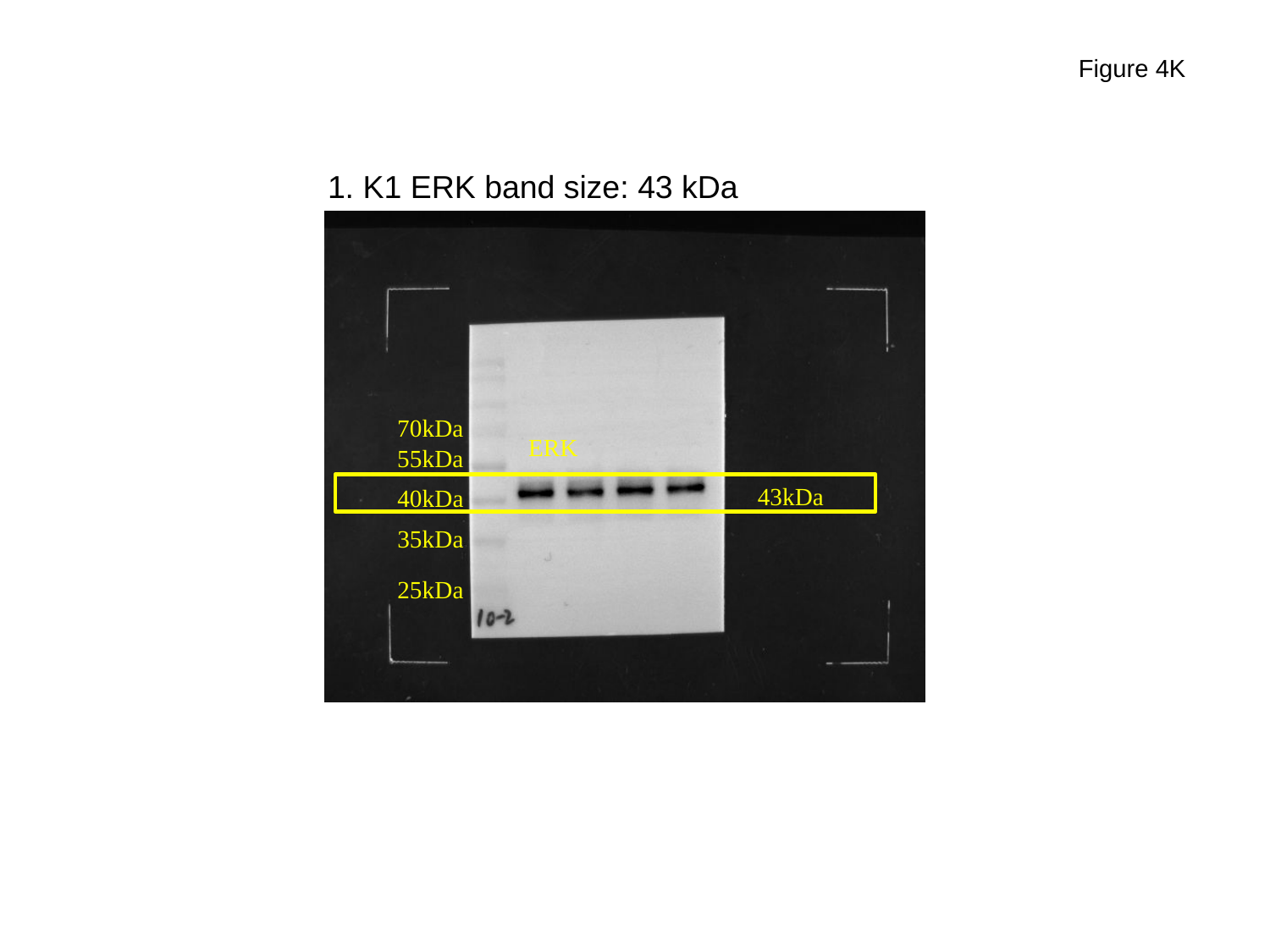

Figure 4K
1. K1 ERK band size: 43 kDa
70kDa
ERK
55kDa
43kDa
40kDa
35kDa
25kDa

## Slide 46
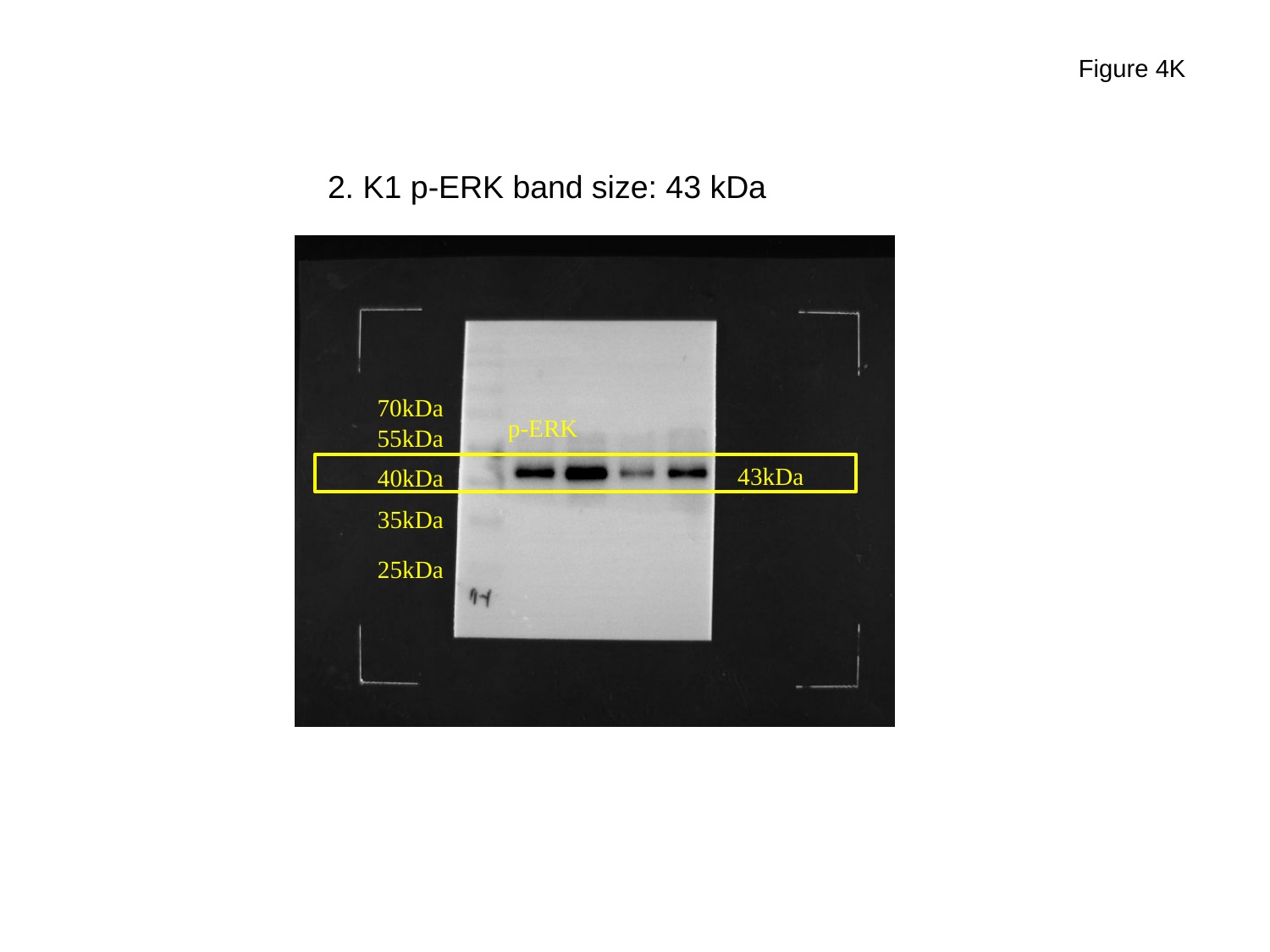

Figure 4K
2. K1 p-ERK band size: 43 kDa
70kDa
p-ERK
55kDa
43kDa
40kDa
35kDa
25kDa

## Slide 47
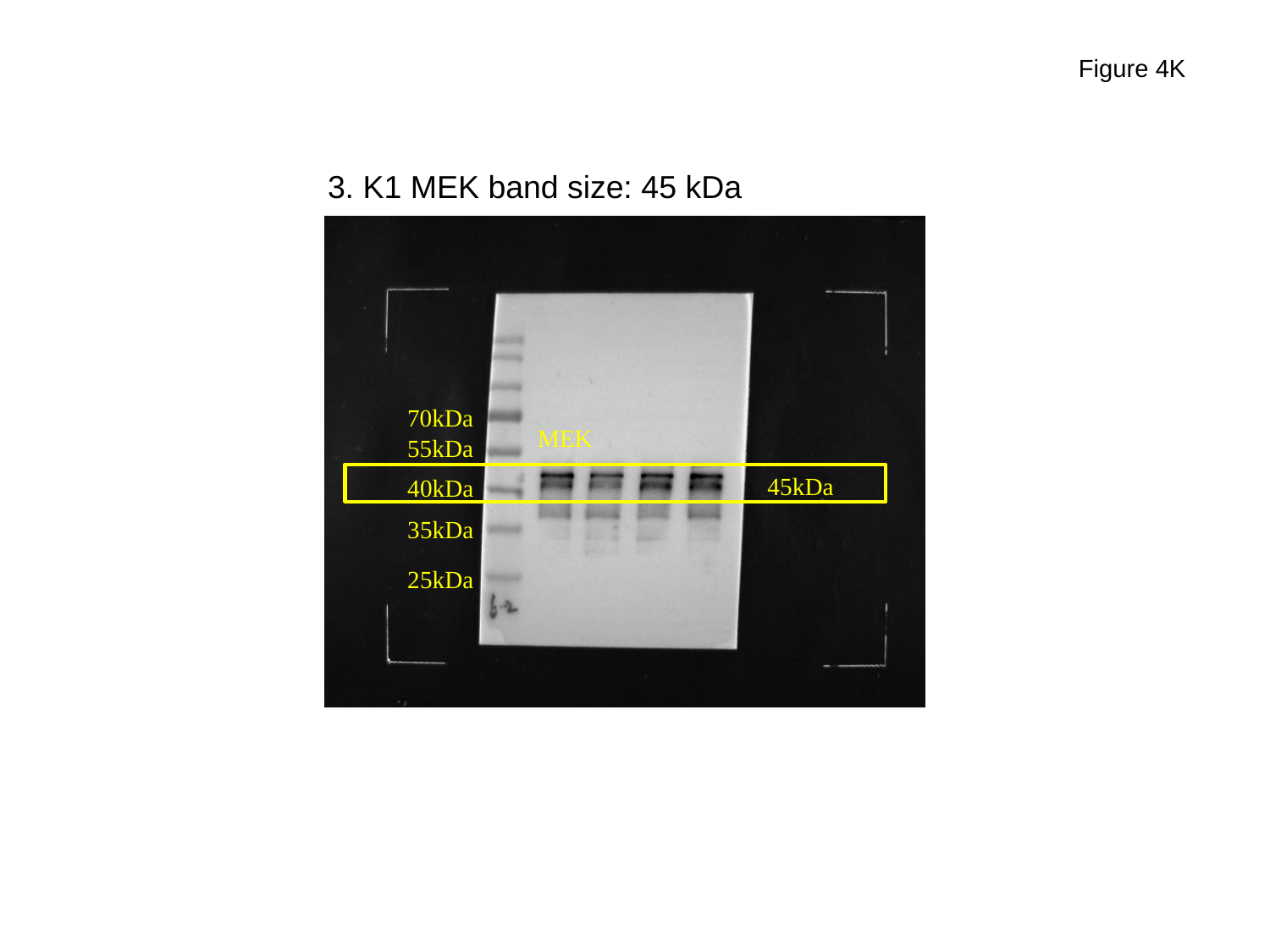

Figure 4K
3. K1 MEK band size: 45 kDa
70kDa
MEK
55kDa
45kDa
40kDa
35kDa
25kDa

## Slide 48
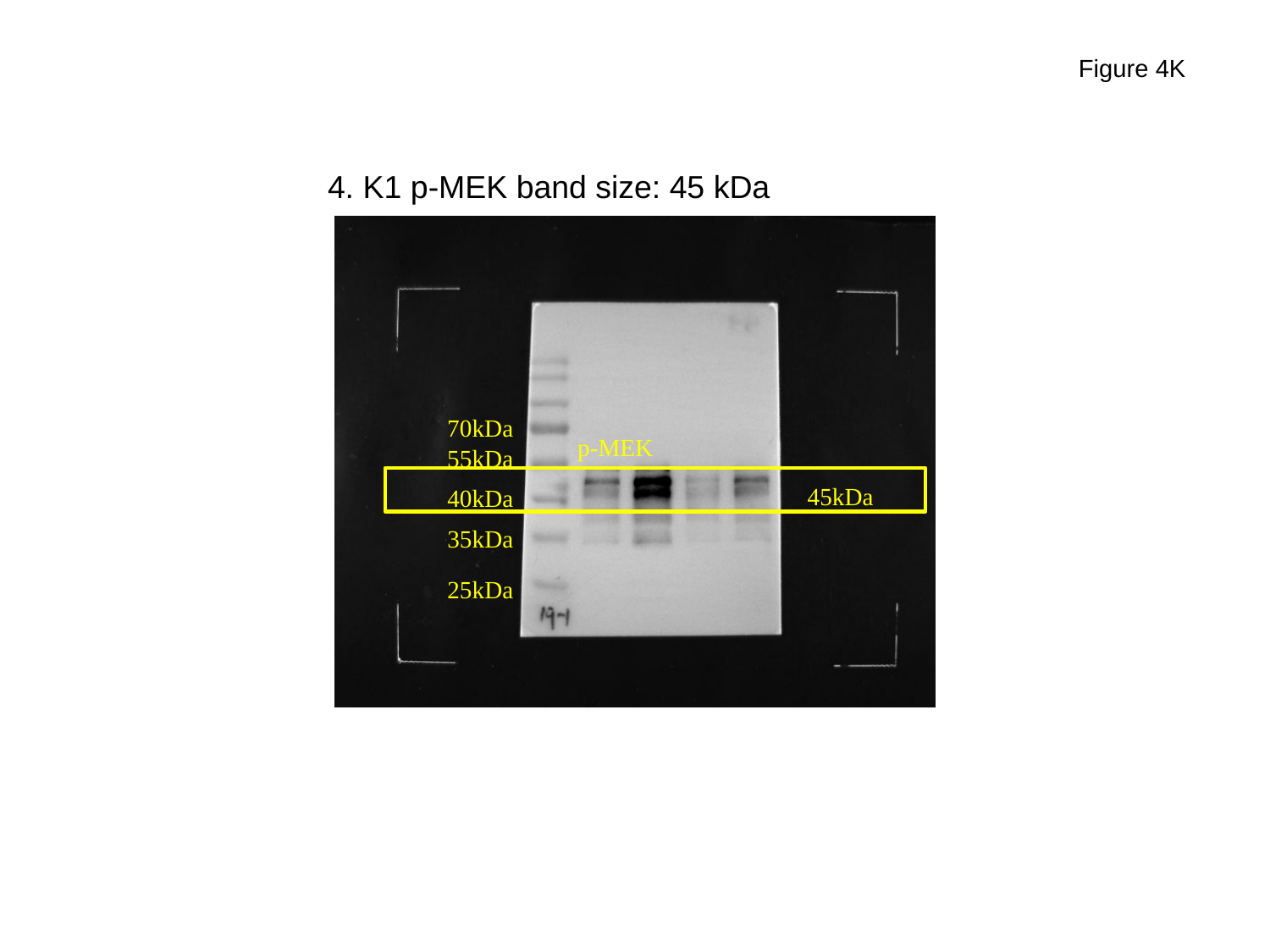

Figure 4K
4. K1 p-MEK band size: 45 kDa
70kDa
p-MEK
55kDa
45kDa
40kDa
35kDa
25kDa

## Slide 49
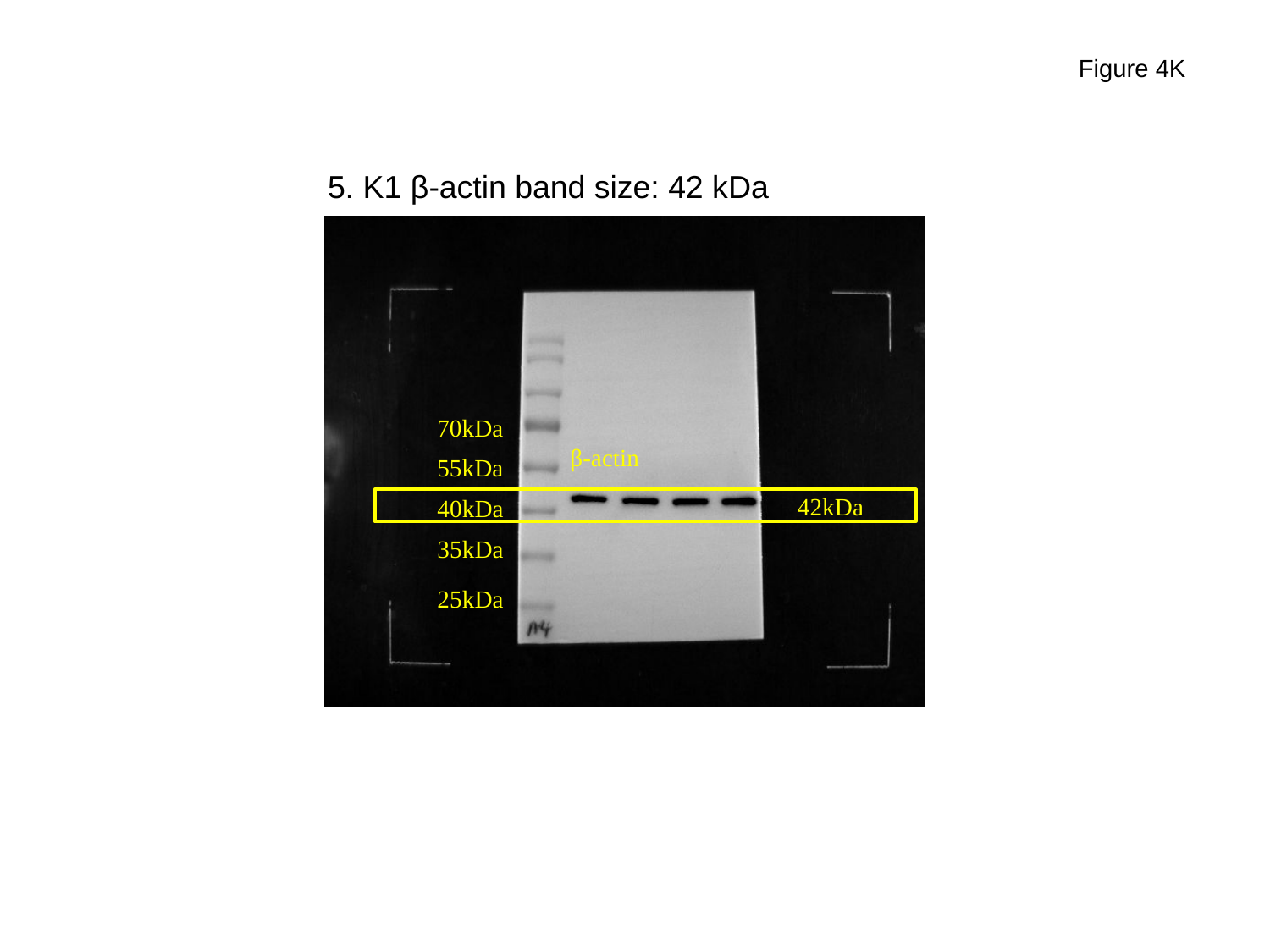

Figure 4K
5. K1 β-actin band size: 42 kDa
70kDa
β-actin
55kDa
42kDa
40kDa
35kDa
25kDa

## Slide 50
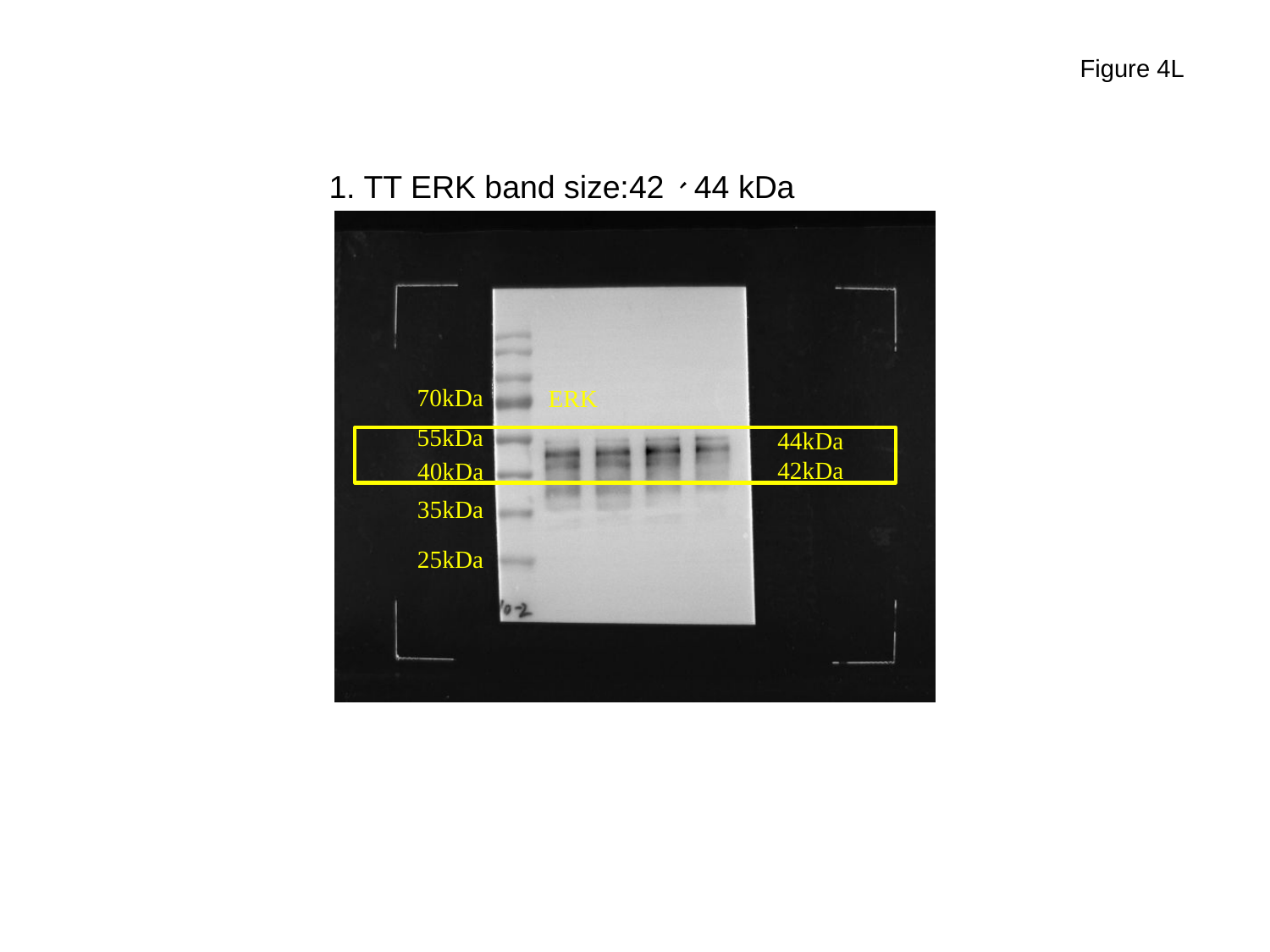

Figure 4L
1. TT ERK band size:42、44 kDa
70kDa
ERK
55kDa
44kDa
42kDa
40kDa
35kDa
25kDa

## Slide 51
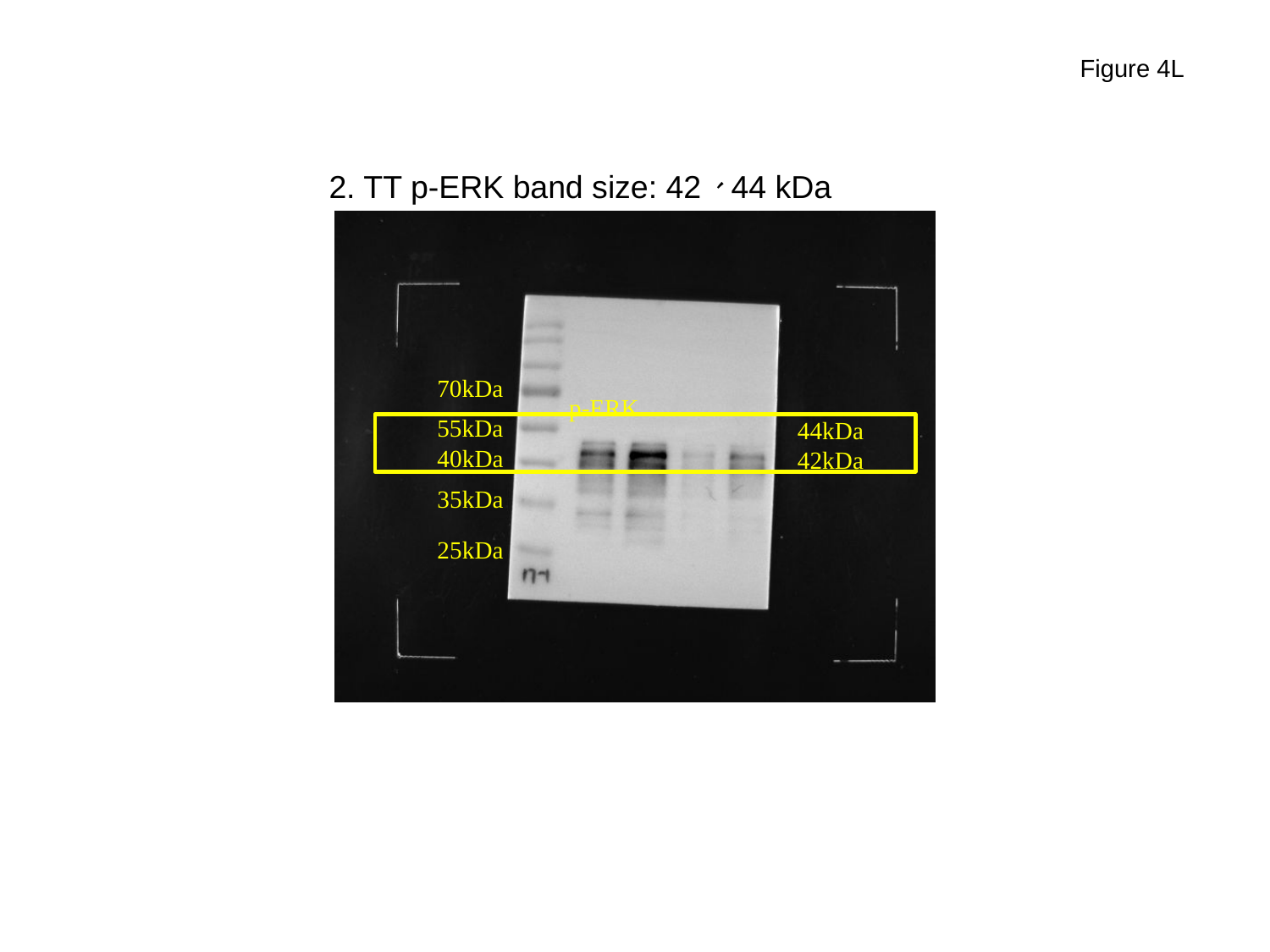

Figure 4L
2. TT p-ERK band size: 42、44 kDa
70kDa
p-ERK
55kDa
44kDa
42kDa
40kDa
35kDa
25kDa

## Slide 52
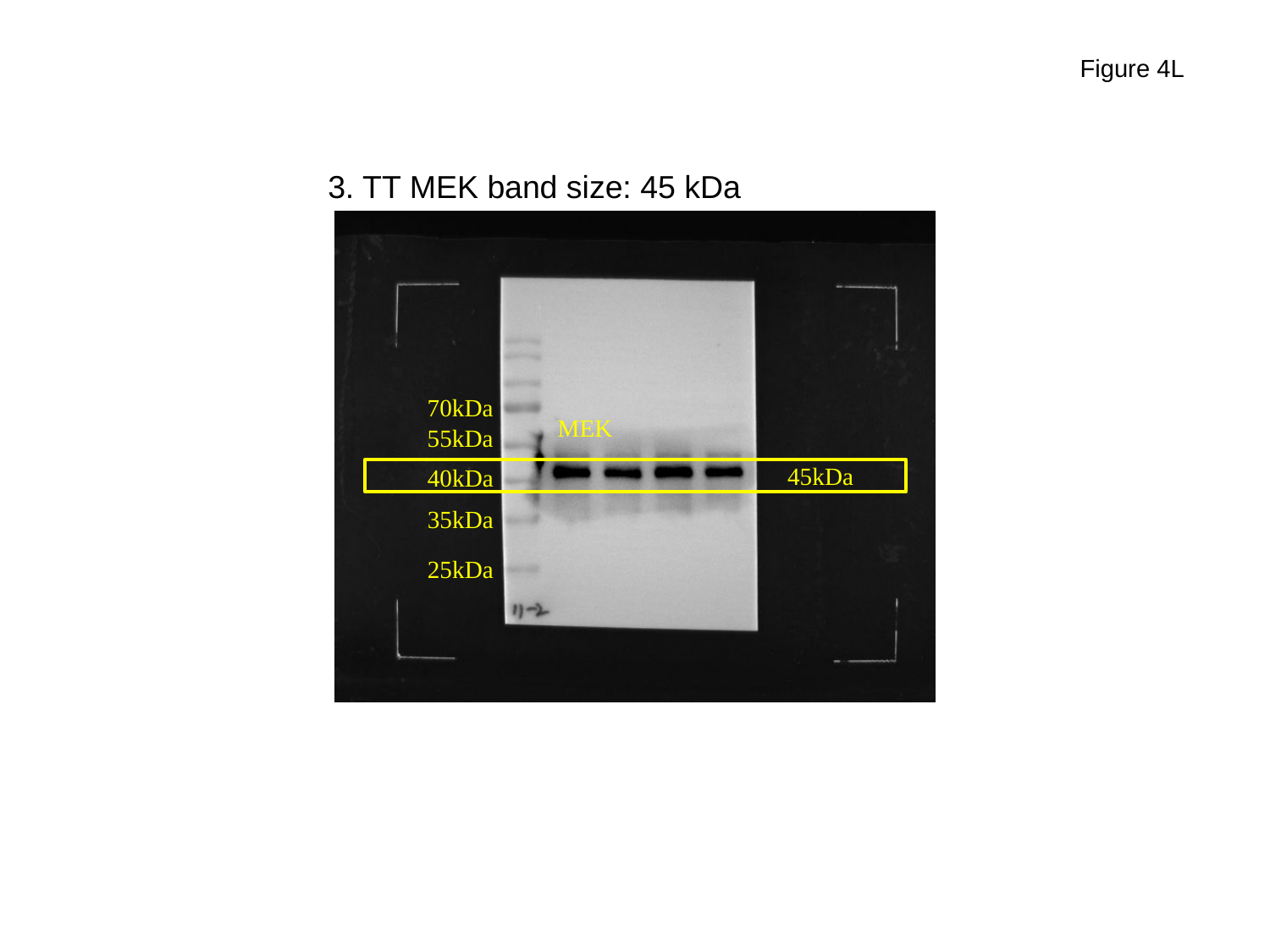

Figure 4L
3. TT MEK band size: 45 kDa
70kDa
MEK
55kDa
45kDa
40kDa
35kDa
25kDa

## Slide 53
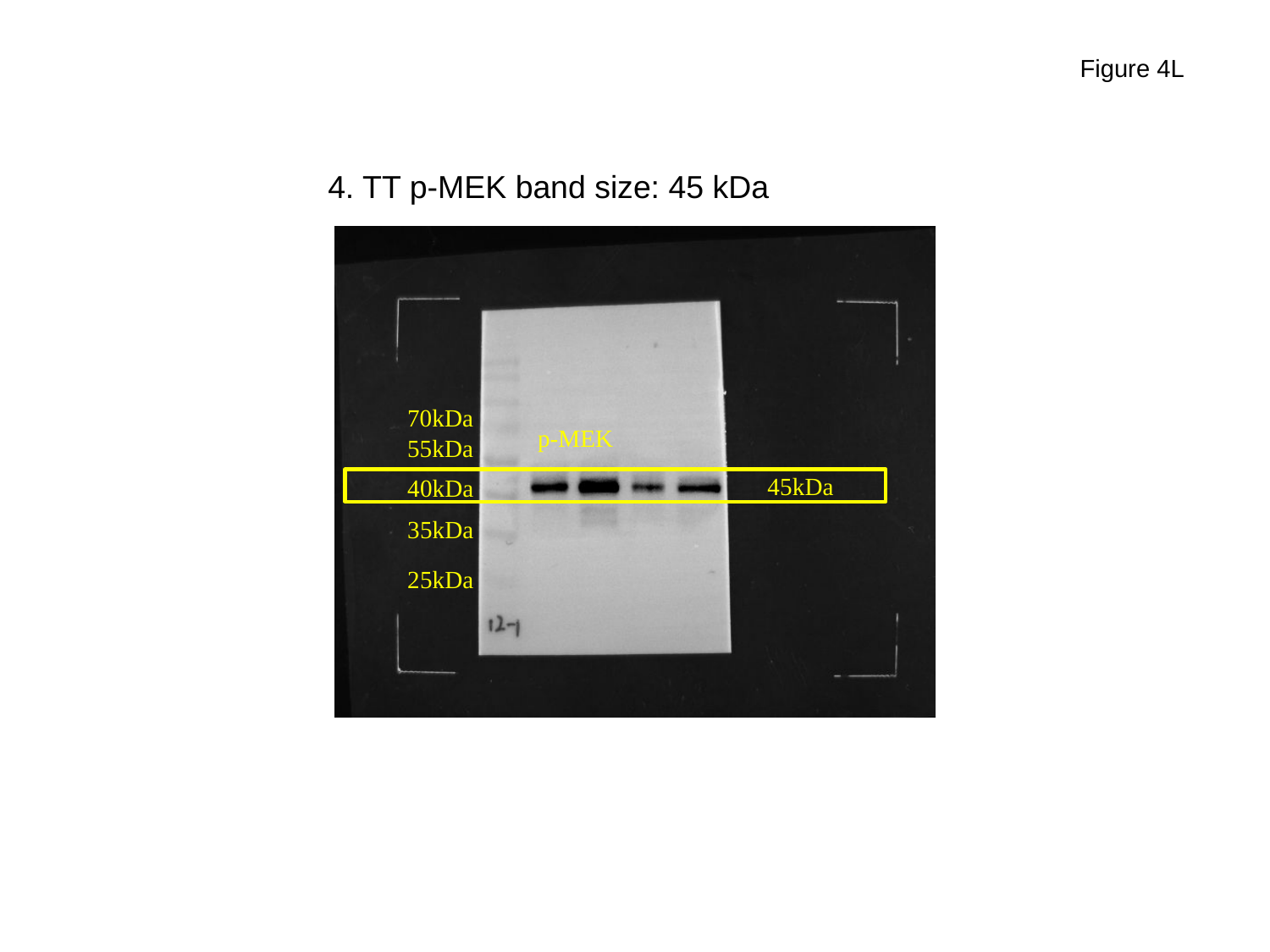

Figure 4L
4. TT p-MEK band size: 45 kDa
70kDa
p-MEK
55kDa
45kDa
40kDa
35kDa
25kDa

## Slide 54
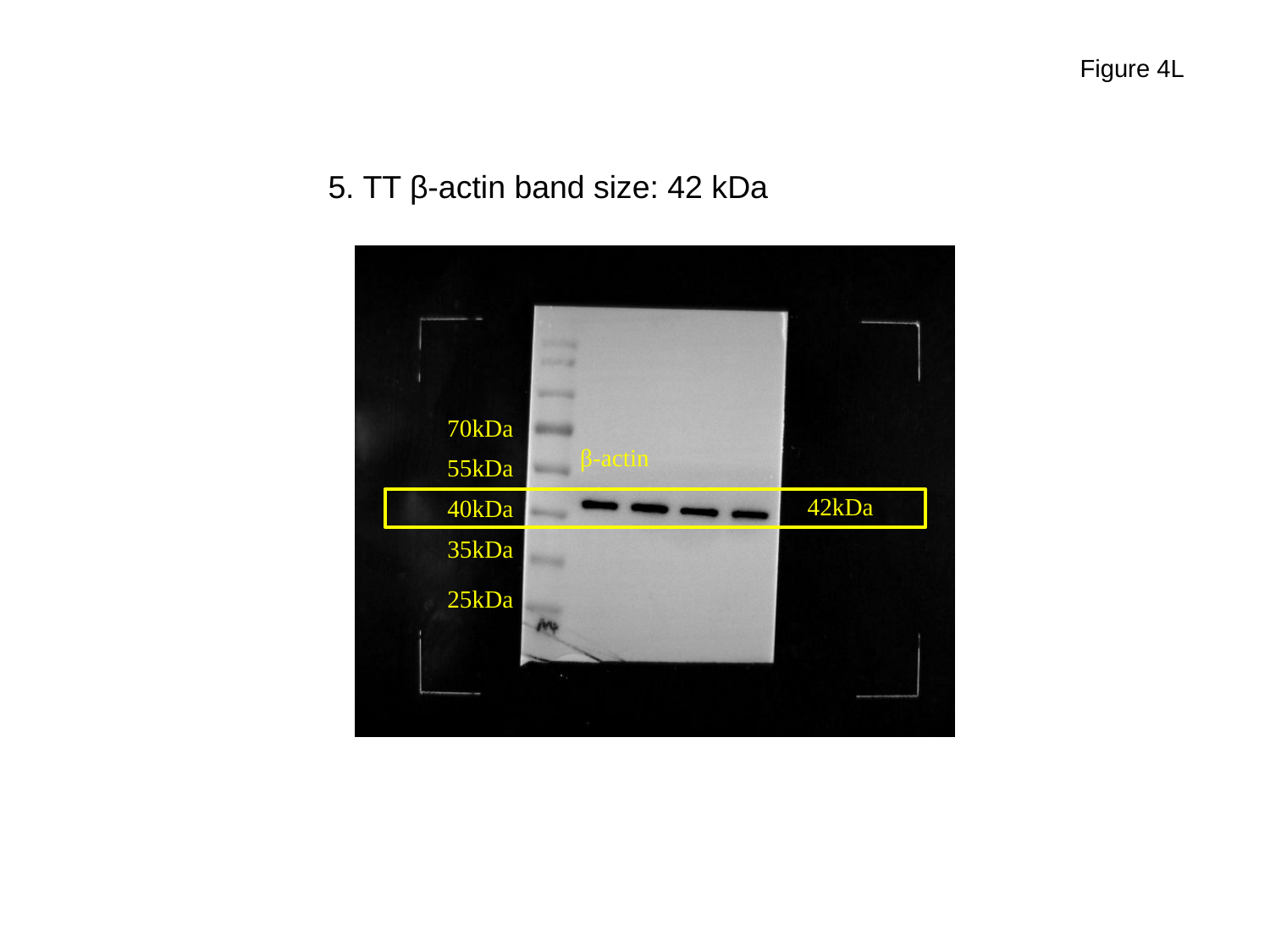

Figure 4L
5. TT β-actin band size: 42 kDa
70kDa
β-actin
55kDa
42kDa
40kDa
35kDa
25kDa

## Slide 55
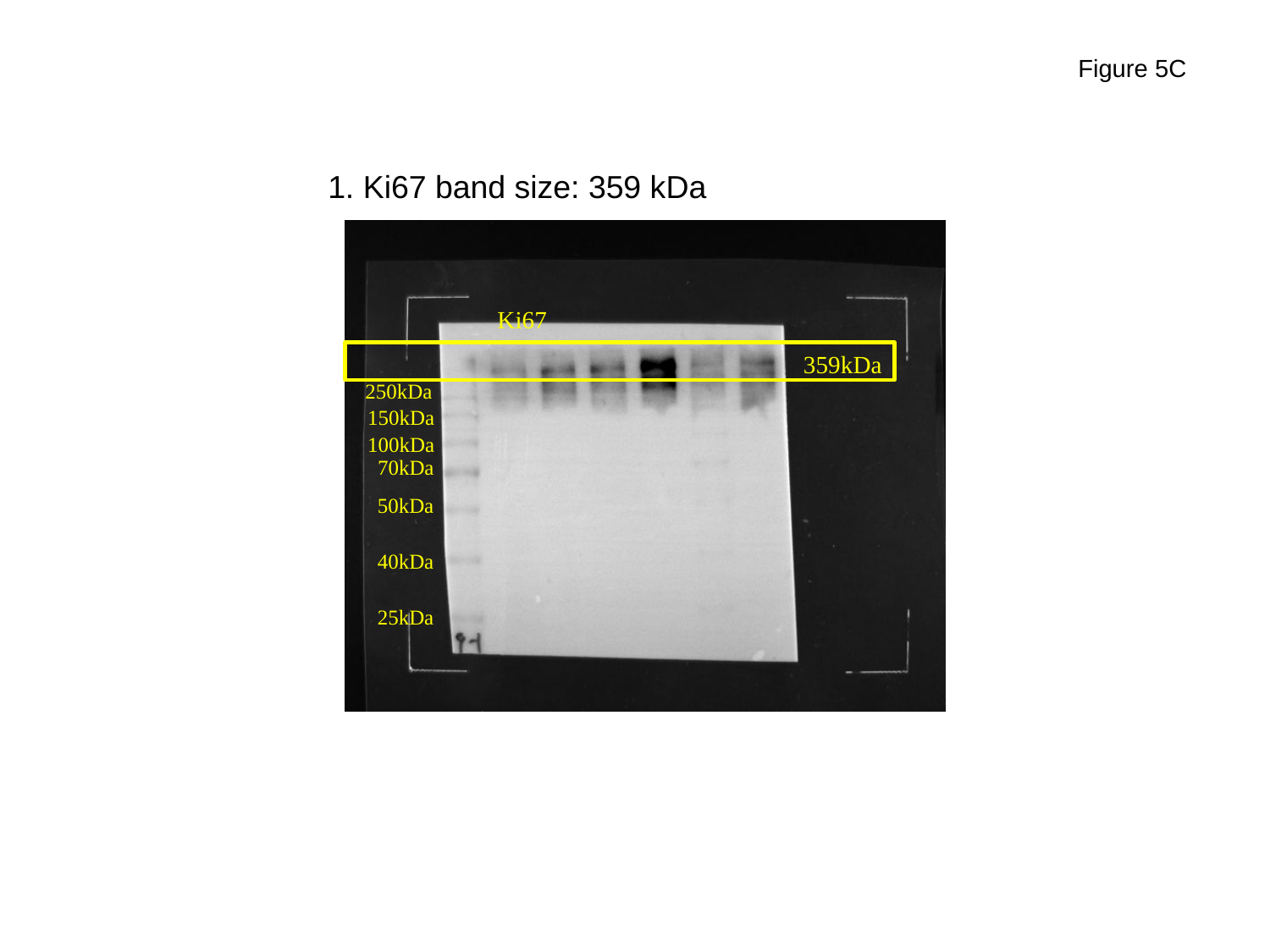

Figure 5C
1. Ki67 band size: 359 kDa
Ki67
359kDa
250kDa
150kDa
100kDa
70kDa
50kDa
40kDa
25kDa

## Slide 56
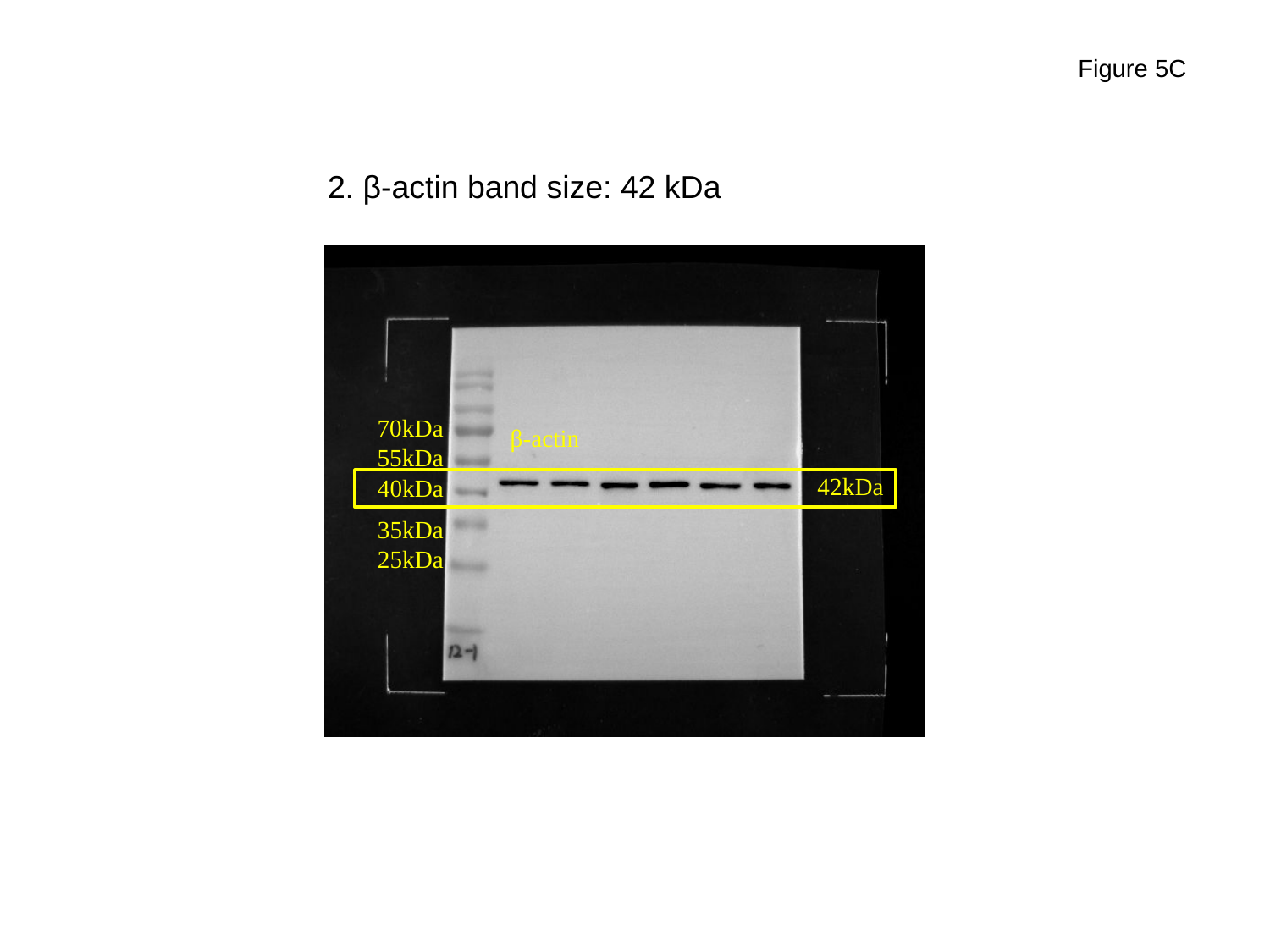

Figure 5C
2. β-actin band size: 42 kDa
70kDa
β-actin
55kDa
42kDa
40kDa
35kDa
25kDa

## Slide 57
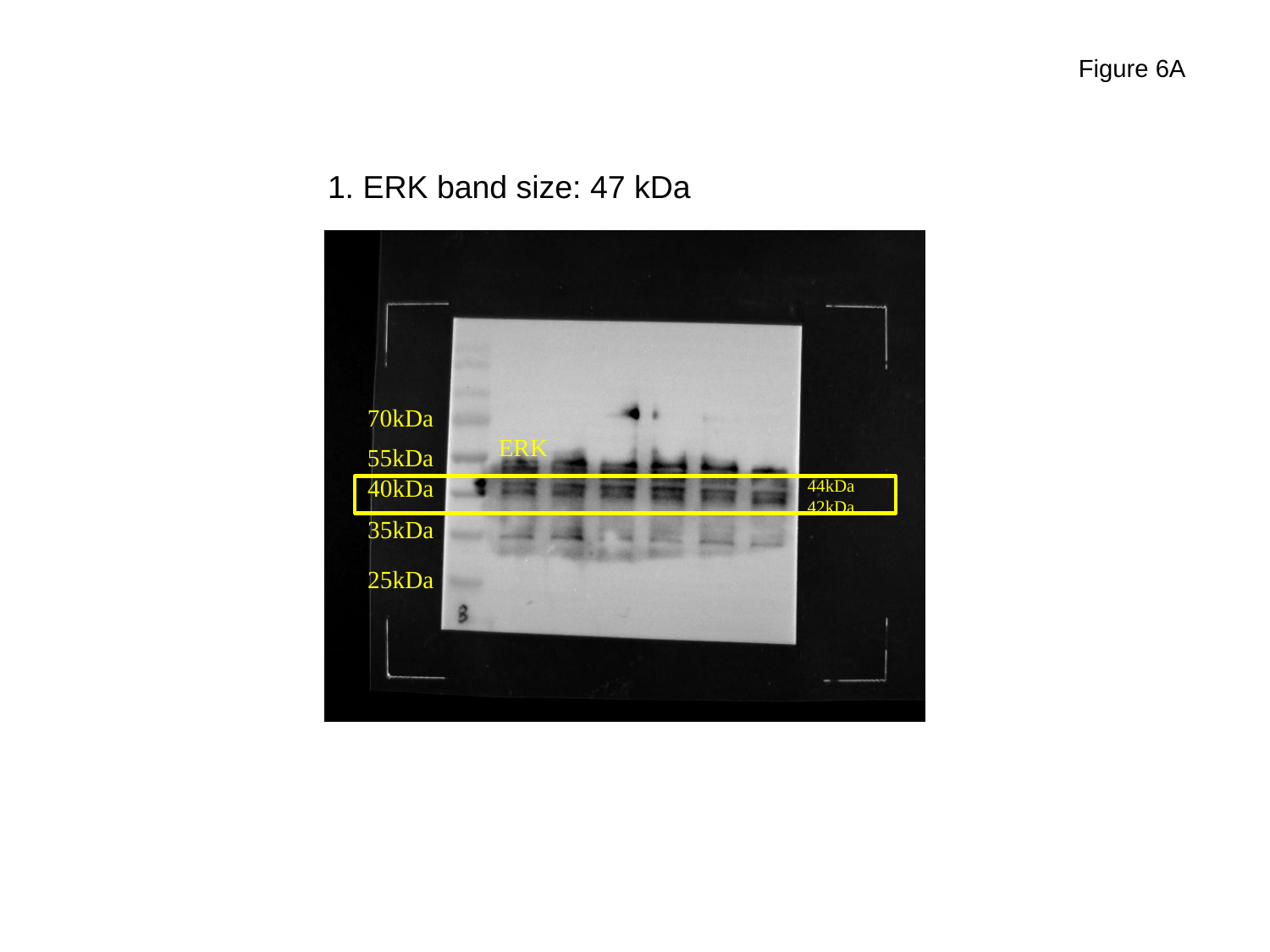

Figure 6A
1. ERK band size: 47 kDa
70kDa
ERK
55kDa
40kDa
44kDa
42kDa
35kDa
25kDa

## Slide 58
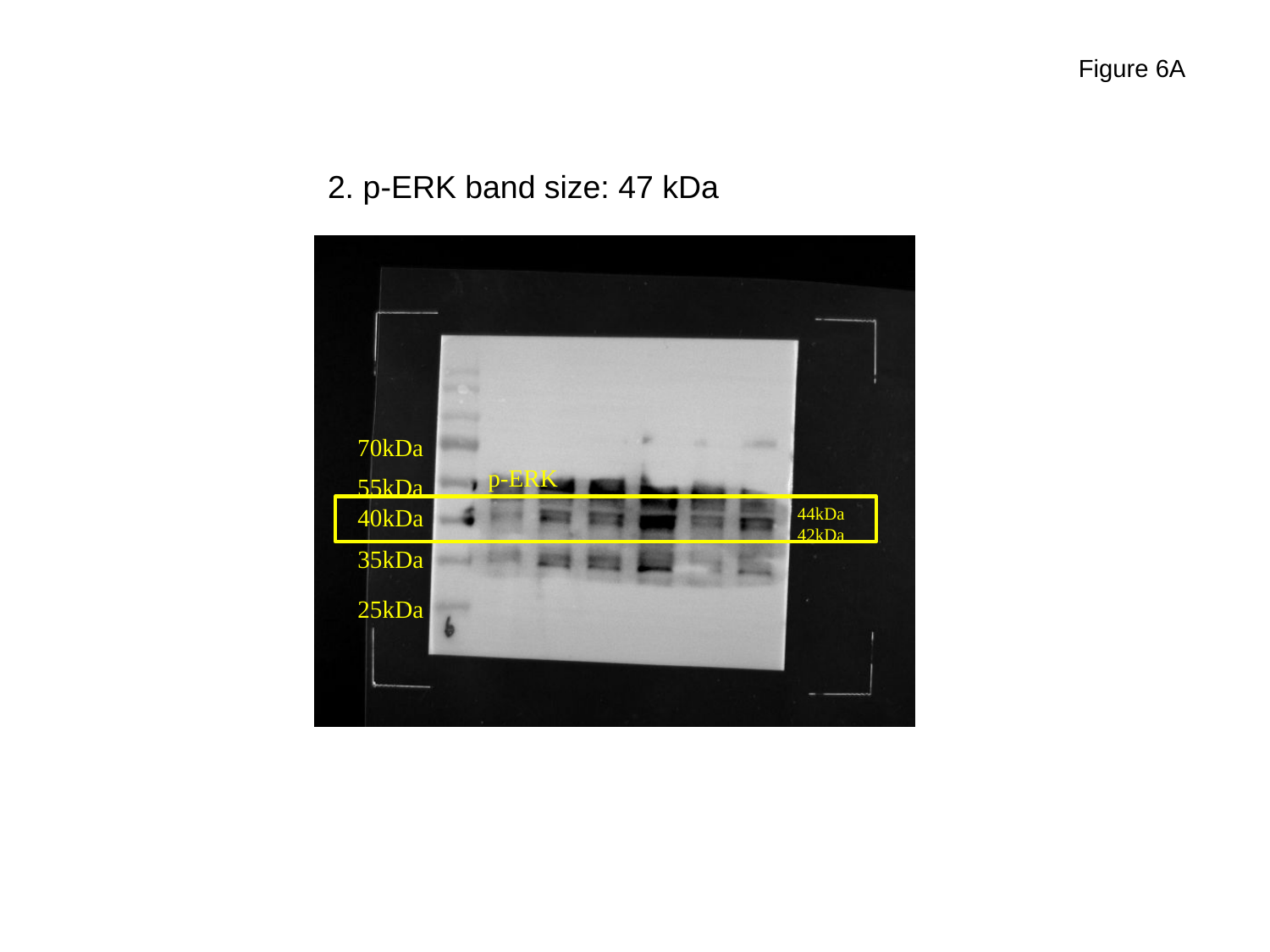

Figure 6A
2. p-ERK band size: 47 kDa
70kDa
p-ERK
55kDa
40kDa
44kDa
42kDa
35kDa
25kDa

## Slide 59
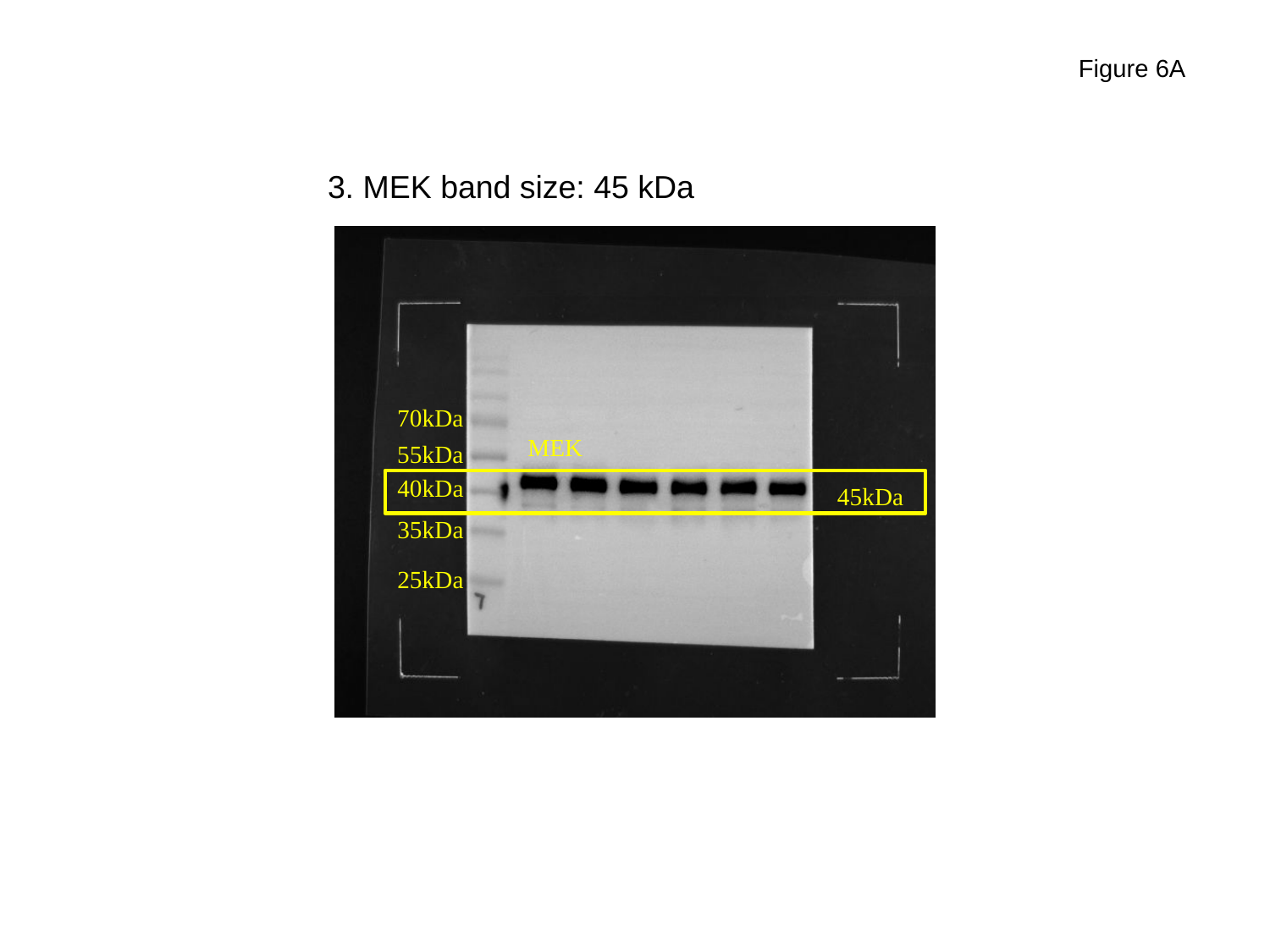

Figure 6A
3. MEK band size: 45 kDa
70kDa
MEK
55kDa
40kDa
45kDa
35kDa
25kDa

## Slide 60
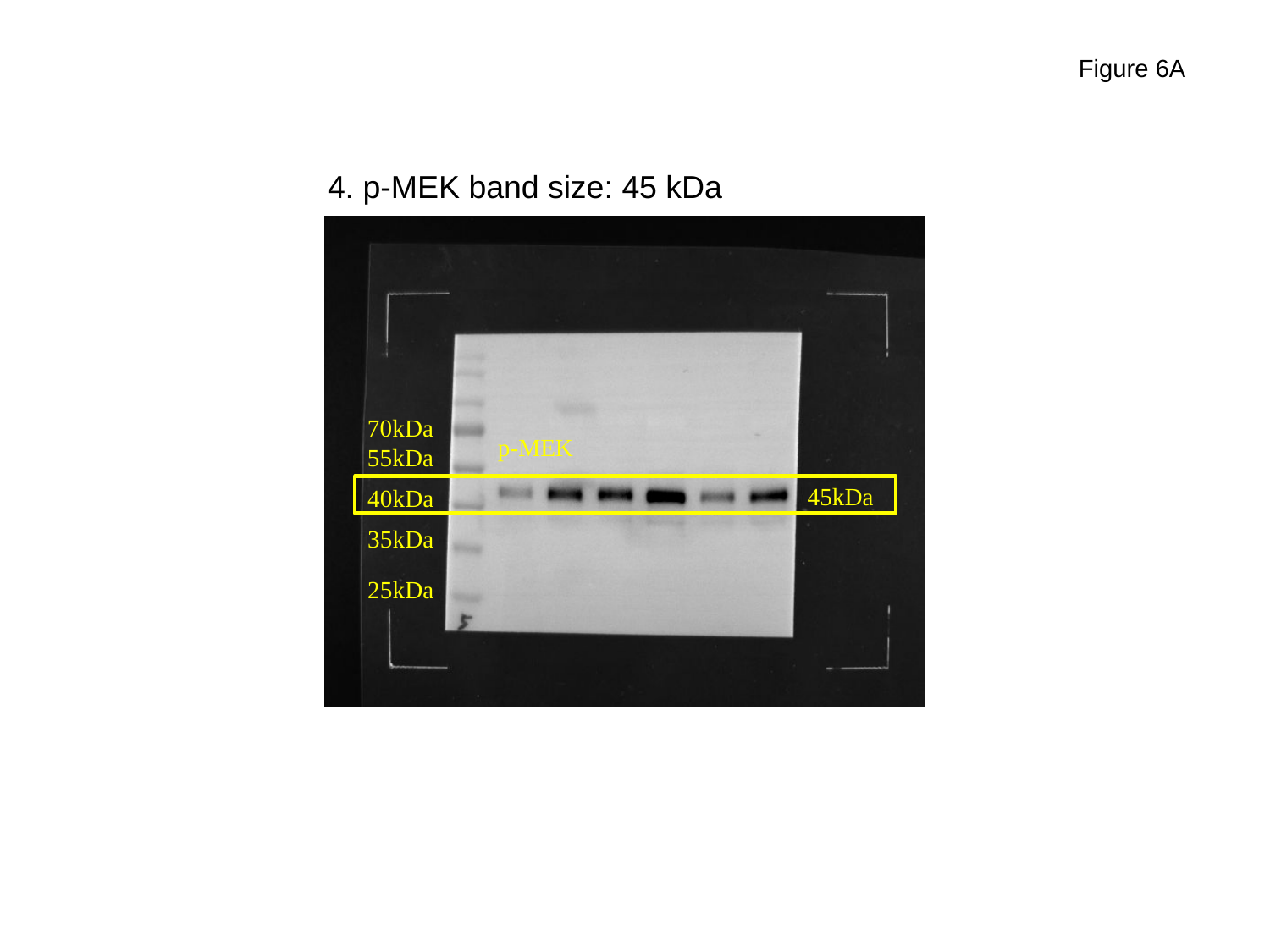

Figure 6A
4. p-MEK band size: 45 kDa
70kDa
p-MEK
55kDa
45kDa
40kDa
35kDa
25kDa

## Slide 61
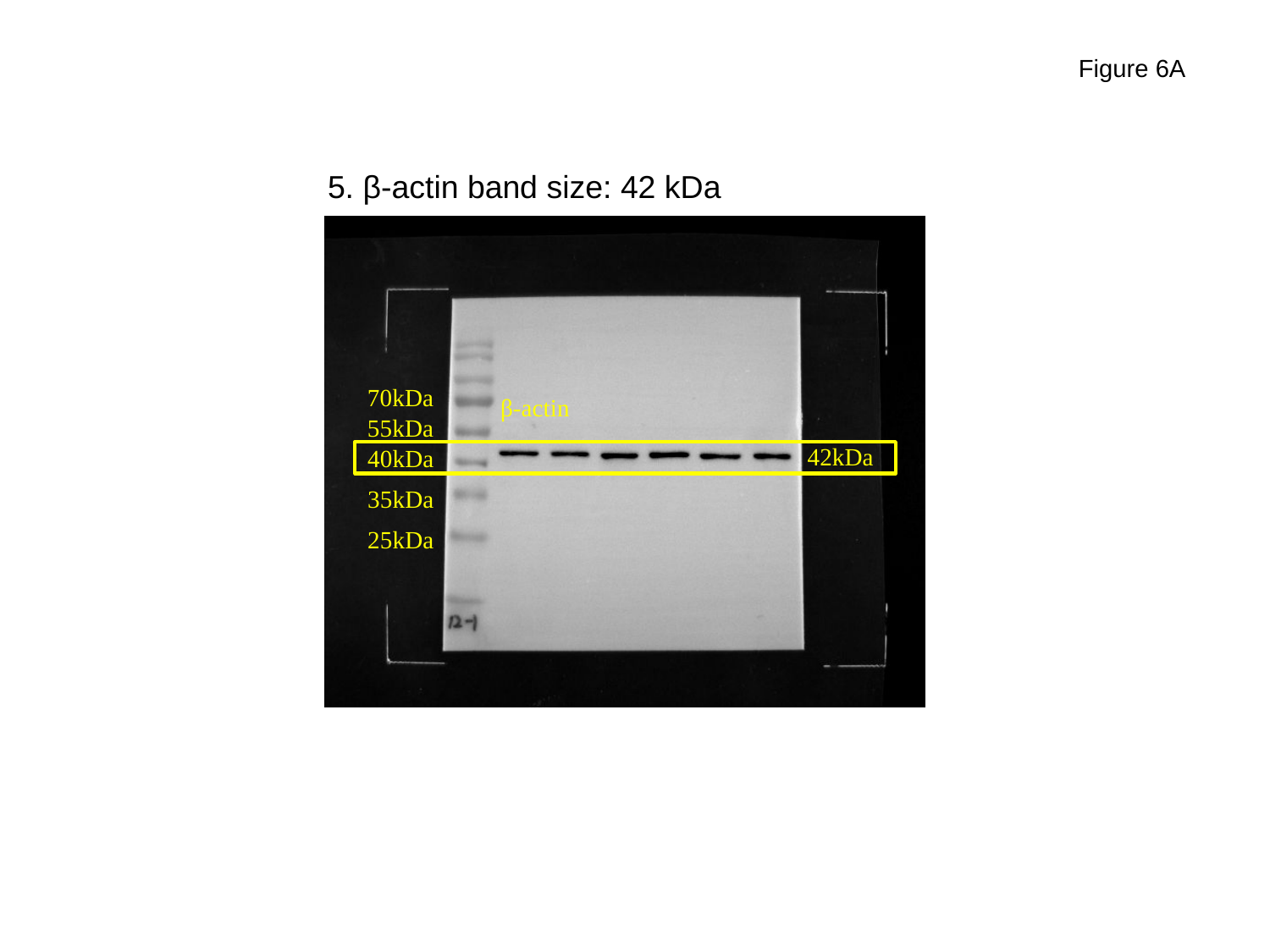

Figure 6A
5. β-actin band size: 42 kDa
70kDa
β-actin
55kDa
42kDa
40kDa
35kDa
25kDa
