# Supplementary material for: Glutamate activates the MAPK pathway by inhibiting LPAR1 expression and promotes anlotinib resistance in thyroid cancer
Source: Discov Oncol. 2025 Jun 13;16:1082. doi: 10.1007/s12672-025-02853-0 (PMC12165934; doi:10.1007/s12672-025-02853-0)
Supplement: Supplementary file 2 — Supplementary Material 2 [file 12672_2025_2853_MOESM2_ESM.pdf]

擎科生物 15368843730 km-tech@tsingke.com.cn(项目)

细胞鉴定实验报告——STR 鉴定

项目报告日： 2024 年 03 月 19 日

实验人	报告人	审核人
张睿宇	张睿宇	张睿宇

TSINGKE

■ 样品信息

1. 样品名称

8305c

2 样品类型

细胞悬浮液

■ 实验方法

1. 提取细胞基因组 DNA

使用 Tsingke 的动物基因组抽提试剂盒（货号 TSP201-200）提取细胞的基因组 DNA。

2. 使用 20 位点特异荧光引物进行扩增

使用新海生物的 NuHi SU9 dNTP Mix（货号 NH9347）进行扩增。

■ 实验结果

样品编号	多等位基因	匹配细胞系	细胞库	匹配度	匹配说明
8305c	无	8305C	ATCC	1.00	完全匹配

- 多等位基因指三等位及以上基因现象。

备注：待测细胞系与收录于 ATCC, DSMZ, JCRB 和 RIKEN 数据库的细胞系 STR 数据进行比对，未收录于以上细胞库的细胞系将无法匹配。

擎科生物 15368843730 km-tech@tsingke.com.cn(项目)

■样本分型结果详情

细胞 8305c 的 STR 位点和 Amelogenin 位点的基因分型结果						
Loci	送检细胞 STR 信息			细胞库细胞 STR 信息		
	送检细胞名:8305c			细胞库细胞名: 8305C		
	Allele1	Allele2	Allele3	Allele1	Allele2	Allele3
D5S818	11	12		11	12	
D13S317	8	11		8	11	
D7S820	10	10		10	10	
D16S539	11	12		11	12	
VWA	17	18		17	18	
TH01	6	9		6	9	
AMEL	X			X		
TPOX	8	11		8	11	
CSF1PO	10	12		10	12	
D12S391	18	21		18	21	
FGA	22	25		22	25	
D2S1338	19	23		19	23	
D21S11	29	30		29	30	
D18S51	12	17		12	17	
D8S1179	11	13		11	13	
D3S1358	15	18		15	18	
D6S1043						
PENTAE	10	12		10	12	
D19S433	13	14.2		13	14.2	
PENTAD	9	10		9	10	
