# Supplementary material for: Glutamate activates the MAPK pathway by inhibiting LPAR1 expression and promotes anlotinib resistance in thyroid cancer
Source: Discov Oncol. 2025 Jun 13;16:1082. doi: 10.1007/s12672-025-02853-0 (PMC12165934; doi:10.1007/s12672-025-02853-0)
Supplement: Supplementary file 3 — Supplementary Material 3 [file 12672_2025_2853_MOESM3_ESM.pdf]

赛库生物 CELLCOOK

产品信息

K1

人甲状腺癌细胞（乳头状）

(CellCook cat: CC2301)

- **形态特征：**上皮细胞样
- **生长特征：**贴壁
- **种属：**人源
- **组织来源：**甲状腺
- **疾病：**甲状腺乳头状癌

规格及存储

常规出库：

T25培养瓶，1x10^{6}cells

活细胞请及时放置于细胞培养箱

(37℃，5%CO_2)

冻存株出库：

同批次2管，1x10^{6}cells/管

接收后请及时存储于液氮（-196℃）

使用范围

本产品仅限于科学研究

- 广州赛库生物技术有限公司
- Guangzhou Cellcook Biotech Co.,Ltd
- Tel:020-89449936
- Email:info@cellcook.com
- www.cellcook.com

赛库公众号

赛库微信小程序

培养条件

DMEM,high glucose(CellCook cat:CM2016,或同配方) 10%胎牛血清(CellCook cat:CM1002L,或更高级别)

推荐培养试剂

基础培养基：

DMEM,high glucose(CellCook cat:CM2016,或同配方)

血清：

南美胎牛血清(CellCook cat:CM1002L)

添加剂：

\

配套完全培养基(CellCook cat:CC2301M)

传代比例：不高于1:3传代（培养面积比）

传代方式：消化2分钟

换液频率：每周换液2-3次

倍增时间：24 hours (PubMed=9054892).

冻存液配方：DMEM,high glucose+10%FBS+10%DMSO

难度等级：+

培养要点：暂无

特征特性：从原发性乳头状甲状腺癌中分离建系，细胞维持甲状腺滤泡细胞分化，如合成甲状腺球蛋白。细胞表达野生型p53肿瘤抑制基因。文献报道K1细胞来源于甲状腺细胞系GLAG-66。

STR位点信息：

STR Profile	AMEL	CSF1PO	D13S317	D16S539	D5S818	D7S820	TH01	TPOX	vWA
K1	X,Y	11,12	11,14	11,12	10,11	11	6,9	8	17,18
